# Unveiling the Antimycobacterial Potential of Novel 4-Alkoxyquinolines: Insights into Selectivity, Mechanism of Action, and *In Vivo* Exposure

**DOI:** 10.1021/acs.jmedchem.4c01302

**Published:** 2024-12-04

**Authors:** Fernanda Fries da Silva, Josiane Delgado Paz, Raoní Scheibler Rambo, Guilherme Arraché Gonçalves, Mauro Neves Muniz, Alexia de Matos Czeczot, Marcia Alberton Perelló, Andresa Berger, Laura Calle González, Lovaine Silva Duarte, Anelise Baptista da Silva, Carlos Alexandre Sanchez Ferreira, Sílvia Dias de Oliveira, Sidnei Moura, Cristiano Valim Bizarro, Luiz Augusto Basso, Pablo Machado

**Affiliations:** a Instituto Nacional de Ciência e Tecnologia em Tuberculose, Centro de Pesquisas em Biologia Molecular e Funcional, Pontifícia Universidade Católica do Rio Grande do Sul, Porto Alegre, Rio Grande do Sul 90616-900, Brazil; b Programa de Pós-Graduação em Biologia Celular e Molecular, Pontifícia Universidade Católica do Rio Grande do Sul, Porto Alegre, Rio Grande do Sul 90616-900, Brazil; c Programa de Pós-Graduação em Medicina e Ciências da Saúde, Pontifícia Universidade Católica do Rio Grande do Sul, Porto Alegre, Rio Grande do Sul 90616-900, Brazil; d Laboratório de Imunologia e Microbiologia, Pontifícia Universidade Católica do Rio Grande do Sul, Porto Alegre, Rio Grande do Sul 90616-900, Brazil; e Laboratório de Biotecnologia de Produtos Naturais e Sintéticos, Instituto de Biotecnologia, Universidade de Caxias do Sul, Caxias do Sul, Rio Grande do Sul 95070-560, Brazil

## Abstract

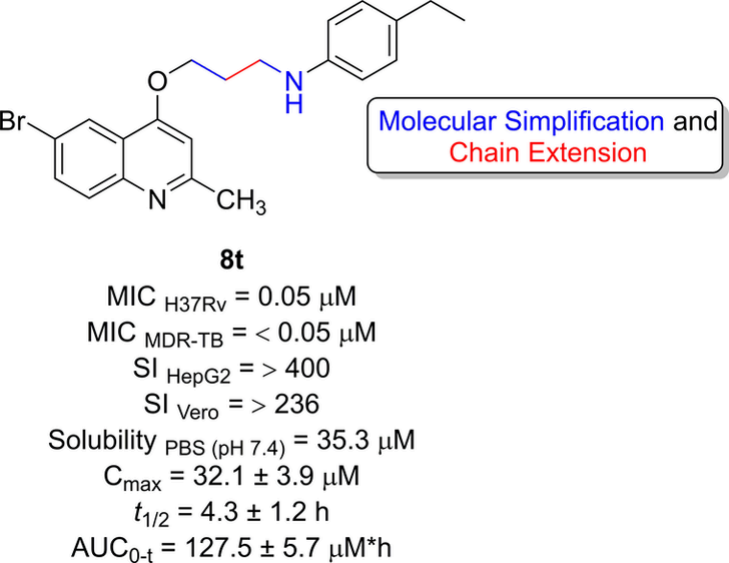

This work presents a comprehensive investigation into the design, synthesis, and evaluation of a novel series of 4-alkoxyquinolines as potential antimycobacterial agents. The design approach, which combined molecular simplification and chain extension, resulted in compounds with potent and selective activity against both drug-susceptible and multidrug-resistant *Mycobacterium tuberculosis* strains. The lead molecule, targeting the cytochrome *bc*_1_ complex, exhibited favorable kinetic solubility and remarkable chemical stability under acidic conditions. Despite *in vitro* ADME evaluations showing low permeability and high metabolism in rat microsomes, the lead compound exhibited bacteriostatic activity in a murine macrophage model of TB infection and demonstrated promising *in vivo* exposure following gavage in mice, with an AUC_0–*t*_ of 127.5 ± 5.7 μM h. To the best of our knowledge, for the first time, a simplified structure from 2-(quinolin-4-yloxy)acetamides has shown such potential. These findings suggest a new avenue for exploring this chemical class as a source of antituberculosis drug candidates.

## 1 Introduction

Tuberculosis (TB), caused by *Mycobacterium tuberculosis* (Mtb), has been recognized as a global public health crisis by the World Health Organization (WHO) since the 1990s. In 2022, the WHO reported 10.6 million TB cases and 1.3 million deaths worldwide, making TB the second leading cause of death from a single infectious agent, following SARS-CoV-2.^1^ The disease primarily affects the lungs but can also impact other parts of the body, causing severe health complications. Despite significant advances in TB research and treatment, the disease remains a major health challenge, especially in low- and middle-income countries.^1^ Unfortunately, the COVID-19 pandemic has disrupted access to diagnosis, treatment, and public health efforts, potentially worsening the TB burden and jeopardizing years of progress in TB control.^1^

The primary pharmacological approach to treating TB involves the use of drugs such as isoniazid (INH), rifampicin (RIF), pyrazinamide, and ethambutol.^1^ Despite the efficacy of this recommended treatment regimen, the emergence of mono-, multi-, and polydrug-resistant strains has complicated TB management, leading to increased morbidity and mortality. Alarmingly, the WHO reports that a significant proportion of patients with drug-resistant TB have not received proper diagnosis and treatment.^2^ Inadequate healthcare infrastructure, limited access to diagnostics, and a lack of effective drugs have been significant barriers to optimal clinical management. In response to these growing resistance challenges, the 21st century has seen the approval of three new anti-TB drugs: bedaquiline (2012),^3^ delamanid (2014),^4^ and pretomanid (2019),^5^ aimed at combating strains that are unresponsive to traditional treatments. It is noteworthy that pretomanid was approved in combination with bedaquiline and linezolid for the treatment of a specific subset of adult patients with extensively drug-resistant TB.^5^ These new drugs represent a critical advancement in TB therapy, offering hope for improved outcomes in patients with drug-resistant TB. However, the implementation of these new agents has required careful monitoring to prevent the development of resistance. Treatment failures and genotypic changes associated with resistance have already been documented for bedaquiline and delamanid.^6,7^ Therefore, continuous research and development, along with global collaboration, are essential to address the evolving threat of drug-resistant TB and ensure effective treatment for all patients.

**Figure 1:**
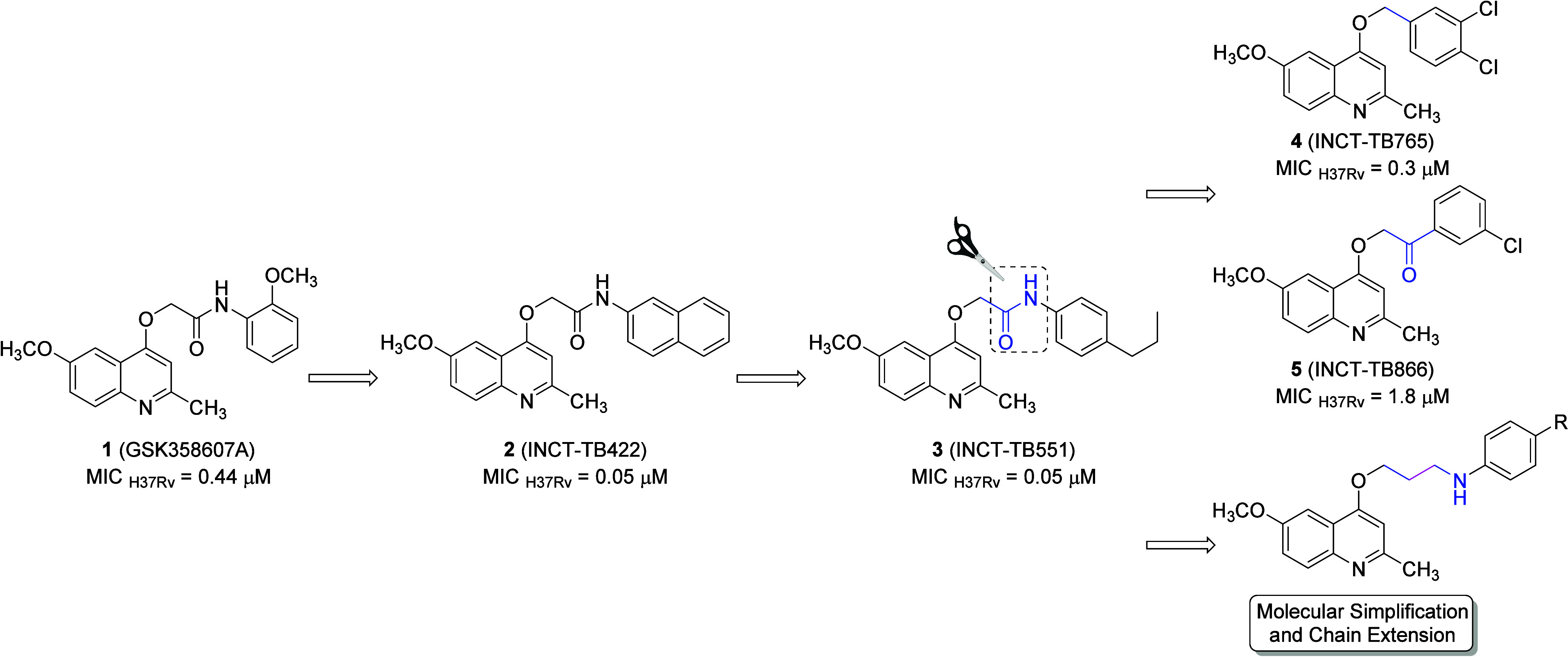
Scaffold evolution starting from 2-(quinolin-4-yloxy)acetamide **1**, discovered through high-throughput screening, led to compounds **2** and **3**, which showed optimized antimycobacterial activity. Molecules **4** and **5** were derived by molecular simplification of the amide function and exhibited a significant reduction in potency. Finally, the innovative design of 4-alkoxyquinolines, combining molecular simplification with chain extension explored in this study.

Within this context, our research group has focused on the design and synthesis of compounds with the potential to inhibit the growth of both drug-susceptible and drug-resistant Mtb strains.^8−15^ Among the chemical classes explored, 2-(quinolin-4-yloxy)acetamides have demonstrated potent and selective activity,^8−11^ exerting their inhibitory effect by targeting the cytochrome *bc*_1_ complex.^10,11,16^ This class of compounds was discovered through high-throughput screening, leading to the identification of molecule **1**, which inhibited Mtb growth with a minimum inhibitory concentration (MIC) of 0.44 μM (Figure 1).^17^ As part of our ongoing research program, this chemical class has been optimized using classical techniques in medicinal chemistry. Modifications aiming the improvement of liposolubility of the acetamide group resulted in compound **2**, which exhibited a significantly enhanced ability to inhibit Mtb, with an MIC of 0.05 μM (Figure 1).^8^ Further structural variations revealed that the potency of the 2-(quinolin-4-yloxy)acetamide scaffold was not dependent on the planarity of the amide substituent. Notably, compound **3**, featuring a propyl group attached to the 4-position of the benzene ring, maintained an MIC of 0.05 μM while offering significant improvements in aqueous solubility and metabolic stability (Figure 1).^9^ Interestingly, while the presence of the amide function attached at the 4-position of the quinoline ring has been described as a vulnerable point for hydrolysis reactions, leading to potential stability issues,^18^ its absence has resulted in compounds with significantly reduced activity against Mtb.^13,18^ In our study, the most potent simplified molecules, **4** and **5**, presented MIC values of 0.3 μM and 1.8 μM, respectively (Figure 1).^13^ This represents a 6- to 36-fold decrease in potency compared to 2-(quinolin-4-yloxy)acetamides **2** and **3**, suggesting that the amide group plays a critical role in the bioactivity of these compounds. To enhance the stability and efficacy of these agents, further structural modifications and optimization strategies has been investigated. One of our experimental approaches involved introducing electron-donating groups to strategically reduce the electrophilicity of the amide carbonyl, thereby mitigating hydrolysis-mediated reactions.^10^ Encouragingly, the results indicate enhanced metabolic stability of the designed compounds while maintaining their inhibitory potency against the bacillus.

In this study, the proposed molecules were designed by excluding the amide group while simultaneously extending the side chain through the incorporation of alkyl groups (Figure 1). This strategic alteration aimed to access the hydrophobic regions of the potential molecular target. As previously demonstrated through molecular docking, 2-(quinolin-4-yloxy)acetamides interact with the β-subunit of the cytochrome *bc*_1_ complex, specifically engaging residues T313, M342, and L176.^16^ Notably, the alkyl and aryl substituents positioned at the quinoline ring’s 4-position were oriented toward a substantial hydrophobic region within the protein, forming van der Waals interactions with the L176 residue. This site could facilitate additional hydrophobic interactions, enhancing binding affinity through closer contacts. It is noteworthy that molecular simplification performed keeping the side chain size did not result in molecules as potent as 2-(quinolin-4-yloxy)acetamides.^13,18^ Based on Structure–Activity Relationship (SAR) data, the antimycobacterial activity of this chemical class has been enhanced in the presence of bulky and hydrophobic substituents adjacent to the 4-position of the heterocycle.^8−11,18,19^ Thus, we hypothesized that elongating the side chain could facilitate optimal positioning of hydrophobic groups, potentially leading to improved antimycobacterial activity by enhancing intermolecular interactions. Therefore, a new series of 4-alkoxyquinolines featuring extended side chains was synthesized and evaluated against the drug-susceptible *M. tuberculosis* H37Rv strain. The basic structural requirements for compound potency (SAR) were determined using MIC values. The most promising structures were further tested against a panel of multidrug-resistant strains. Additionally, the viability of HepG2 and Vero cells was assessed to provide preliminary indications of molecular toxicity and selectivity. The possible mechanism of action of these compounds was investigated using a spontaneous mutant strain.^16^ Further insights were obtained through assessments of kinetic solubility, chemical stability, passive permeability, metabolic stability, intracellular activity and *in vivo* absorption profiling of the most promising compound.

## 2 Results and Discussion

**Scheme 1:**
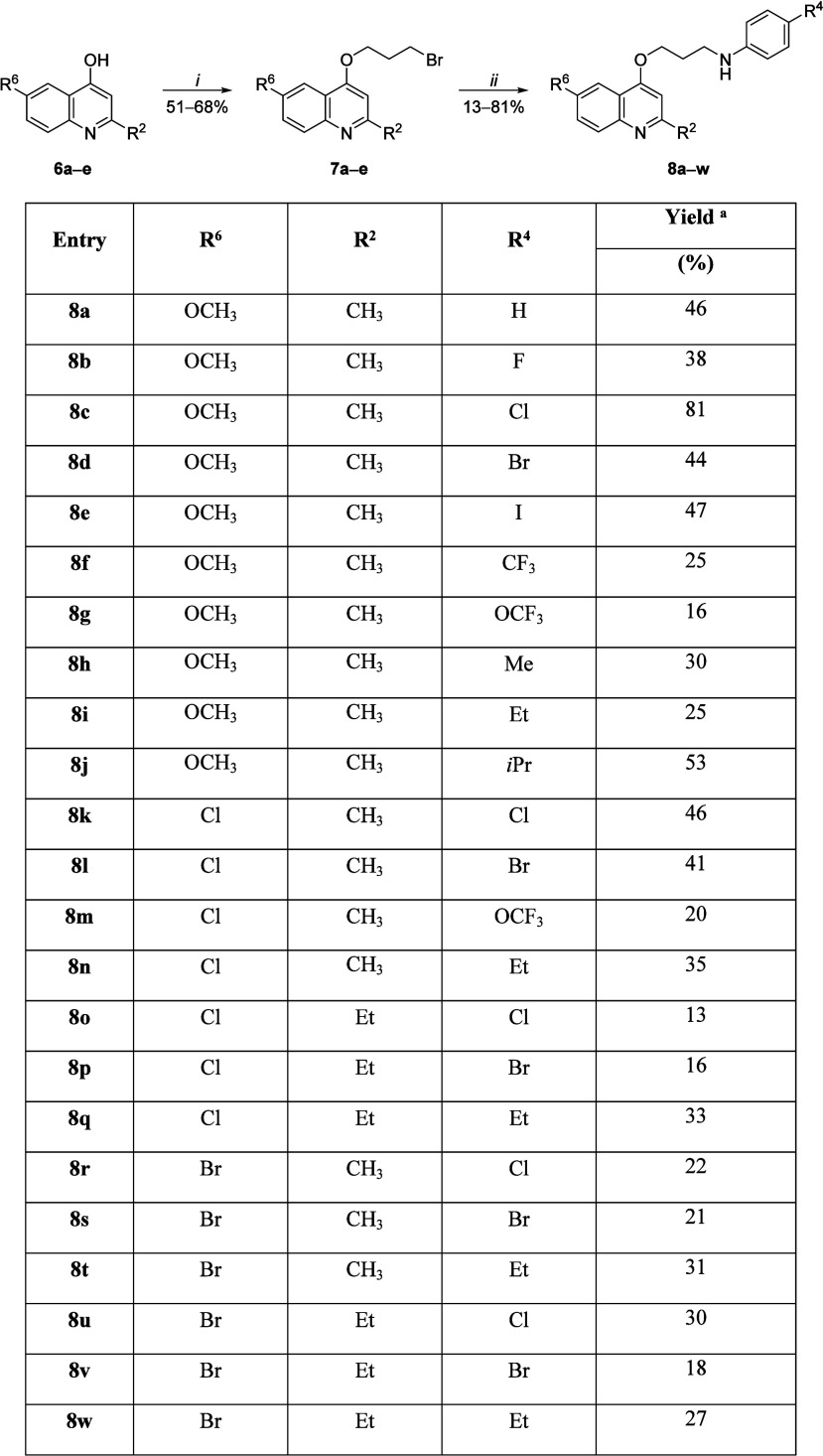
Conditions: *i* = 1,3-Dibromopropane, Cs_2_CO_3_, NaI, CH_3_CN, 25 °C, 24 h; *ii* = Anilines, K_2_CO_3_, NaI, toluene, 110 °C, 48-72 h Yields were quantified after purification and were not optimized.

The designed compounds were synthesized in two alkylation steps from the 4-hydroxyquinolines (**6a**–**e**). The *O*-alkylation of the **6a**–**e** was carried out using 1,3-dibromopropane as the alkylating agent in the presence of cesium carbonate (Cs_2_CO_3_) and sodium iodide (NaI). Reactants and reagents were stirred in acetonitrile for 24 h at 25 °C to give the desired products **7a**–**e** in 51–68% yields (Scheme 1). Although quinolinic nitrogen alkylation may be also obtained,^8^ only the *O*-alkylation product was observed in the reactions performed under the described experimental conditions. In the second step, the alkylation of appropriately substituted anilines was carried out in the presence of potassium carbonate (K_2_CO_3_) and NaI using toluene as solvent. The reaction mixture was heated at 110 °C for 48–72 h to provide structures **8a**–**w** in 13–81% yields after purification (Scheme 1). All compounds presented spectroscopic and spectrometric data consistent with the proposed chemical structures (Supporting Information).

Synthesized 4-alkoxyquinolines (**8a**–**w**) were evaluated in a whole-cell assay for their ability to inhibit the *M. tuberculosis* H37Rv strain using isoniazid, rifampicin, and telacebec (Q203) as positive control.^20^ In this experiment, the MIC was defined as the lowest concentration of the compound capable of preventing colorimetric alteration on resazurin-based assay. Overall, the molecules **8a**–**w** showed good to excellent antimycobacterial activities with MICs values ranging from 12.32 μM to 0.03 μM under the evaluated experimental conditions (Table 1).

**Table 1:**
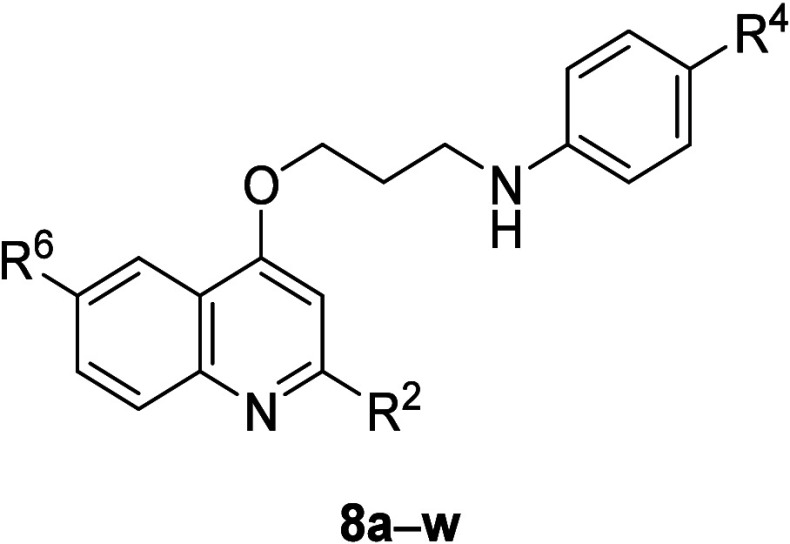
*In Vitro* Activities of the 4-Alkoxyquinolines against the *M.tuberculosis* H37Rv Strain

				**MIC (H37Rv)**^a^
**Entry**	**R**^**6**^	**R**^**2**^	**R**^**4**^	**(μM)**
**8a**	OCH_3_	CH_3_	H	0.25
**8b**	OCH_3_	CH_3_	F	0.47
**8c**	OCH_3_	CH_3_	Cl	0.22
**8d**	OCH_3_	CH_3_	Br	0.10
**8e**	OCH_3_	CH_3_	I	0.09
**8f**	OCH_3_	CH_3_	CF_3_	3.20
**8g**	OCH_3_	CH_3_	OCF_3_	0.20
**8h**	OCH_3_	CH_3_	Me	0.24
**8i**	OCH_3_	CH_3_	Et	0.06
**8j**	OCH_3_	CH_3_	*i*Pr	0.11
**8k**	Cl	CH_3_	Cl	0.22
**8l**	Cl	CH_3_	Br	1.55
**8m**	Cl	CH_3_	OCF_3_	0.19
**8n**	Cl	CH_3_	Et	0.03
**8o**	Cl	Et	Cl	0.11
**8p**	Cl	Et	Br	0.38
**8q**	Cl	Et	Et	0.22
**8r**	Br	CH_3_	Cl	0.10
**8s**	Br	CH_3_	Br	0.35
**8t**	Br	CH_3_	Et	0.05
**8u**	Br	Et	Cl	0.19
**8v**	Br	Et	Br	0.34
**8w**	Br	Et	Et	0.10
**INH**	-	-	-	2.3
**RIF**	-	-	-	0.2
**Q203**	-	-	-	0.009

a Minimum inhibitory concentration (MIC) against the *M. tuberculosis* H37Rv strain. INH, isoniazid. RIF, rifampin. Q203, telacebec.

Notably, the unsubstituted product **8a**, obtained from the reaction with aniline, was able to inhibit the bacillus with MIC value of 0.25 μM. This preliminary finding denoted that molecular simplification combined with chain extension could lead to structures with high activity against Mtb. The introduction of a fluorine atom at the 4-position of the benzene ring led to an approximate 2-fold reduction in activity, as evidenced by the 4-alkoxyquinoline **8b** with a MIC of 0.47 μM. In contrast, the incorporation of chlorine (**8c**), bromine (**8d**), and iodine (**8e**) at this part of the compound, resulting in increased atomic volume and polarizability, correlated with improved antimycobacterial activity. Indeed, molecules **8c**, **8d**, and **8e** exhibited MIC values of 0.22 μM, 0.10 μM, and 0.09 μM, respectively, reflecting an enhancement of roughly 2-fold to more than 5-fold when compared to activity of the fluorinated structure **8b**. The presence of a trifluoromethyl group attached to the 4-position of the benzene ring significantly reduced the inhibitory activity of compound **8f**. This electron-withdrawing group yielded a molecule with a MIC value of 3.20 μM. Interestingly, the introduction of an oxygen atom, forming the trifluoromethoxy group in structure **8g**, restored potency, resulting in a MIC of 0.20 μM. This observation could be attributed to a more effective positioning of the trifluoromethyl group, rather than its electronic characteristics. With the inclusion of oxygen, the trifluoromethyl group deviates from the main plane of the benzene ring, possibly contributing to the heightened inhibitory capacity of the trifluoromethoxy-substituted compound **8g**. The incorporation of a methyl group at the 4-position of the benzene ring was well-tolerated, exhibiting no substantial alteration in antimycobacterial activity. The methylated compound **8h** inhibited the bacilli with a MIC of 0.24 μM, a value comparable to that observed for unsubstituted (**8a**), chlorinated (**8c**), and trifluoromethoxylated (**8g**) molecules. The increase of the alkyl chain with the presence of an ethyl group resulted in the creation of a structure endowed with potent activity against Mtb. The 4-alkoxyquinoline **8i** demonstrated a MIC of 0.06 μM, which was 4-fold more potent than methyl-containing analogue **8h**. Remarkably, for the first time, a simplified analogue derived from 2-(quinoline-4-yloxy)acetamides exhibited a MIC within the submicromolar range. In contrast, the introduction of branching in the alkyl substituent, using an iso-propyl group, diminished the potency of compound **8j**, leading to a MIC value of 0.11 μM.

In the second round of structural modifications, chlorine, bromine, trifluoromethoxy and ethyl groups were fixed as substituents at the 4-position aniline ring. These substituents generated molecules displaying significant activity against the bacillus, forming a collection with diverse steric, electronic, and physicochemical properties. It is noteworthy that while iodine yielded a derivative with substantial inhibitory capacity, bromine was selected for subsequent modifications due to its comparable outcomes. Alteration of the methoxy group attached at 6-position of the quinoline ring by a chlorine resulted in the maintenance of inhibitory potency against *M. tuberculosis* H37Rv strain. Specifically, structure **8k** effectively reduced the bacilli exhibiting a MIC of 0.22 μM, a value identical to that exhibited by its methoxylated counterpart **8c**. In contrast, when chlorine replaced the methoxy group in the 4-alkoxyquinoline **8l**, a considerable decline in activity was observed leading to a MIC of 1.55 μM. This value denoted a 15.5-fold decrease in potency when comparing compound **8l** with molecule **8d**. The incorporation of chlorine at the 6-position of the quinoline ring and trifluoromethoxy as a side chain attachment in structure **8m** resulted in the restoration of potency as this 4-alkoxyquinoline showed MIC of 0.19 μM against Mtb. Additionally, when ethyl substituted the trifluoromethoxy group in the compound **8n**, a significant enhancement in antimycobacterial activity was observed. Molecule **8n** displayed a MIC value of 0.03 μM, representing an increase of over 6-fold in activity when compared to trifluoromethoxy-substituted **8m**. Furthermore, the exchange of the methoxy group at the 6-position of the heterocycle in **8i** with a chlorine atom in **8n** led to a molecule that was 2-fold more potent. The elongation of the alkyl chain attached to the 2-position of the quinolinic ring, with the introduction of an ethyl group, improved the potency of structures **8o** and **8p** compared to their methyl-substituted analogues, **8k** and **8l**. Notably, these 4-alkoxyquinolines demonstrated MIC values of 0.11 μM and 0.38 μM for **8o** and **8p**, respectively, while compounds **8k** and **8l** displayed values of 0.22 μM and 12.32 μM, respectively. Conversely, the inclusion of an ethyl group at the 2-position of the heterocycle led to a reduction in the activity of molecule **8q** by more than 7-fold in comparison to the methylated analogue **8n**. Specifically, structure **8q** exhibited a MIC value of 0.22 μM, whereas **8n** inhibited the growth of the bacilli with a MIC of 0.03 μM.

Subsequently, in a third round of structural modifications, bromine was introduced to substitute chlorine at the 6-position of the quinoline ring. Once again, for the sake of comparability in the SAR study, chlorine, bromine, and an ethyl group were chosen as side chain substituents on the 4-alkoxyquinolines. In compound **8r**, bromine was positioned at the 6-position of the heterocycle, while chlorine was retained in the side chain, resulting in a MIC of 0.10 μM. This value indicated that molecule **8r** was approximately 2-fold more potent compared to the methoxylated (**8c**) and chlorinated (**8k**) derivatives, which displayed MIC values of 0.22 μM. Interestingly, the dibrominated structure **8s** exhibited a MIC of 0.35 μM, representing nearly a 4.4-fold increase in potency compared to the chlorinated derivative **8l**, with a MIC of 1.55 μM. Once again, the incorporation of an ethyl group attached to the aniline moiety resulted in a 4-alkoxyquinoline capable of inhibiting the *M. tuberculosis* H37Rv strain within the submicromolar range. Compound **8t** exhibited a MIC of 0.05 μM, comparable to its methoxylated (**8i**) and chlorinated (**8n**) counterparts, which displayed MIC values of 0.06 μM and 0.03 μM, respectively. However, the presence of an ethyl group at the 2-position of the heterocyclic ring in molecule **8u** reduced its potency by nearly 2-fold compared to the methyl-substituted structure **8r**. The MIC of 4-alkoxyquinoline **8u** was 0.19 μM, while its methyl-containing counterpart **8r** exhibited a MIC of 0.10 μM. Conversely, the same exchange between methyl and ethyl resulted in a compound with nearly identical activity; both molecules **8v** and **8s** yielded MIC values of 0.34 μM and 0.35 μM, respectively. Finally, structure **8w** exhibited a MIC of 0.10 μM, indicating that the insertion of an ethyl group at the 2-position of the heterocyclic ring diminished the antimycobacterial activity by approximately 2-fold when compared to the MIC value of the methylated derivative **8t**. Altogether, the initial SAR findings appear to suggest a more pronounced correlation between the steric effects and the compound’s activity, rather than the electronic and physicochemical characteristics of the assessed molecules.

It noteworthy that isoniazid and rifampin, which constitute the principal drugs of first-line tuberculosis therapy, showed MIC values of 2.3 μM and 0.2 μM, respectively, against the *M. tuberculosis* H37Rv strain when evaluated under the same experimental conditions. Additionally, the clinical candidate telacebec demonstrated potent inhibition of the bacilli, with an MIC of 0.009 μM.

Employing a MIC threshold of 0.10 μM, seven 4-alkoxyquinolines (**8d**–**e**, **8i**, **8n**, **8r**, **8t**, and **8w**) were selected for assessment against a panel of clinical isolate strains (Table 2). Importantly, the genomes of these multidrug-resistant tuberculosis (MDR-TB) strains have been sequenced, elucidating the genotypic alterations associated with resistance.^21^ Notably, *M. tuberculosis* strains PT2, PT12, and PT20 have been characterized as resistant to a spectrum of drugs, including isoniazid, rifampin, streptomycin, ethionamide, and rifabutin. Furthermore, PT12 and PT20 exhibit resistance to pyrazinamide and ethambutol, with PT12 manifesting additional resistance to amikacin and capreomycin. Interestingly, all evaluated compounds were able to inhibit the growth of MDR-TB strains, displaying MIC values below 0.06 μM. These findings not only underscore the potential effectiveness of the molecules against drug-resistant clinical isolates, but also hint at their possibly superior performance compared to the inhibition of wild-type *M. tuberculosis* H37Rv strain. Furthermore, it is plausible to infer that the synthesized structures exhibit no *in vitro* cross-resistance with key and clinically significant anti-TB drugs. Altogether, these data imply a promising potential for 4-alkoxyquinolines against both drug-susceptible and drug-resistant Mtb strains, likely acting through different molecular targets than those of the classical antimycobacterial drugs.

**Table 2:** *In Vitro* Evaluation of the Selected 4-Alkoxyquinolines against *M. tuberculosis* H37Rv, MDR Strains, and Assessment of HepG2 and Vero Cell Viability

**Entry**	**MIC H37Rv (μM)**	**MIC PT2 (μM)**	**MIC PT12 (μM)**	**MIC PT20 (μM)**	**CC**_**50**_^a^**HepG2 (μM)**	**CC**_**50**_^a^**Vero (μM)**	**SI**^e^**HepG2**	**SI**^e^**Vero**
**8d**	0.10	<0.05	<0.05	<0.05	15.3^b^; 16.8^c^	>20^b^; 12.8^c^	153^b^; 168^c^	>200^b^; 128^c^
**8e**	0.09	<0.05	<0.05	<0.05	10.6^b^; 12.8^c^	8.2^b^; 7.5^c^	118^b^; 142^c^	91^b^; 83^c^
**8i**	0.06	<0.06	<0.06	<0.06	0.3^b^; 8.1^c^	<1^b^; 4.2^c^	5^b^; 135^c^	<17^b^; 70^c^
**8n**	0.03	<0.06	<0.06	<0.06	-d	-d	-	-
**8r**	0.10	<0.05	<0.05	<0.05	>20^b^; > 20^c^	19.8^b^; 11.2^c^	>200^b^; > 200^c^	198^b^; 112^c^
**8t**	0.05	<0.05	<0.05	<0.05	>20^b^; > 20^c^	>20^b^; 11.8^c^	>400^b^; > 400^c^	>400^b^; 236^c^
**8w**	0.10	<0.05	<0.05	<0.05	>20^b^; 8.3^c^	>20^b^; 9.1^c^	>200^b^; 83^c^	>200^b^; 91^c^
**INH**	2.3	291.7	291.7	291.7	-	-	-	-
**RIF**	0.2	>48.6	>48.6	>48.6	-	-	-	-

a The toxicity and selectivity of the compounds were investigated using HepG2 and Vero cell lines. The outcomes were quantified as the concentration causing a 50% reduction in cell viability (CC_50_) using MTT and Neutral Red assays.

b Determined by the MTT method.

c Determined by the Neutral Red method.

d Limited solubility within the applied experimental conditions.

e Selectivity index (SI = CC_50_/MIC H37Rv). INH, Isoniazid. RIF, Rifampin.

Additionally, the selectivity and preliminary toxicity assessment of selected compounds were performed by evaluating the viability of HepG2 and Vero cell lines after exposure to the molecules (Table 2). It is noteworthy that the concentration required to reduce cell viability by 50% (CC_50_) was determined using the MTT (methyl-thiazolyl-tetrazolium)^22^ and neutral red^23^ protocols. The MTT approach probes mitochondrial activity, whereas the neutral red assay provides insights into the lysosomal integrity of the cells. Unfortunately, owing to solubility challenges arising at diminished cosolvent concentrations, was unfeasible to subject molecule **8n** to the mentioned experiments. Among the evaluated structures, 4-alkoxyquinoline **8i** exhibited a notable capacity to inhibit the viability of HepG2 and Vero cells. Notably, apparent cytotoxicity toward mammalian cells was observed in the MTT assay, but this was not evident in the assessment utilizing neutral red. When employing MTT conditions for the assessment of cell viability of **8i**, the selectivity indices (CC_50_/MIC H37Rv) were 5 for HepG2 and less than 17 for Vero cells. Conversely, under neutral red protocol conditions, the selectivity index values were 135 for HepG2 and 70 for Vero cell lines. These results underscore the significance of employing multiple methodologies for evaluating viability in cell assays. The other compounds did not exert substantial alterations in the viability of the investigated cell lines. The presented selectivity indices surpassed 83, exceeding the minimum 10-fold ratio commonly employed in drug discovery campaigns.^24^ It is important to emphasize that among the molecules demonstrating low apparent toxicity to mammalian cells, structure **8t** displayed the highest selectivity indices, with values equal to or exceeding 236 for both evaluated cell lines.

Furthermore, the aqueous solubility of the selected 4-alkoxyquinolines (**8d**–**e**, **8i**, **8n**, **8r**, **8t**, and **8w**) was assessed in solutions simulating stomach (pH 1.2), plasma (pH 7.4), and intestinal (pH 9.1) pH conditions (Table 3). As expected, the compounds exhibited substantially greater solubility at pH 1.2 compared to pH 7.4 and pH 9.1. The selected molecules demonstrated solubility ranging from 200 μM to over 2,230 μM at pH 1.2. Under plasma-like pH conditions, the structures displayed solubility ranging from 0.06 μM to 71.2 μM. Remarkably, these values position certain synthesized 4-alkoxyquinolines with solubility profiles surpassing the threshold of 25 μM, which has been described in early TB drug discovery programs.^25^ Finally, under pH 9.1, the aqueous solubilities of the compounds were obtained with values ranging from 0.37 μM to 61.4 μM. It is important to highlight that molecule **8n** exhibited the lowest aqueous solubility among the evaluated structures, aligning with expectations due to the impracticability of conducting cellular assays for toxicity and selectivity. This 4-alkoxyquinoline demonstrated solubility of merely 0.06 μM and 0.37 μM at pH 7.4 and pH 9.1, respectively. Additionally, despite multiple attempts, compound **8w** could not be quantified through any of the methods tested using UHPLC-DAD.

**Table 3:** Solubility Profiles of Selected 4-Alkoxyquinolines

	**Solubility**^a^		
**Entry**	**pH 1.2**^b^**(μM)**	**pH 7.4**^c^**(μM)**	**pH 9.1**^d^**(μM)**
**8d**	>2,500	71.2	21.03
**8e**	>2,230	2.9	2.49
**8i**	2,650	70.6	61.37
**8n**	200	0.06	0.37
**8r**	>2,470	15.0	57.18
**8t**	>2,500	35.3	30.22
**8w**	-	-	-

a Concentration was determined by UHPLC-DAD after shaking incubation of the compound suspensions at 25 °C for 4 h.

b Using a 0.1 M HCl solution.

c Using PBS.

d Using a 0.1 M NH_4_HCO_3_ solution.

Based on the outcomes from the antimycobacterial assays conducted on both susceptible and resistant Mtb strains, as well as the evaluation of selectivity using two mammalian cell lines and the solubility profile, molecule **8t** was selected for subsequent investigations. The objective was to assess the drug-like attributes of this structure as a representative of its chemical class, thus assessing its potential for integration into future preclinical programs.

Initially, the involvement of the *qcrB* gene product in the antitubercular activity demonstrated by 4-alkoxyquinoline **8t** was examined using a spontaneously generated mutant *M. tuberculosis* strain resistant to 2-(quinolin-4-yloxy)acetamides (Table 4). This resistant strain is known to harbor a mutation in the *qcrB* gene, leading to a T313A amino acid substitution.^16^ The MIC value observed against this resistant strain for the compound **8t** was 3.13 μM, which was approximately 62.5-fold higher than the value exhibited against the *M. tuberculosis* H37Rv strain (MIC = 0.05 μM). These findings suggest that the β-subunit of the cytochrome *bc*_1_ oxidase complex, a component of the respiratory electron transport chain, plays a central role in the antimycobacterial activity of this molecule. Therefore, in line with our previous findings^13^ and those of other research groups,^26^ it appears that molecular simplification and chain extension preserves the antitubercular action mechanism exhibited by 2-(quinolin-4-yloxy)acetamides. Moreover, structure **8t** underwent evaluation against *Staphylococcus aureus* ATCC 25923, *Escherichia coli* ATCC 25922, as well as the multidrug-resistant clinical isolates *Acinetobacter baumannii* and *Klebsiella pneumoniae* (Table 4). At a concentration of 20 μM, the 4-alkoxyquinoline was not able to inhibit bacterial growth, suggesting a potential selectivity of this compound toward Mtb inhibition.

**Table 4:** *In Vitro* Activities of the Selected 4-Alkoxyquinoline **8t** against a 2-(Quinolin-4-yloxy)acetamide-Resistant Strain, Gram-Positive and Gram-Negative Bacterial Strains, and Assessment of Its Chemical Stability, Permeability, and Metabolic Stability

**Entry**	**Properties**
**8t**	MIC _*qcrB*-T313A_^a^ = 3.13 μM
	MIC _*S. aureus*_ = > 20 μM
	MIC _*E. coli*_ = > 20 μM
	MIC _*A. baumannii*_ = > 20 μM
	MIC _*K. pneumoniae*_ = > 20 μM
	Chemical Stability^b^ pH 1.2^c^ = 100%
	Chemical Stability^b^ pH 7.4^d^ = 5.3%
	Chemical Stability^b^ pH 9.1^e^ = 6.0%
	PAMPA = 0.2 × 10^−6^ cm/s
	Cl_int_^f^ = 58 mL/min/kg
	*t*_1/2_^g^ = 7.0 min

a 2-(Quinolin-4-yloxy)acetamide-resistant spontaneous mutant containing a unique alteration in the *qcrB* gene (ACC to GCC at nucleotide position 937 or a T313A amino substitution).

b Percentage of remaining compound after incubation at 37 °C for 24 h.

c 0.1 M HCl solution.

d Using PBS.

e Using a 0.1 M NH_4_HCO_3_ solution.

f Intrinsic clearance of rat liver microsomes.

g Half-life.

Furthermore, some *in vitro* ADME properties were examined for molecule **8t** (Table 4). It is noteworthy that the stability of structure **8t** remained unaltered after 24 h of incubation at pH 1.2. Conversely, when subjected to neutral (pH 7.4) or alkaline (pH 9.1) conditions, the compound’s concentration was significantly reduced. This data suggests that utilizing hydrochloride as a counterion could serve as an alternative to stabilize the 4-alkoxyquinoline in aqueous solutions. In terms of passive permeability, compound **8t** exhibited low permeability, as evaluated by parallel artificial membrane permeability assay (PAMPA). Specifically, molecule **8t** exhibited a permeability of 0.2 × 10^–6^ cm/s, whereas alprenolol, employed as a positive control, exhibited a permeability of 4.1 × 10^–6^ cm/s under the applied experimental conditions. Lastly, the metabolic stability of structure **8t** was assessed in the presence of rat liver microsomes. The 4-alkoxyquinoline exhibited a high metabolic rate with clearance of 58 mL/min/kg which resulted in a half-life of 7 min. Notably, these values closely resembled those exhibited by verapamil, utilized as a positive control in the experiment. Verapamil displayed a clearance of 56 mL/min/kg along with a half-life of 7.6 min under the applied experimental conditions.

Based on the results describing antimycobacterial activity against susceptible and drug-resistant strains, selectivity using eukaryotic cells, preliminary mechanism of action data, and *in vitro* ADME profile, 4-alkoxyquinoline **8t** was evaluated in a murine macrophage model of TB infection using the first-line drug isoniazid as positive control (Table 5). The objective was to determine whether the physicochemical characteristics of the molecule allow it to pass through different cellular compartments and reduce the bacterial load within macrophages. Compared to the early control group, macrophages in the untreated group showed an increase of approximately 1.9 log_10_ colony-forming units (log_10_ CFU) over 5 days, indicating intracellular growth of Mtb. Treatment with 1 μM and 5 μM of the compound **8t** prevented bacterial growth and maintained stable bacterial loads inside the macrophages. The groups treated with 1 μM and 5 μM reduced the Mtb load by 1.27 log_10_ CFU and 1.07 log_10_ CFU, respectively, compared to the untreated group. However, when compared to the early control group, the number of colonies was not reduced, indicating a bacteriostatic effect of 4-alkoxyquinoline **8t** on the intracellular growth of the *M. tuberculosis* H37Rv strain. It is important to note the lack of a dose–response effect regarding the intracellular activity of the structure, as there was no significant difference in bacteriostatic effects between the groups of macrophages treated with 1 μM and 5 μM of the compound **8t**. As expected, isoniazid effectively reduced macrophage infection compared to both the early control and untreated control groups. At a concentration of 1 μM, the drug lowered the bacterial load to 2.25 log_10_ CFU, demonstrating its bactericidal effect under the given test conditions.

**Table 5:** Intracellular Activity of 4-Alkoxyquinoline **8t** in Murine Macrophages Model of TB Infection^a^

Entry	Log_10_ CFU/well (Mean ± SD)
Early Control	3.28 ± 0.11
Untreated	5.17 ± 0.40^b^
**8t** (1 μM)	3.90 ± 0.30^d^
**8t** (5 μM)	4.10 ± 0.02^d^
**INH** (1 μM)	2.25 ± 0.15^c^^,^^d^

a SD, standard deviation.

b *P* < 0.0001 compared to early control group.

c *P* < 0.002 compared to early control group.

d *P* < 0.0001 compared to untreated group which was dosed with 0.5% DMSO. Multiple comparisons were performed with Tukey’s test. INH, isoniazid.

Considering the potential challenges in terms of pharmacokinetic exposure occasioned by its low permeability and high metabolic rate observed *in vitro*, an experiment was conducted to determine whether compound **8t** could be effectively absorbed and reach systemic circulation in effective amounts after administration via gavage to mice (Table 6). Employing a concentration of 300 mg/kg, the presence of molecule **8t** in plasma was quantified, revealing concentrations exceeding 7.49 μM for a span of up to 8 h. Notably, the peak plasma concentration (*C*_max_) obtained for the structure **8t** was 32.1 ± 3.9 μM, achieved 1 h after administration (*T*_max_). The observed half-life (*t*_1/2_) was 4.3 ± 1.2 h, and the exposure to the 4-alkoxyquinoline **8t** from time 0 to 8 h was 127.5 ± 5.7 μM*h. Furthermore, the elimination rate constant (K_e_) indicated that the concentration of compound **8t** decreased at a rate of 0.16 (16%) over the evaluated time interval. It is noteworthy that incomplete drug elimination may influence the values reported for *t*_1/2_, area under the curve (AUC), and K_e_. Lastly, after an 8 h period after oral administration, the measured molecule concentration was 9.80 μM. Notably, this concentration stands 196-fold higher than the MIC exhibited by the compound against the *M. tuberculosis* H37Rv strain. These preliminary absorption findings underscore that structure **8t** is probably capable of absorption, allowing it to penetrate the systemic circulation of the animals and maintain substantial concentrations for at least 8 h, significantly surpassing the levels required to inhibit the growth of the bacillus.

**Table 6:** Absorption Profile and Preliminary Pharmacokinetic Parameters of 4-Alkoxyquinoline **8t** after Oral Gavage Administration to Mice

	**Concentration**^a^	**Pharmacokinetics**^b^	
**Time (h)**	**μM ± SD**	**Parameters**	**Mean ± SD**
**0.25**	7.49 ± 2.72	*C*_max_ (μM)	32.1 **±**3.9
**0.5**	11.10 ± 1.24	*T*_max_ (h)	1
**0.75**	15.73 ± 1.61	*t*_1/2_ (h)	4.3 **±** 1.2
**1**	32.15 ± 3.94	AUC_0-t_ (μM*h)	127.5 ± 5.7
**4**	13.45 ± 2.01	K_e_ (h^–1^)	0.16
**8**	9.80 ± 2.13		

a Plasma concentration was determined using UHPLC-DAD after a single dose (300 mg/kg) of **8t**, the results were presented as the mean of two animals over time, accompanied by the standard deviation.

b Noncompartmental pharmacokinetics analysis. *C*_max_, peak concentration. *T*_max_, time to peak plasma concentration. *t*_1/2_, elimination half-life. AUC_0-t_, area under the concentration–time curve from 0 to time t. K_e_, elimination rate constant.

## 3 Conclusion

In this study, we present the design and synthesis of a novel series of 4-alkoxyquinolines and demonstrate their *in vitro* antimycobacterial activities. The synthetic procedures were executed using readily available reagents and reactants within well established and straightforward protocols. Additionally, the compounds exhibited potent activity against both drug-sensitive and MDR-TB strains. Interestingly, the lead molecule manifests its antitubercular activity by targeting the cytochrome *bc*_1_ complex, thereby expanding the potential utility of this chemical class in addressing nonreplicating forms of Mtb. Furthermore, the design strategy, involving molecular simplification and chain extension, yielded a compound characterized by favorable kinetic solubility and chemical stability using acidic conditions. This molecule also exhibited low permeability and a high metabolism rate in the presence of rat microsomes, a metabolism profile akin to that of verapamil. Additionally, the leading structure demonstrated bacteriostatic activity in a murine macrophages model of TB infection. Lastly, a preliminary absorption assessment of the lead compound revealed, for the first time, a simplified structure derived from 2-(quinolin-4-yloxy)acetamides that exhibited promising *in vivo* exposure following oral administration to mice. This suggests the potential of this chemical class to yield novel anti-TB drug candidates. Ongoing efforts are focused on further structural modifications to achieve a more comprehensive SAR, and these results will be presented in due course.

## 4 Experimental Section

### 4.1 Synthesis and Structure: Apparatus and Analysis

All solvents and reagents used in this study were obtained from commercial suppliers and were employed in the experiments without any additional purification steps. The progress of the reactions was monitored by thin-layer chromatography (TLC). Melting points were determined using a Microquímica MQAPF-302 apparatus. ^1^H and ^13^C NMR spectra were acquired on an Avance III HD Bruker spectrometer located at the Pontifical Catholic University of Rio Grande do Sul. Chemical shifts (δ) were reported in parts per million (ppm) with reference to DMSO-*d*_*6*_ or CDCl_3_, which served as the solvents, and to tetramethylsilane (TMS), used as an internal standard. NMR spectra were processed using MestReNova v.14.0.0–23239 (2019 Mestrelab Research S.L.). In the ^1^H NMR spectra, the following abbreviations were used to denote splitting patterns: s for Singlet, d for Doublet, t for Triplet, q for Quartet, p for Pentet (or quintet), m for Multiplet, and dd for Doublet of doublets. High-resolution mass spectra (HRMS) were acquired using either an LTQ Orbitrap Discovery (Thermo Fisher Scientific, Bremen, Germany) or a MicroTof-QII (Bruker Corporation, Bremen, Germany) mass spectrometer, both equipped with an electrospray ionization source (ESI) operating in positive ionization mode. The purity of the compounds was assessed using a Dionex Ultimate 3000 UHPLC chromatograph (Dionex Corporation, Germering, Germany). This UHPLC system was equipped with a binary pump, an automatic injector, and a diode array (DAD) detector. Data acquisition and processing were performed using Chromeleon 6.80 SR11 (Build 3160) software. The analytical conditions included an RP column (5 μm Nucleodur C-18, 250 × 4.6 mm), a flow rate of 1.5 mL/min, and UV detection at 254–260 nm. The elution gradient consisted of 100% water (0.1% acetic acid) from 0 to 7 min, followed by a linear gradient from 100% water (0.1% acetic acid) to 90% acetonitrile/methanol (1:1, v/v) from 7 to 15 min (15–30 min). Subsequently, the system returned to 100% water (0.1% acetic acid) within 5 min (30–35 min) and was maintained for an additional 10 min (35–45 min). All evaluated compounds exhibited a purity of >95%.

### 4.2 *General Procedure for the Synthesis of 4-Bromoalkoxyquinolines***7a**–***e***

The compounds **7a**–**e** were synthesized from 4-hydroxyquinolines **6a**–**e**. The reaction was carried out in a round-bottom flask containing the respective 4-hydroxyquinoline (2 mmol), 1,3-dibromopropane (1.21 g, 6 mmol), cesium carbonate (1.95 g, 6 mmol), sodium iodide (0.075 g, 0.5 mmol) in acetonitrile (50 mL). The reaction mixture was stirred at room temperature (25 °C) for 24 h. Subsequently, extraction was performed using chloroform (3 × 50 mL) and saturated ammonium chloride solution. The organic layer was dried over magnesium sulfate and then concentrated under reduced pressure. The purification of 4-bromoalkoxyquinolines **7a**–**e** was accomplished by chromatography using silica gel as the stationary phase and a mobile phase consisting of either 100% ethyl acetate or a mixture of ethyl acetate and hexane in a 3:7 ratio.

### 4.3 *General Procedure for the Synthesis 4-Alkoxyquinolines***8a**–***w***

The compounds **8a**–**w** were synthesized from alkylation reaction involving appropriately substituted anilines. The reactions were conducted in a round-bottom flasks containing the 4-bromoalkoxyquinoline of interest (1 mmol), the respective aniline (2 mmol), potassium carbonate (0.41 g, 3 mmol), sodium iodide (0.15 g, 1 mmol), and toluene (20 mL). The reaction mixture was heated at 110 °C for 48–72 h. Afterward, extraction was performed using chloroform (3 × 50 mL) and saturated ammonium chloride solution. The organic layer was dried over magnesium sulfate and then concentrated under reduced pressure. The purification of the products was conducted by a recrystallization process using ethyl acetate, or by employing chromatographic separation with silica gel as the stationary phase and a mobile phase comprising mixtures of hexane and ethyl acetate in ratios of 3:7 or 1:1, or alternatively using a mixture of chloroform and methanol in ratios of 99:1, 98:2, or 97:3.

### 4.4 *N*-(3-((6-methoxy-2-methylquinolin-4-yl)oxy)propyl)aniline (**8a**)

column chromatography on silica gel (chloroform: methanol, 98:2), pinkish solid, yield: 0.148 g (46%); mp.: 165–167 °C; UHPLC: 96% (*t*_R_ = 13.54 min); ^1^H NMR (400 MHz, DMSO-*d*_6_) δ ppm 2.23 (p, *J* = 6.3 Hz, 3H), 2.84 (s, 3H), 3.32 (t, *J* = 6.6 Hz, 2H), 3.95 (s, 3H), 4.62 (t, *J* = 6.2 Hz, 2H), 6.56 (t, *J* = 7.3 Hz, 1H), 6.66 (d, *J* = 7.9 Hz, 2H), 7.05–7.12 (m, 2H), 7.51 (d, *J* = 3.2 Hz, 2H), 7.71 (dd, *J* = 9.3, 2.8 Hz, 1H), 8.09 (d, *J* = 9.3 Hz, 1H); ^13^C NMR (101 MHz, DMSO-*d*_6_) δ ppm 20.25, 27.52, 55.94, 69.00, 101.06, 103.71, 112.36 (2C), 116.09, 120.15, 121.47, 125.65, 128.87 (2C), 133.81, 148.26, 156.04, 158.25, 165.49; HRMS (ESI) *m*/*z*: 323.1748 [M + H]^+^; calcd. for C_20_H_23_N_2_O_2_: 323.1754.

### 4.5 4-Fluoro-*N*-(3-((6-methoxy-2-methylquinolin-4-yl)oxy)propyl)aniline (**8b**)

column chromatography on silica gel (chloroform: methanol, 97:3), white solid, yield: 0.129 g (38%); mp.: 185–187 °C; UHPLC: 96% (*t*_R_ = 13.82 min); ^1^H NMR (400 MHz, DMSO-*d*_6_) δ ppm 2.22 (p, *J* = 6.4 Hz, 2H), 2.83 (s, 3H), 3.28 (t, *J* = 6.6 Hz, 2H), 3.95 (s, 3H), 4.61 (t, *J* = 6.3 Hz, 2H), 6.60–6.66 (m, 2H), 6.88–6.95 (m, 2H), 7.47–7.53 (m, 2H), 7.69 (dd, *J* = 9.2, 2.8 Hz, 1H), 8.08 (d, *J* = 9.2 Hz, 1H); ^13^C NMR (101 MHz, DMSO-*d*_6_) δ ppm 20.40, 27.54, 55.91, 68.92, 101.01, 103.65, 112.84, 112.92, 115.09 (2C), 115.31 (2C), 120.12, 121.72, 125.47, 134.14, 145.25, 156.06, 158.16, 165.28; HRMS (ESI) *m*/*z*: 341.1651 [M + H]^+^; calcd. for C_20_H_22_FN_2_O_2_: 341.1660.

### 4.6 4-Chloro-*N*-(3-((6-methoxy-2-methylquinolin-4-yl)oxy)propyl)aniline (**8c**)

column chromatography on silica gel (chloroform: methanol, 98:2), yellowish solid, yield: 0.289 g (81%); mp.: 193–196 °C; UHPLC: 97% (*t*_R_ = 14.44 min); ^1^H NMR (400 MHz, DMSO-*d*_6_) δ ppm 2.21 (p, *J* = 6.4 Hz, 2H), 2.84 (s, 3H), 3.29 (t, *J* = 6.5 Hz, 2H), 3.95 (s, 3H), 4.61 (t, *J* = 6.2 Hz, 2H), 6.59–6.66 (m, 2H), 7.04–7.10 (m, 2H), 7.47–7.54 (m, 2H), 7.70 (dd, *J* = 9.3, 2.8 Hz, 1H), 8.08 (d, *J* = 9.3 Hz, 1H); ^13^C NMR (101 MHz, DMSO-*d*_6_) δ ppm 20.32, 27.46, 55.93, 68.92, 101.07, 103.69, 113.32 (2C), 118.83, 120.14, 121.56, 125.59, 128.50 (2C), 133.93, 147.59, 156.04, 158.22, 165.41. HRMS (ESI) *m*/*z*: 357.1381 [M + H]^+^; calcd. for C_22_H_22_ClN_2_O_2_: 357.1364.

### 4.7 4-Bromo-*N*-(3-((6-methoxy-2-methylquinolin-4-yl)oxy)propyl)aniline (**8d**)

recrystallized from ethyl acetate, white solid, yield: 0.177 g (44%); mp.: 200–204 °C; UHPLC: 96% (*t*_R_ = 14.59 min); ^1^H NMR (400 MHz, DMSO-*d*_6_) δ ppm 2.20 (p, *J* = 6.3 Hz, 2H), 2.82 (s, 3H), 3.28 (t, *J* = 6.6 Hz, 2H), 3.95 (s, 3H), 4.60 (t, *J* = 6.2 Hz, 2H), 6.55–6.59 (m, 2H), 7.14–7.19 (m, 2H), 7.47 (s, 1H), 7.53 (d, *J* = 2.8 Hz, 1H), 7.70 (dd, *J* = 9.2, 2.8 Hz, 1H), 8.08 (d, *J* = 9.3 Hz, 1H); ^13^C NMR (101 MHz, DMSO-*d*_6_) δ ppm 20.30, 27.44, 39.21, 55.87, 68.83, 101.18, 103.58, 106.14, 113.86 (2C), 120.16, 121.59, 125.47, 131.23 (2C), 133.98, 147.88, 156.02, 158.21, 165.40; HRMS (ESI) *m*/*z*: 401.0860 [M + H]^+^; calcd. for C_20_H_22_BrN_2_O_2_: 401.0859.

### 4.8 4-Iodo-*N*-(3-((6-methoxy-2-methylquinolin-4-yl)oxy)propyl)aniline (**8e**)

recrystallized from ethyl acetate, yellowish solid, yield: 0.211 (47%); mp.: 198–199 °C; UHPLC: 96% (*t*_R_ = 14.87 min); ^1^H NMR (400 MHz, DMSO-*d*_6_) δ ppm 2.19 (p, *J* = 6.4 Hz, 2H), 2.80 (s, 3H), 3.28 (t, *J* = 6.6 Hz, 2H), 3.95 (s, 3H), 4.57 (t, *J* = 6.2 Hz, 2H), 6.45–6.49 (m, 2H), 7.30–7.33 (m, 2H), 7.45 (s, 1H), 7.51 (d, *J* = 2.8 Hz, 1H), 7.69 (dd, *J* = 9.3, 2.8 Hz, 1H), 8.03 (d, *J* = 9.2 Hz, 1H); ^13^C NMR (101 MHz, DMSO-*d*_6_) δ ppm 20.74, 27.47, 55.90, 68.66, 75.95, 101.02, 103.55, 114.63 (2C), 120.15, 122.21, 125.36, 134.68, 137.08 (2C), 148.38, 156.16, 158.11, 165.08; HRMS (ESI) *m*/*z*: 449.0734 [M + H]^+^; calcd. for C_22_H_22_IN_2_O_2_: 449.0721.

### 4.9 *N*-(3-((6-methoxy-2-methylquinolin-4-yl)oxy)propyl)-4-(trifluoromethyl)aniline (**8f**)

recrystallized from ethyl acetate, yellow solid, yield: 0.098 g (25%); mp.: 199–203 °C; UHPLC: 96% (*t*_R_ = 14.69 min); ^1^H NMR (400 MHz, DMSO-*d*_6_) δ ppm 2.24 (p, *J* = 6.4 Hz, 2H), 2.83 (s, 3H), 3.38 (t, *J* = 6.6 Hz, 3H), 3.95 (s, 3H), 4.62 (t, *J* = 6.2 Hz, 2H), 6.72 (d, *J* = 8.5 Hz, 2H), 7.35 (d, *J* = 8.4 Hz, 2H), 7.51–7.55 (m, 2H), 7.72 (dd, *J* = 9.2, 2.8 Hz, 1H), 8.07 (d, *J* = 9.3 Hz, 1H); ^13^C NMR (101 MHz, DMSO-*d*_6_) δ ppm 20.31, 27.37, 55.95, 68.86, 101.18, 103.72, 111.27, 114.98, 115.29, 120.20, 121.53, 123.97, 125.74, 126.16, 126.20, 126.65, 133.84, 151.63, 156.10, 158.31, 165.56; HRMS (ESI) *m*/*z*: 391.1643 [M + H]^+^; calcd. for C_21_H_22_F_3_N_2_O_2_: 391.1628.

### 4.10 *N*-(3-((6-methoxy-2-methylquinolin-4-yl)oxy)propyl)-4-(trifluoromethoxy)aniline (**8g**)

recrystallized from ethyl acetate, white solid, yield: 0.065 g (16%); mp.: 181–188 °C; UHPLC: 97% (*t*_R_ = 14.89 min); ^1^H NMR (400 MHz, DMSO-*d*_6_) δ ppm 2.14 (p, *J* = 6.5 Hz, 2H), 2.54 (s, 2H), 3.28 (q, *J* = 6.5 Hz, 2H), 3.86 (s, 3H), 4.33 (t, *J* = 6.2 Hz, 2H), 6.04 (t, *J* = 5.6 Hz, 1H), 6.60–6.67 (m, 2H), 6.89 (s, 1H), 7.05 (d, *J* = 8.5 Hz, 2H), 7.32 (dd, *J* = 9.1, 2.9 Hz, 1H), 7.38 (d, *J* = 2.9 Hz, 1H), 7.76 (d, *J* = 9.1 Hz, 1H); ^13^C NMR (101 MHz, DMSO-*d*_6_) δ ppm 25.49, 28.38, 55.78, 66.46, 100.23, 102.44, 112.69 (2C), 120.34, 121.77, 122.55 (2C), 130.02, 138.75, 138.77, 144.52, 148.57, 156.70, 157.57, 160.32; HRMS (ESI) *m*/*z*: 407.1570 [M + H]^+^; calcd. for C_21_H_22_F_3_N_2_O_3_: 407.1577.

### 4.11 *N*-(3-((6-methoxy-2-methylquinolin-4-yl)oxy)propyl)-4-methylaniline (**8h**)

column chromatography on silica gel (chloroform: methanol, 98:2), brownwish solid, yield: 0.101 g (30%); mp.: 171–175 °C; UHPLC: 96% (*t*_R_ = 13.47 min); ^1^H NMR (400 MHz, DMSO-*d*_6_) δ ppm 2.24 (s, 3H), 2.87 (s, 3H), 3.40 (p, 2H), 3.56 (t, *J* = 11.0 Hz, 3H), 3.96 (s, 3H), 4.62 (t, *J* = 5.8 Hz, 2H), 7.13 (d, *J* = 8.8 Hz, 4H), 7.46–7.54 (m, 2H), 7.66–7.73 (m, 1H), 8.34 (d, *J* = 8.7 Hz, 1H); ^13^C NMR (101 MHz, DMSO-*d*_6_) δ ppm 20.38, 20.84, 21.01, 26.56, 56.62, 68.95, 101.63, 104.25, 120.67, 121.92, 123.28, 126.00, 130.22 (2C), 130.39 (2C), 134.39, 137.30, 156.29, 158.73, 165.62; HRMS (ESI) *m*/*z*: 337.1897 [M + H]^+^; calcd. for C_21_H_25_N_2_O_2_: 337.1911.

### 4.12 4-Ethyl-*N*-(3-((6-methoxy-2-methylquinolin-4-yl)oxy)propyl)aniline (**8i**)

recrystallized from ethyl acetate, yellowish solid, yield: 0.088 g (25%); mp.: 174–178 °C; UHPLC: 95% (*t*_R_ = 14.19 min); ^1^H NMR (400 MHz, DMSO-*d*_6_) δ ppm 1.10 (t, *J* = 7.6 Hz, 3H), 2.21 (p, *J* = 6.3 Hz, 2H), 2.44 (q, *J* = 7.5 Hz, 2H), 2.80 (s, 3H), 3.29 (t, *J* = 6.6 Hz, 2H), 3.95 (s, 3H), 4.60 (t, *J* = 6.2 Hz, 2H), 6.54–6.61 (m, 2H), 6.88–6.95 (m, 2H), 7.47 (s, 1H), 7.53 (t, *J* = 2.8 Hz, 1H), 7.70 (ddd, *J* = 9.3, 2.9, 1.2 Hz, 1H), 8.02 (d, *J* = 9.2 Hz, 1H); ^13^C NMR (101 MHz, DMSO-*d*_6_) δ ppm 15.99, 20.58, 27.20, 27.57, 39.68, 55.88, 68.82, 101.00, 103.56, 112.45 (2C), 120.11, 121.93, 125.46, 128.08 (2C), 131.28, 134.31, 146.20, 156.09, 158.13, 165.25; HRMS (ESI) *m*/*z*: 351.2053 [M + H]^+^; calcd. for C_22_H_27_N_2_O_2_: 351.2067.

### 4.13 4-Isopropyl-*N*-(3-((6-methoxy-2-methylquinolin-4-yl)oxy)propyl)aniline (**8j**)

recrystallized from ethyl acetate, yellowish solid, yield: 0.193 g (53%); mp.: 166–170 °C; UHPLC: 97% (*t*_R_ = 15.19 min); ^1^H NMR (400 MHz, DMSO-*d*_6_) δ ppm 1.13 (d, *J* = 6.9 Hz, 7H), 2.22 (p, *J* = 6.4 Hz, 2H), 2.71 (h, *J* = 6.9 Hz, 1H), 2.82 (s, 3H), 3.30 (t, *J* = 6.6 Hz, 2H), 3.95 (s, 3H), 4.61 (t, *J* = 6.2 Hz, 2H), 6.57–6.65 (m, 2H), 6.93–7.00 (m, 2H), 7.46–7.54 (m, 2H), 7.70 (dd, *J* = 9.2, 2.8 Hz, 1H), 8.03 (d, *J* = 9.2 Hz, 1H); ^13^C NMR (101 MHz, DMSO-*d*_6_) δ ppm 20.46, 24.13 (2C), 27.51, 32.39, 40.09, 55.91, 68.89, 101.05, 103.61, 112.62 (2C), 120.12, 121.72, 125.54, 126.58 (2C), 134.06, 136.34, 145.98, 156.06, 158.17, 165.36; HRMS (ESI) *m*/*z*: 365.2228 [M + H]^+^; calcd. for C_23_H_29_N_2_O_2_: 365.2224.

### 4.14 4-Chloro-*N*-(3-((6-chloro-2-methylquinolin-4-yl)oxy)propyl)aniline (**8k**)

column chromatography on silica gel (hexane: ethyl acetate, 70:30, then 50:50), brown solid, yield: 0.166 g (46%); mp.: 118–121 °C; UHPLC: 96% (*t*_R_ = 16.21 min); ^1^H NMR (400 MHz, DMSO-*d*_6_) δ ppm 2.13 (p, *J* = 6.1 Hz, 2H), 2.59 (d, *J* = 1.5 Hz, 3H), 3.25 (q, *J* = 6.3 Hz, 2H), 4.34 (t, *J* = 6.1 Hz, 2H), 6.03 (s, 1H), 6.57–6.66 (m, 2H), 7.00 (d, *J* = 1.8 Hz, 1H), 7.08 (dd, *J* = 8.7, 1.5 Hz, 2H), 7.69 (dd, *J* = 9.0, 2.4 Hz, 1H), 7.86 (dd, *J* = 8.9, 1.7 Hz, 1H), 8.04 (d, *J* = 2.6 Hz, 1H); ^13^C NMR (101 MHz, DMSO-*d*_6_) δ ppm 25.27, 27.74, 66.46, 102.68, 113.28 (2C), 118.71, 120.08, 120.27, 128.54 (2C), 129.27, 130.07 (2C), 146.57, 147.72, 159.92, 160.74; HRMS (ESI) *m*/*z*: 361.0881 [M + H]^+^; calcd. for C_19_H_19_Cl_2_N_2_O: 361.0869.

### 4.15 4-Bromo-*N*-(3-((6-chloro-2-methylquinolin-4-yl)oxy)propyl)aniline (**8l**)

column chromatography on silica gel (chloroform: methanol, 99:1, then 97:3), yellow solid, yield: 0.166 g (41%); mp.: 135–139 °C; UHPLC: 97% (*t*_R_ = 17.36 min); ^1^H NMR (400 MHz, CDCl_3_) δ ppm 2.24 (p, *J* = 6.3 Hz, 2H), 2.69 (s, 4H), 3.41 (t, *J* = 6.6 Hz, 2H), 4.32 (t, *J* = 5.9 Hz, 2H), 6.49–6.54 (m, 2H), 6.63 (s, 1H), 7.22–7.26 (m, 3H), 7.60 (ddd, *J* = 9.0, 2.4, 0.7 Hz, 1H), 7.97 (d, *J* = 9.0 Hz, 1H), 8.08 (d, *J* = 2.4 Hz, 1H); ^13^C NMR (101 MHz, CDCl_3_) δ ppm 25.29, 28.86, 41.02, 66.82, 102.13, 109.43, 114.49 (2C), 120.63, 120.84, 129.13, 131.14, 131.33, 132.16 (2C), 146.29, 147.04, 160.32, 161.27; HRMS (ESI) *m*/*z*: 405.0379 [M + H]^+^; calcd. for C_19_H_19_BrClN_2_O: 405.0364.

### 4.16 *N*-(3-((6-Chloro-2-methylquinolin-4-yl)oxy)propyl)-4-(trifluoromethoxy)aniline (**8m**)

column chromatography on silica gel (chloroform: methanol, 99:1), white solid, yield: 0.082g (20%); mp.: 122–126 °C; UHPLC: 98% (*t*_R_ = 16.85 min); ^1^H NMR (400 MHz, CDCl_3_) δ ppm 2.25 (p, *J* = 6.3 Hz, 2H), 2.66 (s, 3H), 3.43 (t, *J* = 6.6 Hz, 2H), 4.28 (t, *J* = 5.9 Hz, 2H), 6.59–6.62 (m, 3H), 7.01–7.07 (m, 2H), 7.59 (dd, *J* = 9.0, 2.4 Hz, 1H), 7.89 (d, *J* = 9.0 Hz, 1H), 8.06 (d, *J* = 2.4 Hz, 1H); ^13^C NMR (101 MHz, CDCl_3_) δ ppm 25.70, 28.66, 41.00, 66.36, 101.87, 112.97 (2C), 119.46, 120.46, 120.69, 120.72, 121.99, 122.55 (2C), 129.64, 130.73, 130.83, 140.56, 140.58, 146.75, 146.90, 160.45, 160.63; HRMS (ESI) *m*/*z*: 411.1092 [M + H]^+^; calcd. for C_20_H_19_ClF_3_N_2_O_2_: 411.1082.

### 4.17 *N*-(3-((6-Chloro-2-methylquinolin-4-yl)oxy)propyl)-4-ethylaniline (**8n**)

column chromatography on silica gel (hexane: ethyl acetate, 50:50), dark brown solid, yield: 0.124 g (35%); mp.: 113–118 °C; UHPLC: 98% (*t*_R_ = 21.58 min); ^1^H NMR (400 MHz, CDCl_3_) δ ppm 1.19 (t, *J* = 7.6 Hz, 3H), 2.24 (p, *J* = 6.3 Hz, 2H), 2.54 (q, *J* = 7.6 Hz, 2H), 2.65 (s, 3H), 3.43 (t, *J* = 6.6 Hz, 2H), 4.28 (t, *J* = 6.0 Hz, 2H), 6.57–6.64 (m, 3H), 6.99–7.06 (m, 2H), 7.58 (dd, *J* = 9.0, 2.4 Hz, 1H), 7.87 (dd, *J* = 8.9, 0.5 Hz, 1H), 8.07–8.12 (m, 1H); ^13^C NMR (101 MHz, CDCl_3_) δ ppm 15.96, 25.83, 27.92, 28.91, 41.13, 66.44, 101.85, 112.99 (2C), 120.55, 120.77, 128.70 (2C), 129.78, 130.59, 130.68, 133.59, 145.91, 147.14, 160.47, 160.63; HRMS (ESI) *m*/*z*: 355.1580 [M + H]^+^; calcd. for C_21_H_24_ClN_2_O: 355.1572.

### 4.18 4-Chloro-*N*-(3-((6-chloro-2-ethylquinolin-4-yl)oxy)propyl)aniline (**8o**)

column chromatography on silica gel (hexane: ethyl acetate, 70:30, then 50:50), brown solid, yield: 0.049 g (13%); mp.: 124–129 °C; UHPLC: 98% (*t*_R_ = 16.80 min); ^1^H NMR (400 MHz, DMSO-*d*_6_) δ ppm 1.30 (t, *J* = 7.6 Hz, 3H), 2.13 (p, *J* = 6.5 Hz, 2H), 2.86 (q, *J* = 7.6 Hz, 2H), 3.26 (q, *J* = 6.3 Hz, 2H), 4.35 (t, *J* = 6.1 Hz, 2H), 5.97 (t, *J* = 5.6 Hz, 1H), 6.57–6.65 (m, 2H), 6.98 (s, 1H), 7.06–7.12 (m, 2H), 7.69 (dd, *J* = 9.0, 2.5 Hz, 1H), 7.88 (d, *J* = 9.1 Hz, 1H), 8.06 (d, *J* = 2.4 Hz, 1H); ^13^C NMR (101 MHz, DMSO-*d*_6_) δ ppm 13.38, 27.75, 31.78, 66.36, 101.62, 113.25 (2C), 118.75, 120.25, 120.28, 128.54 (2C), 129.28, 129.97, 130.30, 146.63, 147.66, 160.01, 165.47; HRMS (ESI) *m*/*z*: 375.1036 [M + H]^+^; calcd. for C_20_H_21_Cl_2_N_2_O: 375.1025.

### 4.19 4-Bromo-*N*-(3-((6-chloro-2-ethylquinolin-4-yl)oxy)propyl)aniline (**8p**)

column chromatography on silica gel (hexane: ethyl acetate, 70:30), black solid, yield: 0.067 g (16%); mp.: 115–119 °C; UHPLC: 96% (*t*_R_ = 18.20 min); ^1^H NMR (400 MHz, CDCl_3_) δ ppm 1.37 (t, *J* = 7.6 Hz, 4H), 2.25 (p, *J* = 6.3 Hz, 2H), 2.91 (q, *J* = 7.6 Hz, 2H), 3.42 (t, *J* = 6.6 Hz, 2H), 4.30 (t, *J* = 5.8 Hz, 2H), 6.51–6.55 (m, 2H), 6.63 (s, 1H), 7.24–7.28 (m, 3H), 7.72 (dd, *J* = 8.9, 2.3 Hz, 1H), 7.84 (d, *J* = 8.9 Hz, 1H), 8.26 (d, *J* = 2.3 Hz, 1H); ^13^C NMR (101 MHz, CDCl_3_) δ pmm 13.89, 28.66, 32.78, 40.97, 66.38, 100.67, 109.20, 114.35 (2C), 118.73, 121.20, 123.95, 130.14, 132.07 (2C), 133.16, 146.93, 147.34, 160.62, 165.74; HRMS (ESI) *m*/*z*: 419.0533 [M + H]^+^; calcd. for C_20_H_21_BrClN_2_O: 419.0520.

### 4.20 *N*-(3-((6-chloro-2-ethylquinolin-4-yl)oxy)propyl)-4-ethylaniline (**8q**)

column chromatography on silica gel (hexane: ethyl acetate, 70:30), brown solid, yield: 0.122 g (33%); mp.: 76–82 °C; UHPLC: 95% (*t*_R_ = 22.36 min); ^1^H NMR (400 MHz, CDCl_3_) δ ppm 1.19 (t, *J* = 7.6 Hz, 3H), 1.36 (t, *J* = 7.6 Hz, 3H), 2.24 (p, *J* = 6.3 Hz, 2H), 2.54 (q, *J* = 7.6 Hz, 2H), 2.91 (q, *J* = 7.6 Hz, 2H), 3.43 (t, *J* = 6.6 Hz, 2H), 4.29 (t, *J* = 5.9 Hz, 2H), 6.59–6.64 (m, 3H), 7.01–7.05 (m, 2H), 7.58 (dd, *J* = 9.0, 2.4 Hz, 1H), 7.90 (dd, *J* = 9.0, 0.5 Hz, 1H), 8.11 (d, *J* = 2.3 Hz, 1H); ^13^C NMR (101 MHz, CDCl_3_) δ ppm 13.95, 15.96, 27.92, 28.92, 32.78, 41.16, 66.41, 100.65, 112.99 (2C), 120.75 (2C), 128.69 (2C), 129.97, 130.51, 130.65, 133.58, 145.91, 147.18, 160.80, 165.60; HRMS (ESI) *m*/*z*: 369.1733 [M + H]^+^; calcd. for C_22_H_26_ClN_2_O: 369.1728.

### 4.21 *N*-(3-((6-bromo-2-methylquinolin-4-yl)oxy)propyl)-4-chloroaniline (**8r**)

column chromatography on silica gel (hexane: ethyl acetate, 70:30, then 50:50), brown solid, yield: 0.089 g (22%); mp.: 120–127 °C; UHPLC: 96% (*t*_R_ = 16.60 min); ^1^H NMR (400 MHz, DMSO-*d*_6_) δ ppm 2.13 (p, *J* = 6.4 Hz, 2H), 2.60 (s, 3H), 3.26 (t, *J* = 6.6 Hz, 2H), 4.34 (t, *J* = 6.1 Hz, 2H), 6.61 (d, *J* = 8.6 Hz, 2H), 7.00 (s, 1H), 7.09 (d, *J* = 8.7 Hz, 2H), 7.75–7.86 (m, 2H), 8.22 (d, *J* = 2.1 Hz, 1H); ^13^C NMR (101 MHz, DMSO-*d*_6_) δ ppm 25.01, 27.71, 66.59, 102.68, 113.25 (2C), 117.79, 118.75, 120.55, 123.54, 128.53 (2C), 129.69, 132.84, 146.18, 147.64, 160.13, 160.72; HRMS (ESI) *m*/*z*: 405.0373 [M + H]^+^; calcd. for C_19_H_19_BrClN_2_O: 405.0364.

### 4.22 4-Bromo-*N*-(3-((6-bromo-2-methylquinolin-4-yl)oxy)propyl)aniline (**8s**)

column chromatography on silica gel (hexane: ethyl acetate, 70:30), black solid, yield: 0.094 g (21%); mp.: 134–138 °C; UHPLC: 97% (*t*_R_ = 17.97 min); ^1^H NMR (400 MHz, CDCl_3_) δ ppm 2.25 (p, *J* = 6.4 Hz, 3H), 2.67 (s, 3H), 3.42 (t, *J* = 6.6 Hz, 2H), 4.29 (t, *J* = 5.8 Hz, 2H), 6.49–6.56 (m, 2H), 6.62 (s, 1H), 7.22–7.29 (m, 2H), 7.73 (dd, *J* = 9.0, 2.2 Hz, 1H), 7.85 (d, *J* = 9.0 Hz, 1H), 8.25 (d, *J* = 2.2 Hz, 1H); ^13^C NMR (101 MHz, CDCl_3_) δ ppm 25.65, 28.64, 40.91, 66.54, 101.95, 109.24, 114.36 (2C), 118.88, 118.95, 120.98, 124.02, 129.61, 132.08 (2C), 133.39, 146.91, 146.93, 160.57; HRMS (ESI) *m*/*z*: 448.9860 [M + H]^+^; calcd. for C_19_H_18_Br_2_N_2_O: 448.9859.

### 4.23 *N*-(3-((6-bromo-2-methylquinolin-4-yl)oxy)propyl)-4-ethylaniline (**8t**)

recrystallized from ethyl acetate, yellow solid, yield: 0.124 g (31%); mp.: 156–164 °C; UHPLC: 98% (*t*_R_ = 17.84 min); ^1^H NMR (400 MHz, DMSO-*d*_6_) δ ppm 1.11 (t, *J* = 7.6 Hz, 3H), 2.22 (p, *J* = 6.4 Hz, 2H), 2.47 (q, *J* = 7.6 Hz, 2H), 2.81 (s, 3H), 3.34 (t, *J* = 6.7 Hz, 2H), 4.57 (t, *J* = 6.0 Hz, 2H), 6.72 (d, *J* = 8.0 Hz, 2H), 7.00 (d, *J* = 8.0 Hz, 2H), 7.50 (s, 1H), 8.02 (d, *J* = 9.0 Hz, 1H), 8.15 (dd, *J* = 8.9, 2.2 Hz, 1H), 8.42 (t, *J* = 2.3 Hz, 1H); ^13^C NMR (101 MHz, DMSO-*d*_6_) δ ppm 15.95, 21.53, 27.12, 27.32, 40.98, 68.92, 104.27 (2C), 114.04, 120.23, 120.40, 123.35, 124.73, 128.29 (2C), 133.32, 136.30, 138.87, 144.30, 159.86, 164.82; HRMS (ESI) *m*/*z*: 399.1076 [M + H]^+^; calcd. for C_21_H_24_BrN_2_O: 399.1067.

### 4.24 *N*-(3-((6-bromo-2-ethylquinolin-4-yl)oxy)propyl)-4-chloroaniline (**8u**)

column chromatography on silica gel (hexane: ethyl acetate, 70:30, then 50:50), brownwish solid, yield: 0.126 g (30%); mp.: 146–148 °C; UHPLC: 96% (*t*_R_ = 17.07 min); ^1^H NMR (400 MHz, CDCl_3_) δ ppm 1.32–1.41 (m, 4H), 2.25 (p, *J* = 6.4 Hz, 2H), 2.94 (q, *J* = 7.6 Hz, 3H), 3.41 (t, *J* = 6.6 Hz, 2H), 4.31 (t, *J* = 5.9 Hz, 2H), 6.57 (dd, *J* = 8.4, 1.5 Hz, 2H), 6.64 (s, 1H), 7.12 (dd, *J* = 8.8, 1.7 Hz, 2H), 7.72 (dd, *J* = 9.0, 2.3 Hz, 1H), 7.90 (d, *J* = 9.0 Hz, 1H), 8.25 (s, 1H); ^13^C NMR (101 MHz, CDCl_3_) δ ppm 13.89, 28.62, 32.36, 40.94, 66.63, 100.76, 113.83 (2C), 119.00, 121.13, 122.11, 124.03, 129.16 (2C), 129.45, 133.46, 146.52, 161.07, 165.60; HRMS (ESI) *m*/*z*: 419.0533 [M + H]^+^; calcd. for C_21_H_24_BrN_2_O: 419.0520.

### 4.25 4-Bromo-*N*-(3-((6-bromo-2-ethylquinolin-4-yl)oxy)propyl)aniline (**8v**)

column chromatography on silica gel (hexane: ethyl acetate, 70:30), greenwish solid, yield: 0.083 g (18%); mp.: 145–147 °C; UHPLC: 98% (*t*_R_ = 17.98 min); ^1^H NMR (400 MHz, CDCl_3_) δ ppm 1.37 (t, *J* = 7.5 Hz, 3H), 2.25 (p, *J* = 6.4 Hz, 2H), 2.92 (q, *J* = 7.6 Hz, 2H), 3.42 (t, *J* = 6.6 Hz, 2H), 4.30 (t, *J* = 5.9 Hz, 2H), 6.49–6.56 (m, 2H), 6.63 (s, 1H), 7.23–7.28 (m, 2H), 7.59 (dd, *J* = 9.0, 2.3 Hz, 1H), 7.91 (d, *J* = 9.0 Hz, 1H), 8.08 (d, *J* = 2.3 Hz, 1H); ^13^C NMR (101 MHz, CDCl_3_) δ ppm 13.92, 28.67, 32.72, 40.94, 66.33, 100.66, 109.19, 114.34 (2C), 120.66, 120.68, 129.96, 130.61, 130.77, 132.07 (2C), 146.93, 147.07, 160.75, 165.56. HRMS (ESI) *m*/*z*: 463.0022 [M + H]^+^; calcd. for C_20_H_21_Br_2_N_2_O: 463.0015.

### 4.26 *N*-(3-((6-bromo-2-ethylquinolin-4-yl)oxy)propyl)-4-ethylaniline (**8w**)

column chromatography on silica gel (hexane: ethyl acetate, 70:30), brownwish solid, yield: 0.112 g (27%); mp.: 92–95 °C; UHPLC: 96% (*t*_R_ = 22.26 min); ^1^H NMR (400 MHz, CDCl_3_) δ ppm 1.19 (t, *J* = 7.6 Hz, 3H), 1.36 (t, *J* = 7.6 Hz, 3H), 2.24 (p, *J* = 6.3 Hz, 2H), 2.54 (q, *J* = 7.6 Hz, 2H), 2.90 (q, *J* = 7.7 Hz, 2H), 3.43 (t, *J* = 6.6 Hz, 2H), 4.29 (t, *J* = 5.9 Hz, 2H), 6.61 (d, *J* = 8.6 Hz, 3H), 7.00–7.05 (m, 2H), 7.71 (dd, *J* = 9.0, 2.3 Hz, 1H), 7.83 (d, *J* = 8.9 Hz, 1H), 8.28 (d, *J* = 2.3 Hz, 1H); ^13^C NMR (101 MHz, CDCl_3_) δ ppm 13.90, 15.96, 27.92, 28.89, 32.80, 41.16, 66.46, 100.65, 112.98 (2C), 118.63, 121.25, 124.04, 128.69 (2C), 130.11, 133.07, 133.57, 145.90, 147.37, 160.69, 165.74; HRMS (ESI) *m*/*z*: 413.1230 [M + H]^+^; calcd. for C_22_H_26_BrN_2_O: 413.1223.

### 4.27 *Mycobacterium tuberculosis* Inhibition Assay

The procedure for determining the minimum inhibitory concentration (MIC) in Mtb aims to determine whether the molecule can inhibit bacterial growth under *in vitro* conditions. The dilutions were made in round-bottom microplates (96 wells). The solution of isoniazid (INH), rifampicin (RIF), and telacebec (Q203) (positive control), and the synthesized compounds were diluted in 100% dimethyl sulfoxide (DMSO) at a concentration of 2 mg/mL. For the assay, aliquots were diluted in Middlebrook 7H9 medium containing 10% ADC (albumin, dextrose, catalase) and 5% DMSO in a series of assays of varying concentrations, depending on the solubility of each compound, finishing at the maximum possible concentration. To determine the aforesaid solubility limit, the solutions were evaluated for the possible presence of crystals. If crystals were identified, the aliquot would be diluted once more with half of the previous concentration. Growth controls without antibiotics and sterility controls without inoculation were included in the procedures. Mycobacterial strains were cultured in Middlebrook 7H9 containing 10% OADC (oleic acid, albumin, dextrose, catalase) and 0.05% tween 80. Cells were vortexed with sterile glass beads (4 mm) for 5 min to disrupt clamps and then left to rest for 20 min. The supernatants were measured in a spectrophotometer at an absorbance of 600 nm. Mtb suspensions were aliquoted and stored at −20 °C. Each suspension was properly diluted in Middlebrook 7H9 broth containing 10% ADC to reach an optical density of 0.006 at 600 nm. Ultimately, 100 μL of this solution was added to each well of the plate, except for the sterility control one. The plates were covered, sealed, and incubated at 37 °C. After 7 days of incubation, 60 μL of 0.01% resazurin solution was added to each well and the plate was incubated for an additional 48 h at 37 °C. The MIC was defined by the lowest concentration of the molecule that did not change the color of the medium from blue to pink, which would have meant the reduction of resazurin and bacterial growth. Assays were performed three times for each structure on different dates and two concordant results or the highest MIC value between assays was showed in micromolar concentration (μM). The laboratory reference strains were: Mtb H37Rv (ATCC 27294) and three strains considered multidrug-resistant (MDR) isolates of Mtb named PT2, PT12, and PT20, which were obtained from patients in the Lisbon Health Region, Lisbon, Portugal. Finally, INH and RIF were used as control drugs in all experiments.

### 4.28 Cellular Viability Evaluation

The evaluation of Vero and HepG2 cell viability was conducted using MTT (3-[4,5-dimethylthiazol-2-yl]-2,5 diphenyl tetrazolium bromide) and neutral red uptake (NRU) assays. Both cell lines were grown in DMEM medium (Dulbecco’s Modified Eagle Medium, Gibco, Grand Island, NY, USA) supplemented with 10% inactivated fetal bovine serum from Invitrogen, 1% antibiotic (penicillin and streptomycin) from Gibco, and 0.1% fungizone from Gibco. Cells were seeded in a 96-well culture plate (4 × 10^3^ HepG2 and 2 × 10^3^ Vero) and incubated overnight at 37 °C to allow for cell attachment. The media was then removed and replaced with fresh media containing each compound at a final concentration of 1 μM, 5 μM, or 20 μM using 1% DMSO. Cells were incubated during 72 h at 37 °C under a 5% CO_2_ atmosphere. After the incubation, medium was removed and replaced with MTT reagent (5 mg/mL), followed by additional 4 h incubation. Then, 100 μL of DMSO was added to dissolve the formazan crystals and the absorbance was measured at 570 nm (EZ Read 400 microplate reader, Biochrom, Cambridge, UK). Precipitated formazan crystals were used as a direct measure of living cells with active mitochondrial metabolism. For the NRU assay, after 72 h of cell incubation with the treatments, the cells were washed with PBS and 200 μL of neutral red dye solution (25 μg/mL, Sigma) prepared in serum-free medium was added to each well. The plate was then incubated for 3 h at 37 °C under 5% CO_2_. Afterward, cells were washed with PBS, and 100 μL of a desorb solution (ethanol/acetic acid/water, 50:1:49) was added to each well and shaken gently for 30 min to extract the neutral red dye from viable cells. The absorbance was measured at 562 nm (EZ Read 400 microplate reader (Biochrom, Holliston, MA, USA)). The percentage of cell viability was calculated using the vehicle control wells (1% DMSO) as the maximum cell viability. The results were presented as concentration required to reduce cell viability by 50% (CC_50_) using three independent experiments performed in triplicate.

### 4.29 Solubility

The solubility assay employed 1 mL of buffer solution (PBS 1X, pH 7.4, or 0.1 M HCl, pH 1.0, or NH_4_HCO_3_ 0.1 M, pH 9.1). Each sample was weighed at 1 mg. Subsequently, the final solutions were vortexed, and the remaining suspensions were agitated for 4 h at 25 °C. Following this, the solutions were centrifuged at 13,000 rpm for 20 min at 25 °C, resulting in the formation of a pellet. The analysis was conducted via ultra high-performance liquid chromatography (UHPLC-DAD) utilizing single-point calibration with a known concentration of the compounds in DMSO.

### 4.30 Evaluation of Possible Broad-spectrum Activity

Four nonmycobacterial species were used: the standard strains *Staphylococcus aureus* ATCC 25923 and *Escherichia coli* ATCC 25922, along with the clinical isolates *Klebsiella pneumoniae* and *Acinetobacter baumannii*, both exhibiting extensive drug resistance. The isolates were stored at −20 °C in a medium with 20% glycerol. To prepare the cultures, 100 μL of each frozen stock was inoculated in Brain Heart Infusion (BHI) broth and incubated for 24 h at 37 °C. Cultures were then streaked on BHI agar plates and incubated for an additional 24 h to obtain isolated colonies. The inoculum was prepared by suspending isolated colonies in 0.85% saline to achieve a turbidity of 0.5 on the McFarland scale (1.5 × 10^8^ CFU/mL). Compound **8t** was diluted in DMSO and Mueller-Hinton (MH) broth to create a stock solution with a concentration of 40 μM and a final DMSO concentration of 4%. In a 96-well U-bottom polystyrene plate, 100 μL of MH broth were added to three wells as negative controls, while 100 μL of MH broth plus 1 μL of adjusted inoculum served as positive controls. For the test wells, 100 μL of MH broth, 100 μL of the diluted compound, and 1 μL of inoculum were combined to reach a final concentration of 20 μM of the compound and 2% DMSO. The plates were incubated at 37 °C for 24 h, and bacterial growth inhibition was assessed visually.

### 4.31 Chemical Stability Evaluation

The assessment of chemical stability at different pH levels provides insights into the quantity or concentration of the test compound that remains unchanged and, consequently, available for absorption. In this study, 4-alkoxyquinoline **8t** (10 μM) was incubated at 37 °C for 24 h in the presence of a buffer solution at controlled pH levels: 1.2 (simulating the stomach pH with 0.1 M HCl), 7.4 (simulating plasma pH with PBS), and 9.1 (simulating intestinal pH with 0.1 M NH_4_HCO_3_). Following this incubation period, the test compounds were quantified using HPLC-MS/MS, with alprenolol serving as the analytical control. This experiment was conducted by CEMSA (São Paulo, SP, Brazil).

### 4.32 Permeability Assay

The Parallel Artificial Membrane Permeability Assay (PAMPA) utilizes an artificial membrane as an efficient and rapid model for studying and evaluating the passive permeation potential of drug candidates. In this study, 4-alkoxyquinoline **8t** was quantified (HPLC-MS/MS) after incubation in two solutions separated by an artificial and porous lipid membrane. The results of this test were expressed in terms of diffusion rate (permeation). Initially, compound **8t** at a concentration of 10 μM (in 2% DMSO) was added to the donor aqueous phase (pH 7.4). After 5 h at 25 °C, an aliquot of the receptor solution (pH 7.4), through which the compound was theoretically transported via passive diffusion, was withdrawn, and the concentration of the tested molecule was measured. Based on the concentration results, the permeation velocity value was determined, with alprenolol serving as the analytical control. Permeability is typically categorized as low (<1.0 × 10^–6^ cm/s) or high (>1.0 × 10^–6^ cm/s).^27^ This experiment was conducted by CEMSA (São Paulo, SP, Brazil).

### 4.33 Metabolic Stability Assay

In brief, 4-alkoxyquinoline **8t** at a concentration of 2 μM were subjected to incubation with a rat microsomes preparation containing NADPH at 37 °C. The consumption of the compound from the incubation mixture was monitored at 0, 5, 15, and 30 min using the HPLC-MS/MS technique to determine the *in vitro* disappearance half-life. Verapamil was employed as the positive control. Intrinsic clearance is typically categorized as low (<16 mL/min/kg), moderate (16–47 mL/min/kg), and high (>47 mL/min/kg).^27^ This experiment was conducted by CEMSA (São Paulo, SP, Brazil).

### 4.34 Intracellular Activity in a Murine Macrophages Model of TB Infection

The experiment utilized murine macrophage RAW 264.7 cells cultured in RPMI 1640 medium (Gibco) supplemented with 10% fetal bovine serum (FBS), without antimicrobials or fungizone. Cells were seeded at a density of 5 × 10^3^ cells per well in a 24-well flat-bottom plate and incubated at 37 °C with 5% CO_2_. After the incubation period, the medium was removed, and the cells were washed with preheated sterile PBS to remove nonadherent cells. *M. tuberculosis* strain H37Rv (ATCC 27294) was grown in 10 mL of Middlebrook 7H9 medium (Becton-Dickinson-BD) supplemented with 10% BD Difco BBL Middlebrook OADC enrichment (oleic acid, albumin, dextrose, catalase), 0.05% (v/v) Tween 80 (Sigma-Aldrich), and 0.2% (v/v) glycerol (Sigma-Aldrich) to mid log phase (OD600 ≅ 0.8). The culture was then diluted in RPMI medium with 10% FBS (without antimicrobials or fungizone) to obtain 1.5 × 10^4^ CFUs/well and 2.5 × 10^4^ CFUs/well of Mtb for MOI 3:1 and 5:1, respectively, in two independent experiments. The cells infection was caried out after adding Mtb to the plates, they were incubated for 3 h at 37 °C with 5% CO_2_. The infected cells were then washed twice with sterile PBS to remove any remaining Mtb in the medium. Early control (EC) cells were lysed at the beginning of treatment with 1 mL of 0.025% SDS diluted in 0.9% saline. Serial dilutions were performed in 0.9% saline, and the lysed cells were plated on Middlebrook 7H10 (Becton-Dickinson-BD) supplemented with 10% OADC and 0.5% glycerol. Infected cells were treated with 1 μM or 5 μM of **8t** in triplicate. A 4 mM solution of each compound was prepared in DMSO and diluted in RPMI medium to a final concentration of 1 μM or 5 μM, with a final DMSO concentration of 0.5%. The late control (LC) group was treated with 0.5% DMSO in RPMI medium. After 5 days of treatment, cells were washed twice with sterile PBS and lysed with 1 mL of 0.025% SDS diluted in 0.9% saline. The cell lysate was diluted in 0.9% saline and plated on 7H10 medium supplemented with 10% OADC and 0.5% glycerol. After an incubation period of 3 to 4 weeks at 37 °C, CFUs were counted, establishing a limit of detection (LOD) between 20 and 300 CFU per plate. Calculated CFU values were converted to log_10_ CFU, and the results of the two independent experiments were combined. Finally, the results were expressed as the mean log_10_ CFU per well ± standard deviation (mean log_10_ CFU/well ± SD). The groups were compared by one-way analysis of variance (ANOVA), followed by Tukey’s post-test, using GraphPad Prism 9.0 (GraphPad, San Diego, CA, USA).

### 4.35 In Vivo Absorption Profiling

Male CF1 mice (4–5 weeks old) were used for the absorption profiling evaluation (*n* = 12). All animals were sourced from the Center of Experimental Biological Models at Pontifícia Universidade Católica do Rio Grande do Sul (CeMBE/PUCRS). The mice were housed under controlled conditions, with humidity levels of 40–60% and room temperature maintained at 24 ± 2 °C, following a 12 h light/dark cycle. Food and water were provided ad libitum. The study protocol was approved by the Animal Ethics Committee at Pontifícia Universidade Católica do Rio Grande do Sul (CEUA/PUCRS, Porto Alegre, RS, Brazil; protocol number 10649). The experiment also adhered to the Brazilian guidelines for the production, maintenance, and use of animals in teaching or scientific research, as established by the National Council for the Control of Animal Experimentation (CONCEA, Brazil).

The determination of the absorption profile of compound **8t** was conducted by using gavage administration. A single dose of 300 mg/kg (≈ 750 μmol/kg) per animal was administered. The suspension was prepared in a saline solution using ultrasound for 1 h. Following administration, the animals were euthanized, and blood samples were collected at the specified time intervals: 0.25, 0.5, 0.75, 1, 4, and 8 h. Two animals were employed for each experimental time point, yielding independent duplicate results. Blood collected from each mouse underwent centrifugation for 30 min at 4 °C and 13000 rpm to separate the plasma. Subsequently, 100 μL of plasma was extracted per sample and mixed with 100 μL of acetonitrile, followed by vortexing for 1 min. The resulting mixture was then centrifuged for 15 min at 4 °C and 13000 rpm. After this process, the supernatant was separated and combined with 300 μL of dichloromethane in a vial. Once again, the resulting mixture was vortexed for 10 s. After phase separation, the organic phase was transferred to another vial, and the dichloromethane was evaporated under reduced pressure. Finally, the sample was reconstituted with 150 μL of a 1% acetic acid solution for the subsequent quantification of the compound. The quantification method was carried out using UHPLC-DAD Shimadzu LC-2060 3D equipment, and detection was performed at a wavelength of 254 nm. The analytical calibration curve in plasma encompassed six concentrations of the analyte, ranging from 1.25 ug/mL to 50 ug/mL of the compound. The R^2^ value of the analytical curve was determined to be 0.9969.

## Data Availability

Data will be made available on request.

## Supporting Information Available

^1^H NMR and ^13^C NMR spectra of synthesized compounds (**8a**–**w**), UHPLC chromatogram for *in vivo* evaluated compound **8t** (PDF)
Molecular formula strings and assay data (CSV)

## Abbreviations

ADC: Albumin dextrose catalase
ADME: Absorption, distribution, metabolism, and excretion
AUC_0-t_: Area under the concentration–time curve from 0 to time *t*
CFU: Colony-forming unit
DMSO: Dimethyl sulfoxide
FBS: Fetal bovine serum
HPLC-MS/MS: High-performance liquid chromatography with tandem mass spectrometry
INH: Isoniazid
*K*_e_: Elimination rate constant
MIC: Minimum inhibitory concentration
NRU: Neutral red uptake
PAMPA: parallel artificial membrane permeability assay
PBS: Phosphate-buffered saline
Q203: Telacebec
RIF: Rifampicin
RPMI: Roswell Park Memorial Institute medium
SAR: Structure–activity relationship
*t*_1/2_: Half-life
TLC: Thin-layer chromatography
UHPLC-DAD: Ultra high-performance liquid chromatography with diode array detection

## References

[ref1] World Health Organization Global tuberculosis report 2023. https://www.who.int/teams/global-tuberculosis-programme/tb-reports/global-tuberculosis-report-2023. (Accessed 20 November 2023).

[ref2] WHO. WHO operational handbook on tuberculosis. Module 4: Treatment - Drug-Resistant Tuberculosis Treatment, 2022 update; World Health Organization: Geneva, 2022. https://www.who.int/publications/i/item/9789240065116. 36630546

[ref3] Diacon A. H.; Pym A.; Grobusch M.; Patientia R.; Rustomjee R.; Page-Shipp L.; Pistorius C.; Krause R.; Bogoshi M.; Churchyard G.; Venter A.; Allen J.; Palomino J. C.; De Marez T.; van Heeswijk R. P.; Lounis N.; Meyvisch P.; Verbeeck J.; Parys W.; de Beule K.; Andries K.; Mc Neeley D. F. The diarylquinoline TMC207 for multidrug-resistant tuberculosis. N. Engl. J. Med. 2009, 360 (23), 2397–2405. 10.1056/NEJMoa0808427. 19494215

[ref4] Gler M. T.; Skripconoka V.; Sanchez-Garavito E.; Xiao H.; Cabrera-Rivero J. L.; Vargas-Vasquez D. E.; Gao M.; Awad M.; Park S. K.; Shim T. S.; Suh G. Y.; Danilovits M.; Ogata H.; Kurve A.; Chang J.; Suzuki K.; Tupasi T.; Koh W. J.; Seaworth B.; Geiter L. J.; Wells C. D. Delamanid for multidrug-resistant pulmonary tuberculosis. New England journal of medicine 2012, 366 (23), 2151–2160. 10.1056/NEJMoa1112433. 22670901

[ref5] Conradie F.; Diacon A. H.; Ngubane N.; Howell P.; Everitt D.; Crook A. M.; Mendel C. M.; Egizi E.; Moreira J.; Timm J.; McHugh T. D.; Wills G. H.; Bateson A.; Hunt R.; Van Niekerk C.; Li M.; Olugbosi M.; Spigelman M.; Treatment of Highly Drug-Resistant Pulmonary Tuberculosis. N. Engl. J. Med. 2020, 382 (10), 893–902. 10.1056/NEJMoa1901814. 32130813 PMC6955640

[ref6] Yoshiyama T.; Takaki A.; Aono A.; Mitarai S.; Okumura M.; Ohta K.; Kato S. Multidrug Resistant Tuberculosis With Simultaneously Acquired Drug Resistance to Bedaquiline and Delamanid. Clinical infectious diseases: an official publication of the Infectious Diseases Society of America 2021, 73 (12), 2329–2331. 10.1093/cid/ciaa1064. 32730621

[ref7] Millard J.; Rimmer S.; Nimmo C.; O’Donnell M. Therapeutic Failure and Acquired Bedaquiline and Delamanid Resistance in Treatment of Drug-Resistant TB. Emerging infectious diseases 2023, 29 (5), 1081–1084. 10.3201/eid2905.221716. 37081529 PMC10124645

[ref8] Pissinate K.; Villela A. D.; Rodrigues-Junior V.; Giacobbo B. C.; Grams E. S.; Abbadi B. L.; Trindade R. V.; Roesler Nery L.; Bonan C. D.; Back D. F.; Campos M. M.; Basso L. A.; Santos D. S.; Machado P. 2-(Quinolin-4-yloxy)acetamides Are Active against Drug-Susceptible and Drug-Resistant Mycobacterium tuberculosis Strains. ACS medicinal chemistry letters 2016, 7 (3), 235–239. 10.1021/acsmedchemlett.5b00324. 26985307 PMC4789679

[ref9] Giacobbo B. C.; Pissinate K.; Rodrigues-Junior V.; Villela A. D.; Grams E. S.; Abbadi B. L.; Subtil F. T.; Sperotto N.; Trindade R. V.; Back D. F.; Campos M. M.; Basso L. A.; Machado P.; Santos D. S. New insights into the SAR and drug combination synergy of 2-(quinolin-4-yloxy)acetamides against Mycobacterium tuberculosis. European journal of medicinal chemistry 2017, 126, 491–501. 10.1016/j.ejmech.2016.11.048. 27914363

[ref10] Borsoi A. F.; Alice L. M.; Sperotto N.; Ramos A. S.; Abbadi B. L.; Macchi Hopf F. S.; Silva Dadda A. d.; Rambo R. S.; Madeira Silva R. B.; Paz J. D.; Pissinate K.; Muniz M. N.; Neves C. E.; Galina L.; González L. C.; Perelló M. A.; de Matos Czeczot A.; Leyser M.; de Oliveira S. D.; de Araújo Lock G.; de Araújo B. V.; Costa T. D.; Bizarro C. V.; Basso L. A.; Machado P. Antitubercular Activity of Novel 2-(Quinoline-4-yloxy)acetamides with Improved Drug-Like Properties. ACS Med. Chem. Lett. 2022, 13 (8), 1337–1344. 10.1021/acsmedchemlett.2c00254. 35978694 PMC9376999

[ref11] Borsoi A. F.; Silva Ramos A.; Sperotto N.; Abbadi B. L.; Souza Macchi Hopf F.; da Silva Dadda A.; Scheibler Rambo R.; Neves Muniz M.; Delgado Paz J.; Silveira Grams E.; Fries da Silva F.; Pissinate K.; Galina L.; Calle González L.; Silva Duarte L.; Alberton Perelló M.; de Matos Czeczot A.; Bizarro C. V.; Basso L. A.; Machado P. Exploring Scaffold Hopping for Novel 2-(Quinolin-4-yloxy)acetamides with Enhanced Antimycobacterial Activity. ACS medicinal chemistry letters 2024, 15 (4), 493–500. 10.1021/acsmedchemlett.3c00570. 38628799 PMC11017393

[ref12] Macchi F. S.; Pissinate K.; Villela A. D.; Abbadi B. L.; Rodrigues-Junior V.; Nabinger D. D.; Altenhofen S.; Sperotto N.; da Silva Dadda A.; Subtil F. T.; de Freitas T. F.; Erhart Rauber A. P.; Borsoi A. F.; Bonan C. D.; Bizarro C. V.; Basso L. A.; Santos D. S.; Machado P. 1H-Benzo[d]imidazoles and 3,4-dihydroquinazolin-4-ones: Design, synthesis and antitubercular activity. European journal of medicinal chemistry 2018, 155, 153–164. 10.1016/j.ejmech.2018.06.005. 29885576

[ref13] Borsoi A. F.; Paz J. D.; Abbadi B. L.; Macchi F. S.; Sperotto N.; Pissinate K.; Rambo R. S.; Ramos A. S.; Machado D.; Viveiros M.; Bizarro C. V.; Basso L. A.; Machado P. Design, synthesis, and evaluation of new 2-(quinoline-4-yloxy)acetamide-based antituberculosis agents. European journal of medicinal chemistry 2020, 192, 112179 10.1016/j.ejmech.2020.112179. 32113048

[ref14] Jardim Etchart R.; Rambo R. S.; Lopes Abbadi B.; Sperotto N.; Ev Neves C.; Fries Silva F.; Dornelles M.; Duarte L.; Souza Macchi F.; Alberton Perelló M.; Vescia Lourega R.; Valim Bizarro C.; Basso L. A.; Machado P. Synthesis and Antimycobacterial Activity of 3-Phenyl-1H-indoles. Molecules 2021, 26 (17), 5148 10.3390/molecules26175148. 34500579 PMC8433792

[ref15] Paz J. D.; de Moura Sperotto N. D.; Ramos A. S.; Pissinate K.; da Silva Rodrigues Junior V.; Abbadi B. L.; Borsoi A. F.; Rambo R. S.; Minotto A. C. C.; da Silva Dadda A.; Galina L.; Hopf F. S. M.; Muniz M. N.; Martinelli L. K. B.; Roth C. D.; Silva R. B. M.; Perelló M. A.; de Matos Czeczot A.; Neves C. E.; Duarte L. S.; Leyser M.; de Oliveira S. D.; Bizarro C. V.; Machado P.; Basso L. A. Novel 4-aminoquinolines: Synthesis, inhibition of the Mycobacterium tuberculosis enoyl-acyl carrier protein reductase, antitubercular activity, SAR, and preclinical evaluation. Eur. J. Med. Chem. 2023, 245 (Pt 1), 114908 10.1016/j.ejmech.2022.114908. 36435016

[ref16] Subtil F. T.; Villela A. D.; Abbadi B. L.; Rodrigues-Junior V. S.; Bizarro C. V.; Timmers L. F. S. M.; de Souza O. N.; Pissinate K.; Machado P.; López-Gavín A.; Tudó G.; González-Martín J.; Basso L. A.; Santos D. S. Activity of 2-(quinolin-4-yloxy)acetamides in Mycobacterium tuberculosis clinical isolates and identification of their molecular target by whole-genome sequencing. International journal of antimicrobial agents 2018, 51 (3), 378–384. 10.1016/j.ijantimicag.2017.08.023. 28843821

[ref17] Ballell L.; Bates R. H.; Young R. J.; Alvarez-Gomez D.; Alvarez-Ruiz E.; Barroso V.; Blanco D.; Crespo B.; Escribano J.; González R.; Lozano S.; Huss S.; Santos-Villarejo A.; Martín–Plaza J. J.; Mendoza A.; Rebollo-Lopez M. J.; Remuiñan–Blanco M.; Lavandera J. L.; Pérez–Herran E.; Gamo-Benito F. J.; García–Bustos J. F.; Barros D.; Castro J. P.; Cammack N. Fueling open-source drug discovery: 177 small-molecule leads against tuberculosis. ChemMedChem 2013, 8 (2), 313–321. 10.1002/cmdc.201200428. 23307663 PMC3743164

[ref18] Pitta E.; Rogacki M. K.; Balabon O.; Huss S.; Cunningham F.; Lopez-Roman E. M.; Joossens J.; Augustyns K.; Ballell L.; Bates R. H.; Van der Veken P. Searching for New Leads for Tuberculosis: Design, Synthesis, and Biological Evaluation of Novel 2-Quinolin-4-yloxyacetamides. Journal of medicinal chemistry 2016, 59 (14), 6709–6728. 10.1021/acs.jmedchem.6b00245. 27348630

[ref19] Phummarin N.; Boshoff H. I.; Tsang P. S.; Dalton J.; Wiles S.; Barry C. E. 3rd; Copp B. R. SAR and identification of 2-(quinolin-4-yloxy)acetamides as Mycobacterium tuberculosis cytochrome bc1 inhibitors. MedChemComm 2016, 7 (11), 2122–2127. 10.1039/C6MD00236F. 28337336 PMC5292992

[ref20] Palomino J. C.; Martin A.; Camacho M.; Guerra H.; Swings J.; Portaels F. Resazurin microtiter assay plate: simple and inexpensive method for detection of drug resistance in Mycobacterium tuberculosis. Antimicrob. Agents Chemother. 2002, 46 (8), 2720–2722. 10.1128/AAC.46.8.2720-2722.2002. 12121966 PMC127336

[ref21] Perdigão J.; Silva H.; Machado D.; Macedo R.; Maltez F.; Silva C.; Jordao L.; Couto I.; Mallard K.; Coll F.; Hill-Cawthorne G. A.; McNerney R.; Pain A.; Clark T. G.; Viveiros M.; Portugal I. Unraveling Mycobacterium tuberculosis genomic diversity and evolution in Lisbon, Portugal, a highly drug resistant setting. BMC genomics 2014, 15 (1), 991 10.1186/1471-2164-15-991. 25407810 PMC4289236

[ref22] van Meerloo J.; Kaspers G. J.; Cloos J. Cell sensitivity assays: the MTT assay. Methods in molecular biology (Clifton, N.J.) 2011, 731, 237–245. 10.1007/978-1-61779-080-5_20. 21516412

[ref23] Repetto G.; del Peso A.; Zurita J. L. Neutral red uptake assay for the estimation of cell viability/cytotoxicity. Nature protocols 2008, 3 (7), 1125–1131. 10.1038/nprot.2008.75. 18600217

[ref24] Indrayanto G.; Putra G. S.; Suhud F. Validation of in-vitro bioassay methods: Application in herbal drug research. Profiles of drug substances, excipients, and related methodology 2021, 46, 273–307. 10.1016/bs.podrm.2020.07.005. 33461699

[ref25] Singh V.; Chibale K. Strategies to Combat Multi-Drug Resistance in Tuberculosis. Accounts of chemical research 2021, 54 (10), 2361–2376. 10.1021/acs.accounts.0c00878. 33886255 PMC8154215

[ref26] Murnane R.; Zloh M.; Tanna S.; Allen R.; Santana-Gomez F.; Parish T.; Brucoli F. Synthesis and antitubercular activity of novel 4-arylalkyl substituted thio-, oxy- and sulfoxy-quinoline analogues targeting the cytochrome bc1 complex. Bioorganic chemistry 2023, 138, 106659 10.1016/j.bioorg.2023.106659. 37336104

[ref27] Di L.; Kerns E. H.; Drug-Like Properties: Concepts, Structure Design and Methods; Academic Press: Cambridge, MA, 2008.

