# Supplementary material for: Unveiling the Antimycobacterial Potential of Novel 4-Alkoxyquinolines: Insights into Selectivity, Mechanism of Action, and In Vivo Exposure
Source: J Med Chem. 2024 Dec 4;67(24):21781–94. doi: 10.1021/acs.jmedchem.4c01302 (PMC11684019; doi:10.1021/acs.jmedchem.4c01302)
Supplement: Supplementary file 1 — jm4c01302_si_001.pdf [file jm4c01302_si_001.pdf]

Supporting Information

Unveiling the Antimycobacterial Potential of Novel 4-Alkoxyquinolines: Insights into Selectivity, Mechanism of Action, and *In Vivo* Exposure

Fernanda Fries da Silva[a,b], Josiane Delgado Paz[a], Raoní Scheibler Rambo[a], Guilherme Arraché Gonçalves[a,c], Mauro Neves Muniz[a], Alexia de Matos Czeczot[a,c], Marcia Alberton Perelló[a], Andresa Berger[a,b], Laura Calle González[a,b], Lovaine Silva Duarte[a], Anelise Baptista da Silva[b,d], Carlos Alexandre Sanchez Ferreira[b,d], Sílvia Dias de Oliveira[b,d], Sidnei Moura[e], Cristiano Valim Bizarro[a,b], Luiz Augusto Basso[a,b,c], Pablo Machado[a,b,c,*]

[a]Instituto Nacional de Ciência e Tecnologia em Tuberculose, Centro de Pesquisas em Biologia Molecular e Funcional, Pontifícia Universidade Católica do Rio Grande do Sul, 90616-900, Porto Alegre, Rio Grande do Sul, Brazil

[b]Programa de Pós-Graduação em Biologia Celular e Molecular, Pontifícia Universidade Católica do Rio Grande do Sul, 90616-900, Porto Alegre, Rio Grande do Sul, Brazil

[c]Programa de Pós-Graduação em Medicina e Ciências da Saúde, Pontifícia Universidade Católica do Rio Grande do Sul, 90616-900, Porto Alegre, Rio Grande do Sul, Brazil

[d]Laboratório de Imunologia e Microbiologia, Pontifícia Universidade Católica do Rio Grande do Sul, 90616-900, Porto Alegre, Rio Grande do Sul, Brazil

[e]Laboratório de Biotecnologia de Produtos Naturais e Sintéticos, Instituto de Biotecnologia, Universidade de Caxias do Sul, 95070-560, Caxias do Sul, Rio Grande do Sul, Brazil

*E-mail: pablo.machado@pucrs.br (Pablo Machado)

TABLE OF CONTENTS

1. ^{1}H NMR and ^{13}C NMR spectra of synthesized compounds (**8a**–**w**)
2. UHPLC chromatogram for *in vivo* evaluated compound **8t**

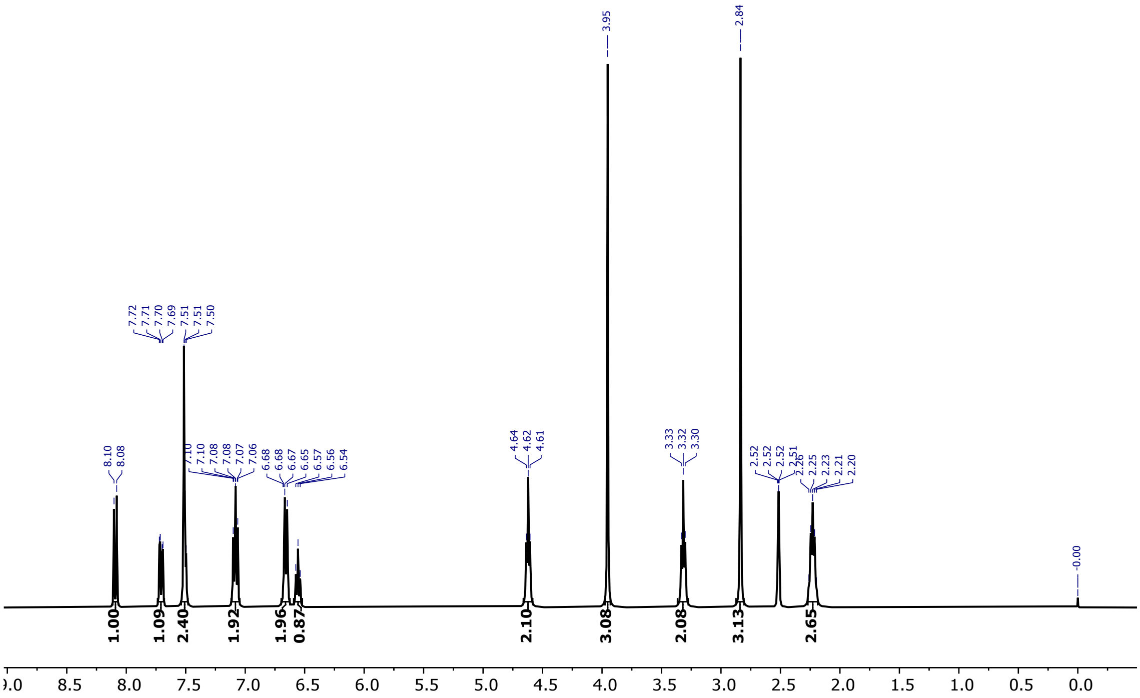

Figure 1. ^{1}H NMR (400 MHz, DMSO-d_6) spectra of compound **8a**.

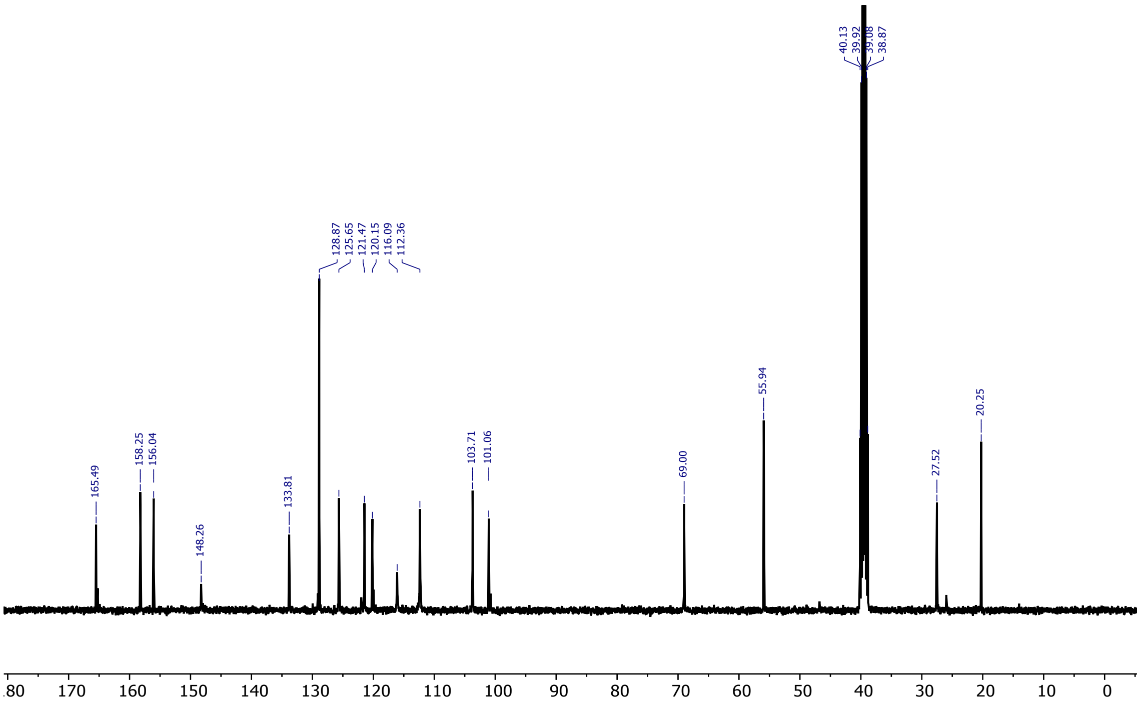

Figure 2. ^{13}C NMR (101 MHz, DMSO-d_6) spectra of compound **8a**.

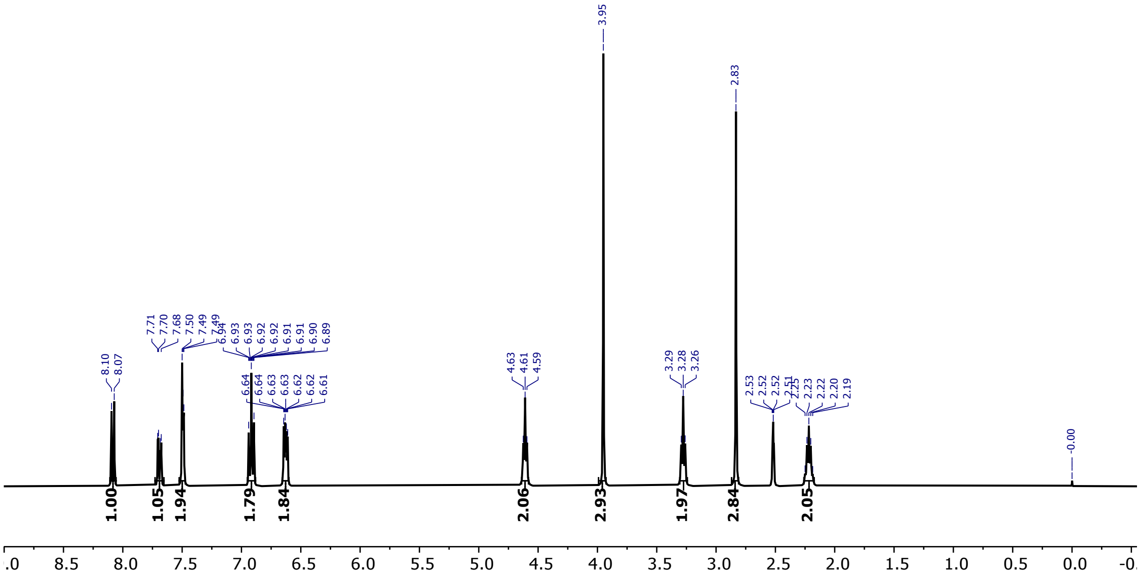

Figure 3. ^{1}H NMR (400 MHz, DMSO-d_6) spectra of compound **8b**.

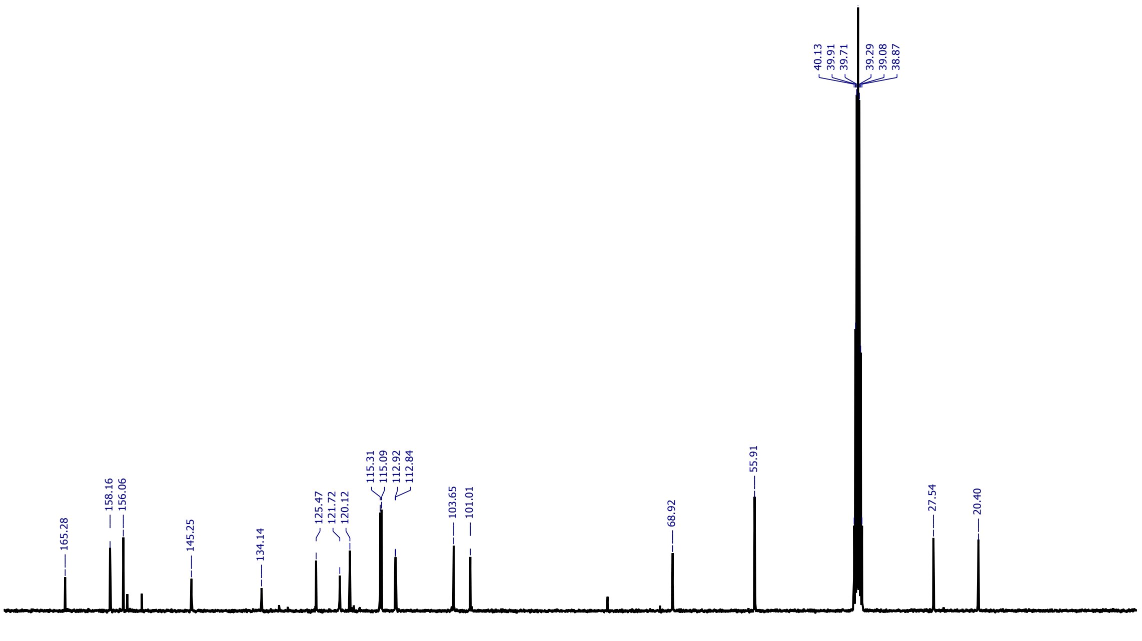

Figure 4. ^{13}C NMR (101 MHz, DMSO-d_6) spectra of compound **8b**.

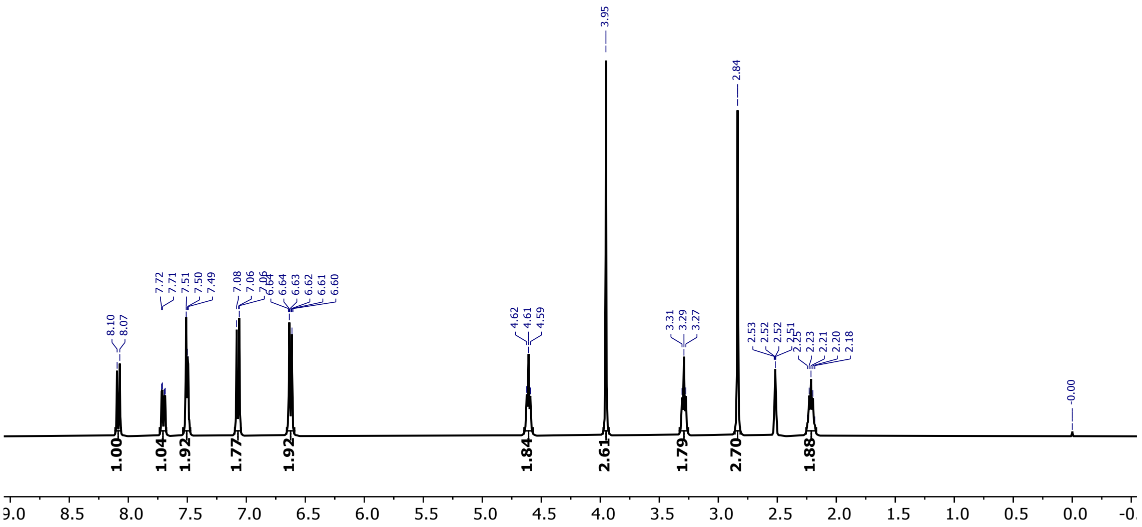

Figure 5. ^{1}H NMR (400 MHz, DMSO-d_6) spectra of compound **8c**.

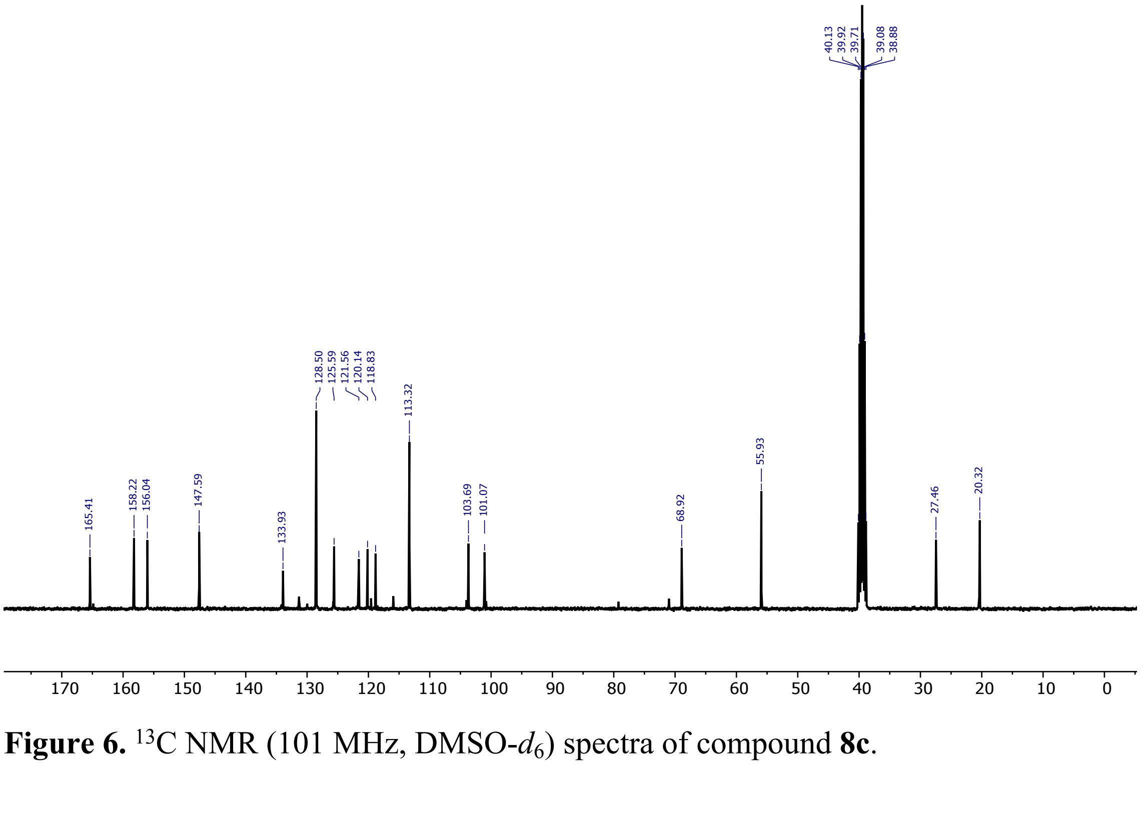

Figure 6. ^{13}C NMR (101 MHz, DMSO-d_6) spectra of compound **8c**.

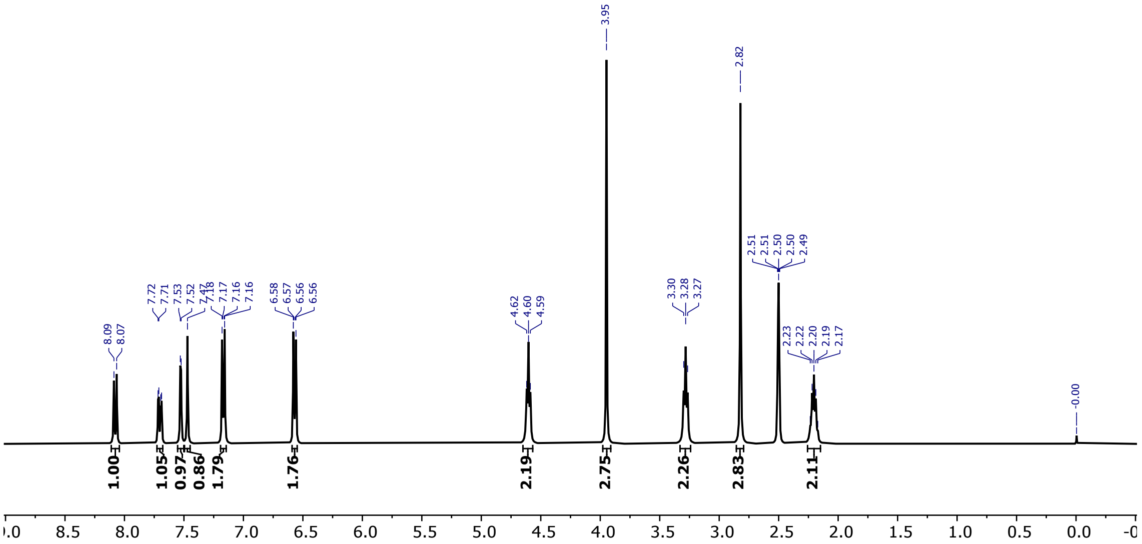

Figure 7. ^{1}H NMR (400 MHz, DMSO-d_6) spectra of compound **8d**.

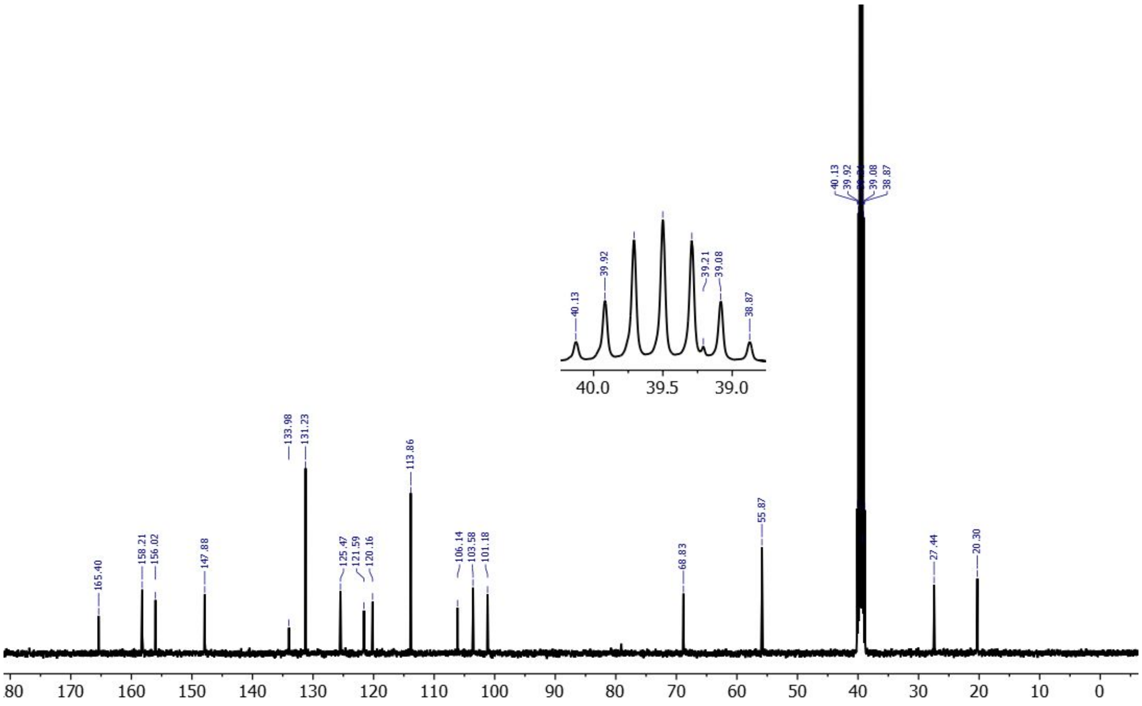

Figure 8. ^{13}C NMR (101 MHz, DMSO-d_6) spectra of compound **8d**.

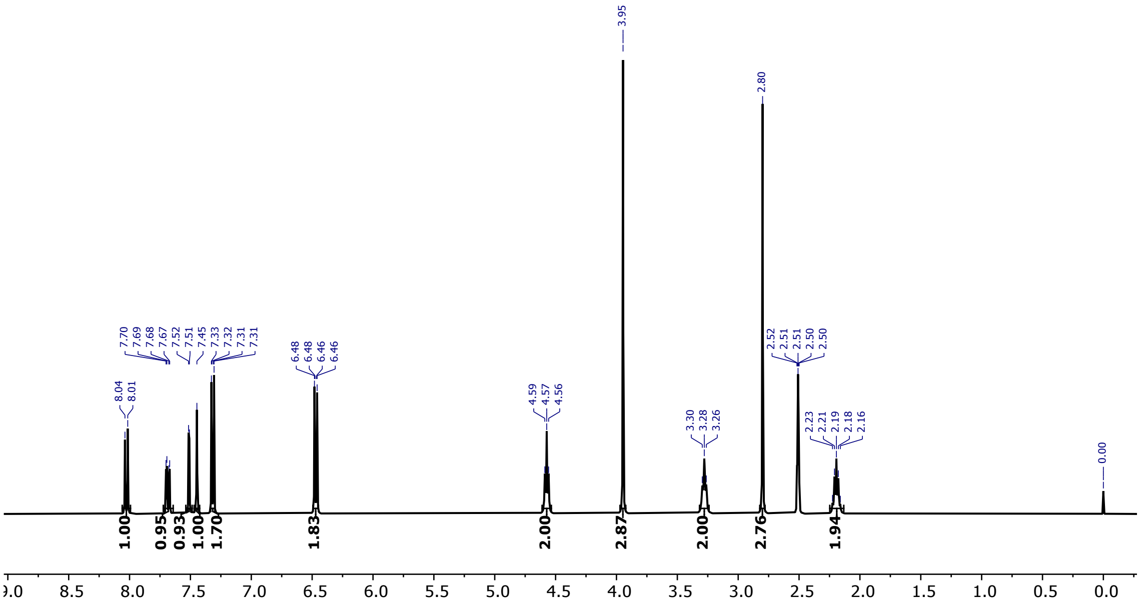

Figure 9. ^{1}H NMR (400 MHz, DMSO-d_6) spectra of compound **8e**.

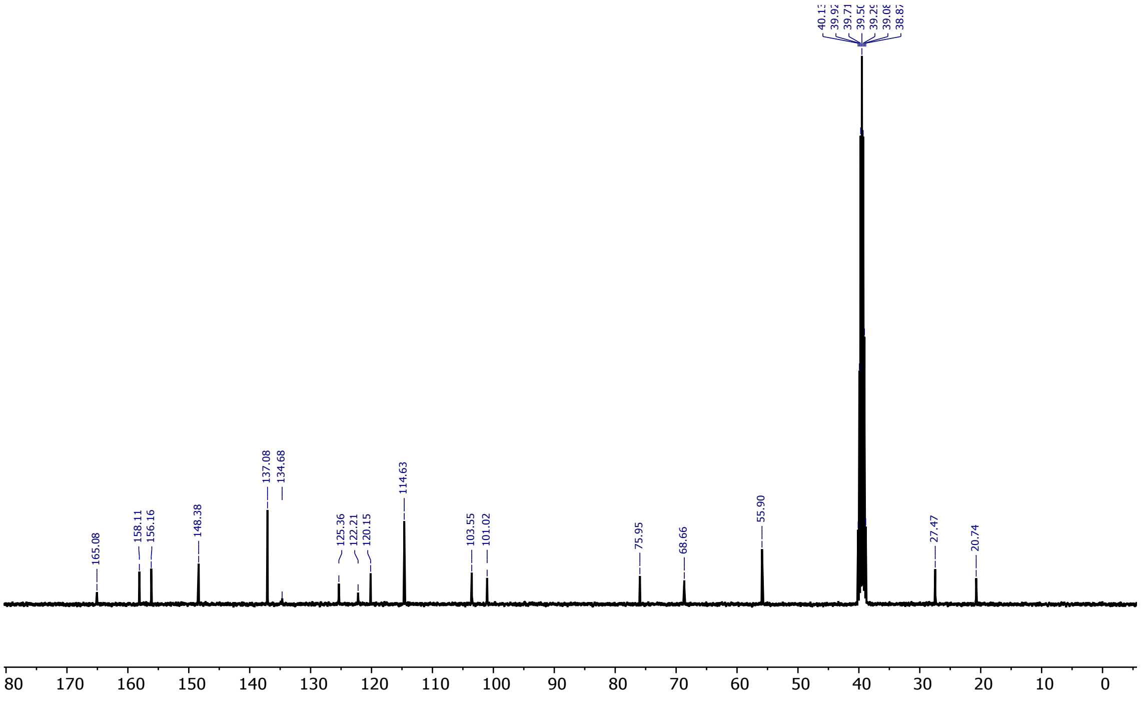

Figure 10. ^{13}C NMR (101 MHz, DMSO-d_6) spectra of compound **8e**.

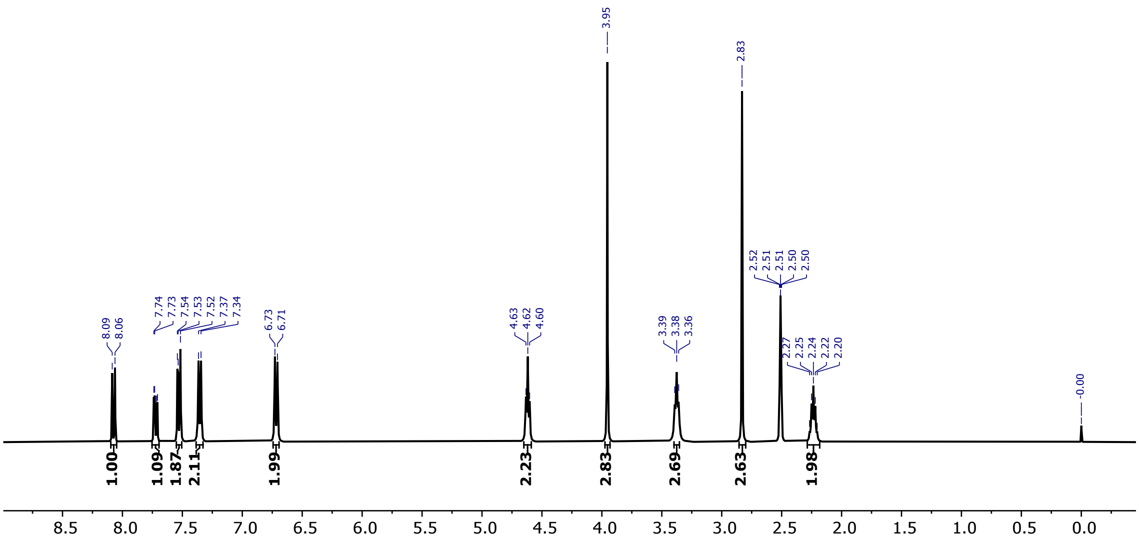

Figure 11. ^{1}H NMR (400 MHz, DMSO-d_6) spectra of compound **8f**.

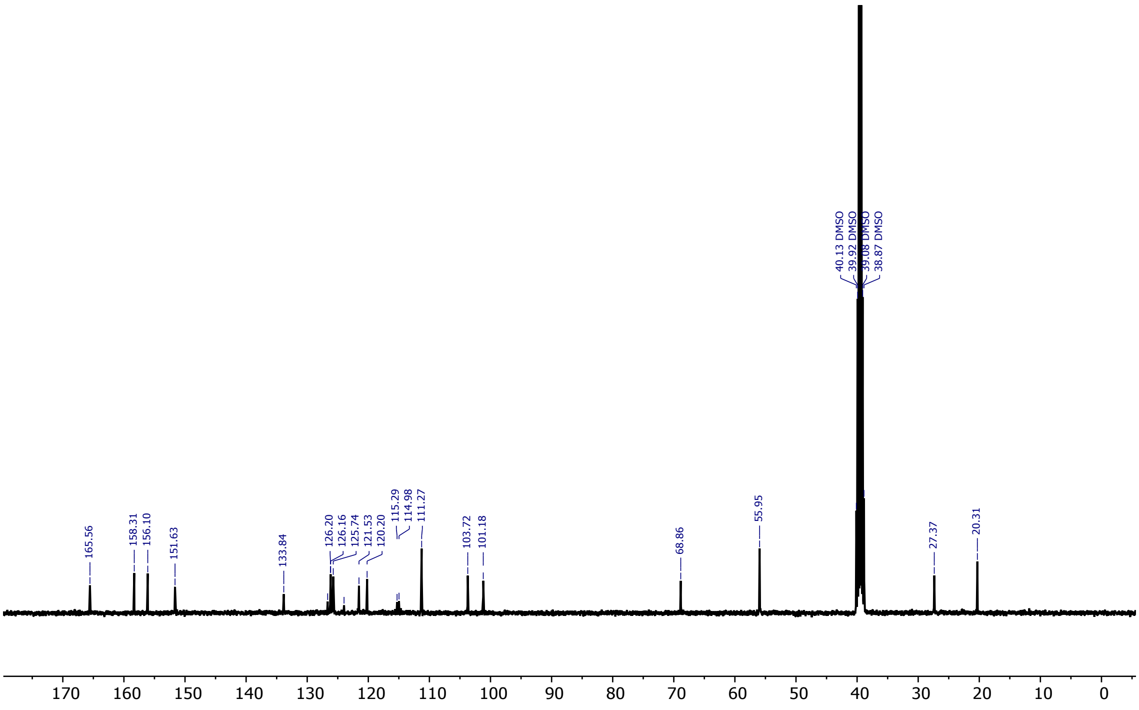

Figure 12. ^{13}C NMR (101 MHz, DMSO-d_6) spectra of compound **8f**.

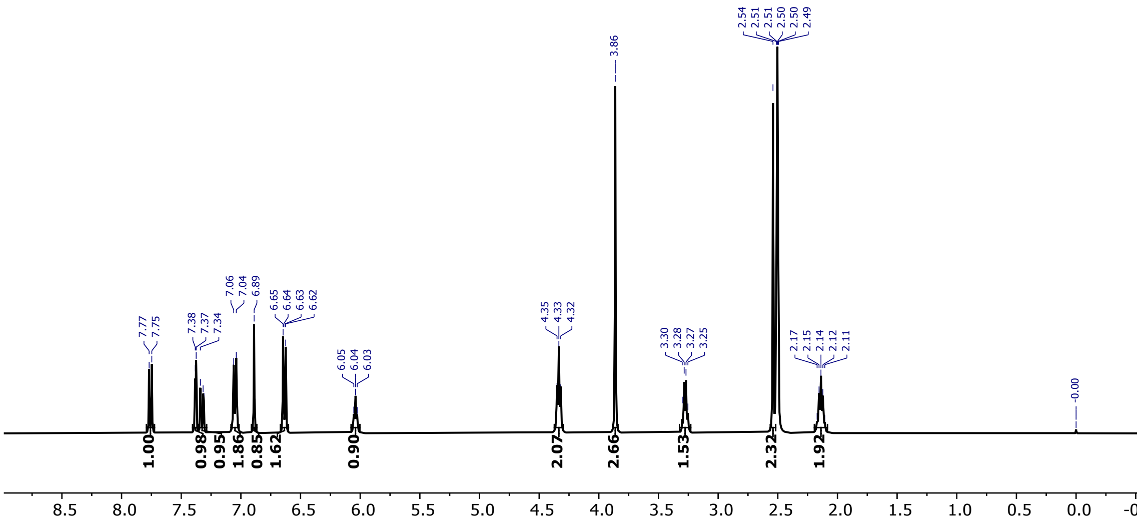

Figure 13. ^{1}H NMR (400 MHz, DMSO-d_6) spectra of compound **8g**.

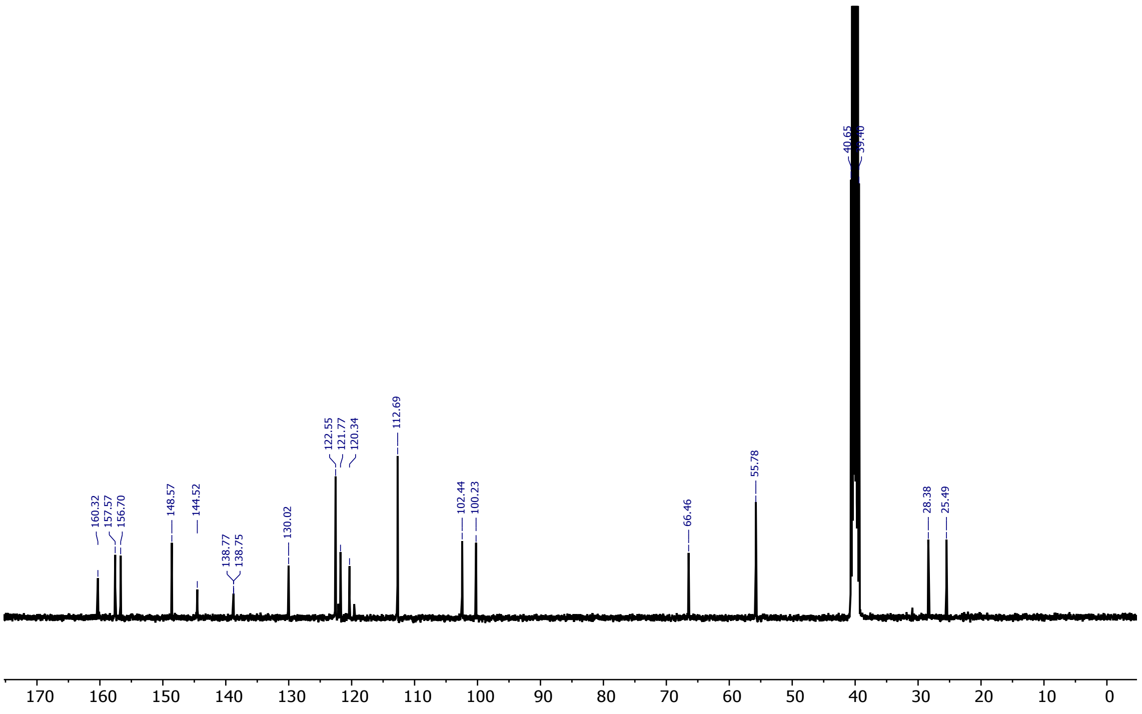

Figure 14. ^{13}C NMR (101 MHz, DMSO-d_6) spectra of compound **8g**.

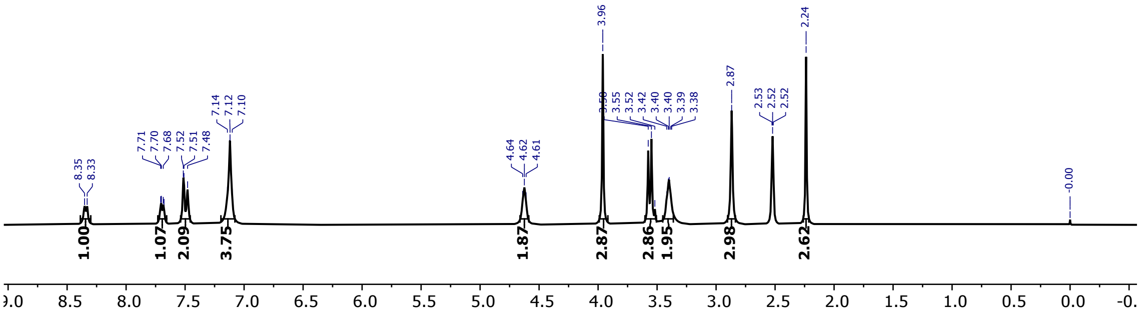

Figure 15. ^{1}H NMR (400 MHz, DMSO-d_6) spectra of compound **8h**.

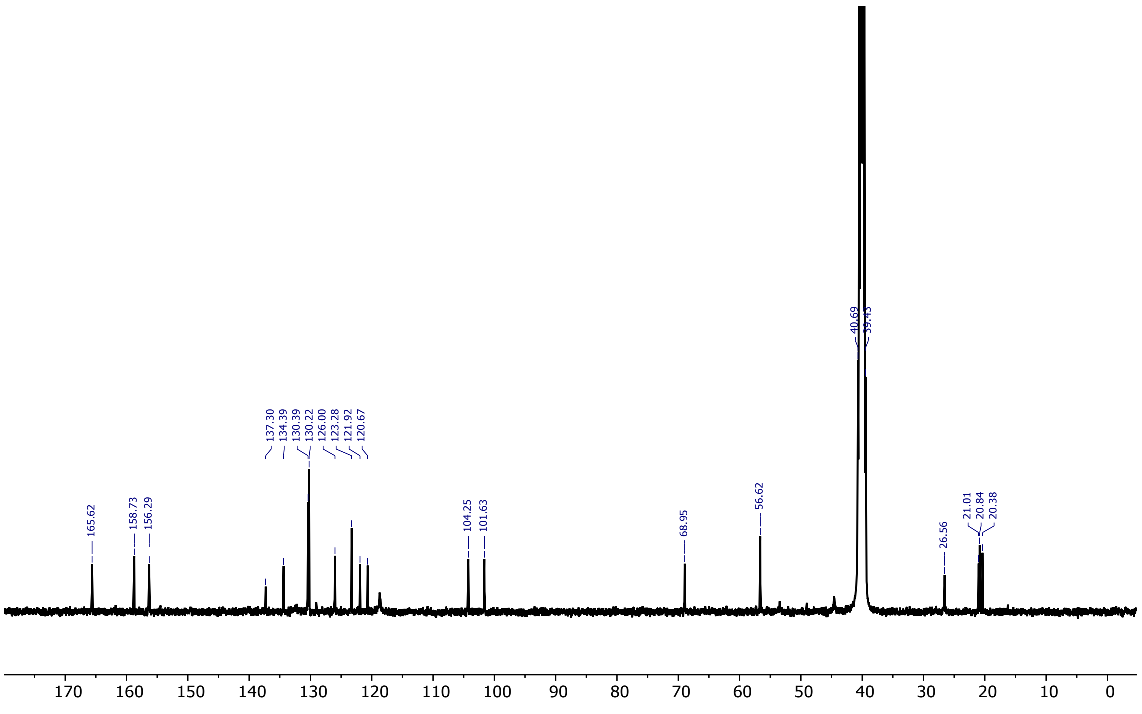

Figure 16. ^{13}C NMR (101 MHz, DMSO-d_6) spectra of compound **8h**.

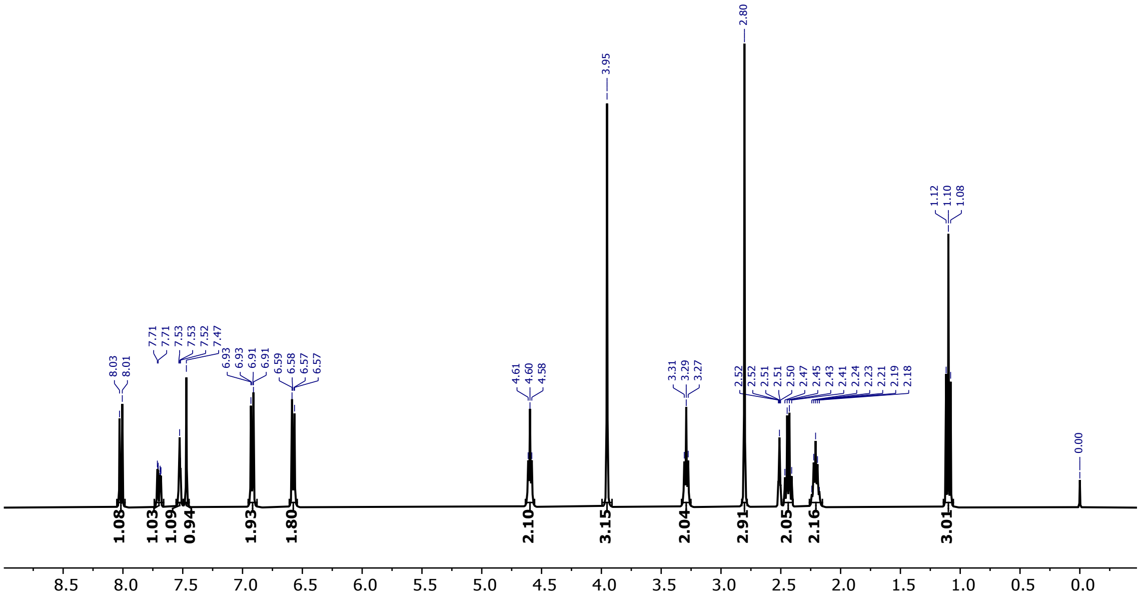

Figure 17. ^{1}H NMR (400 MHz, DMSO-d_6) spectra of compound **8i**.

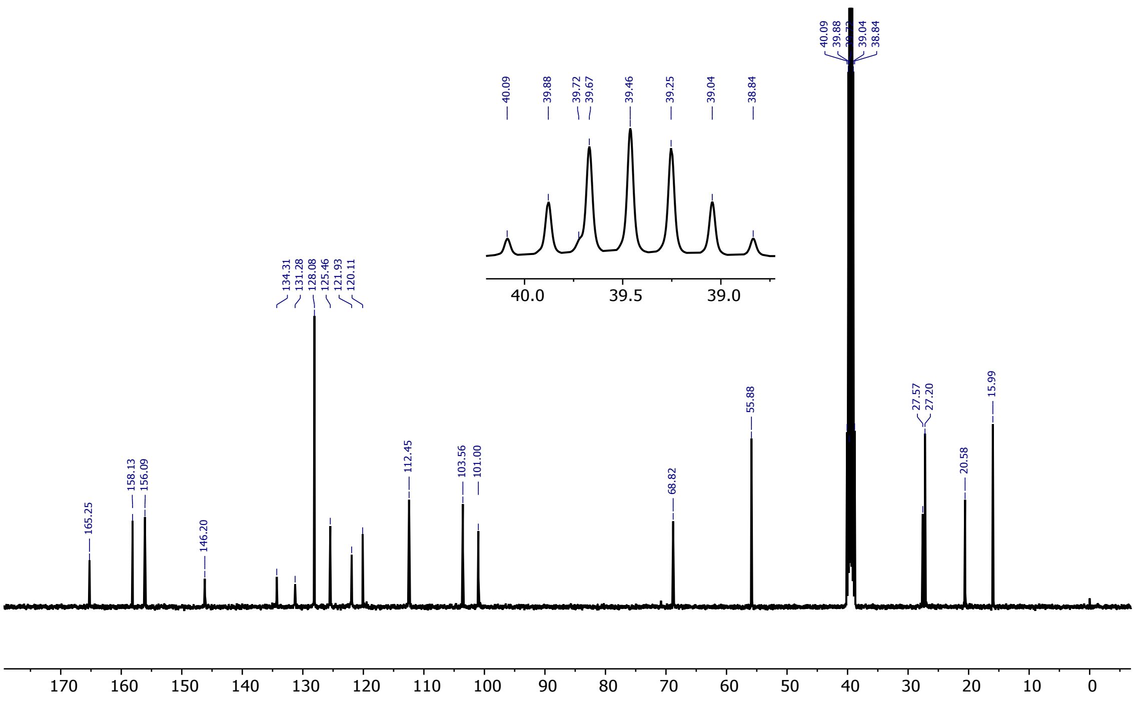

Figure 18. ^{13}C NMR (101 MHz, DMSO-d_6) spectra of compound **8i**.

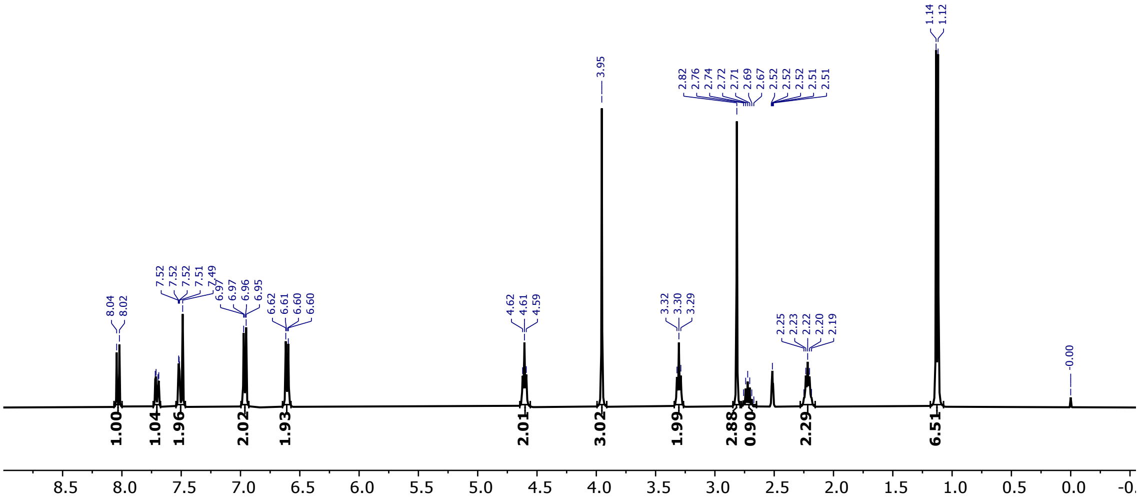

Figure 19. ^{1}H NMR (400 MHz, DMSO-d_6) spectra of compound **8j**.

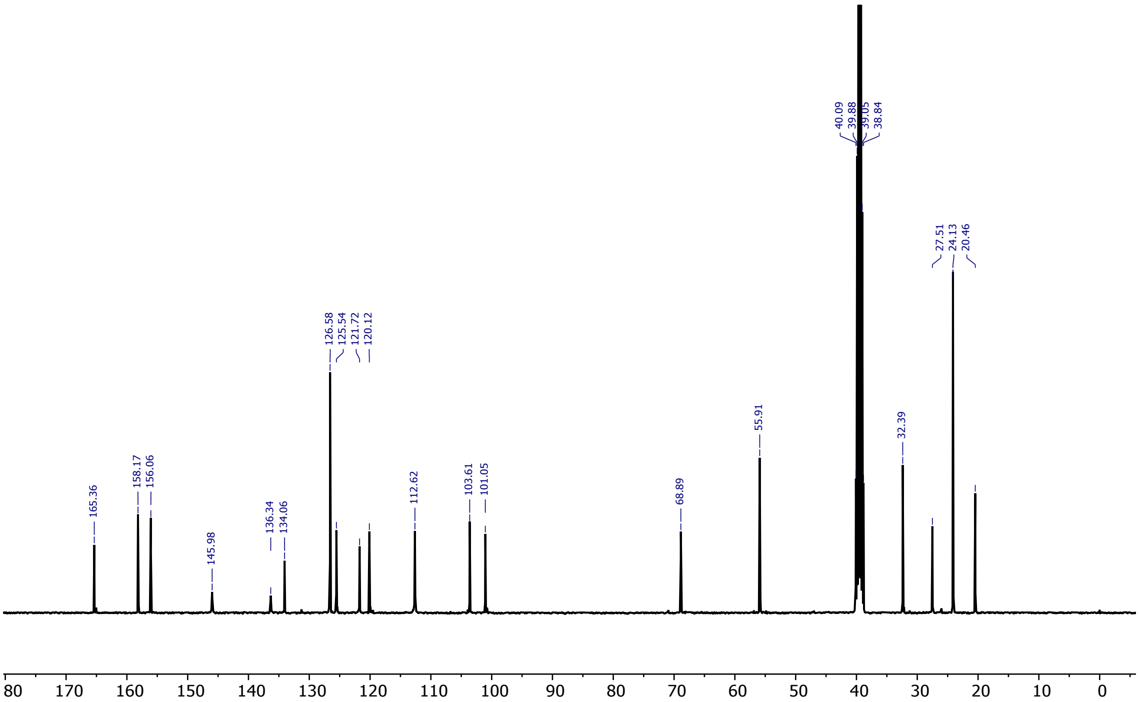

Figure 20. ^{13}C NMR (101 MHz, DMSO-d_6) spectra of compound **8j**.

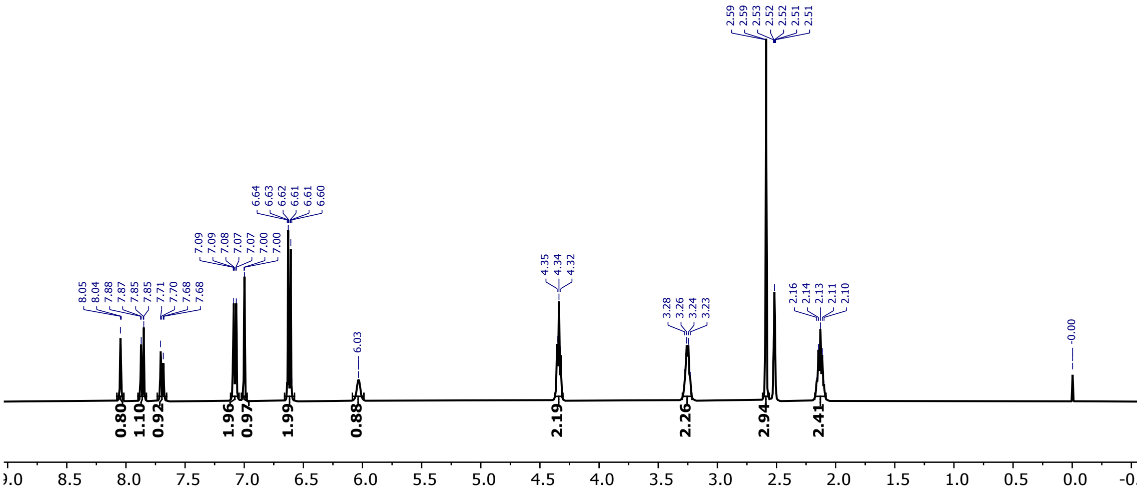

Figure 21. ^{1}H NMR (400 MHz, DMSO-d_6) spectra of compound **8k**.

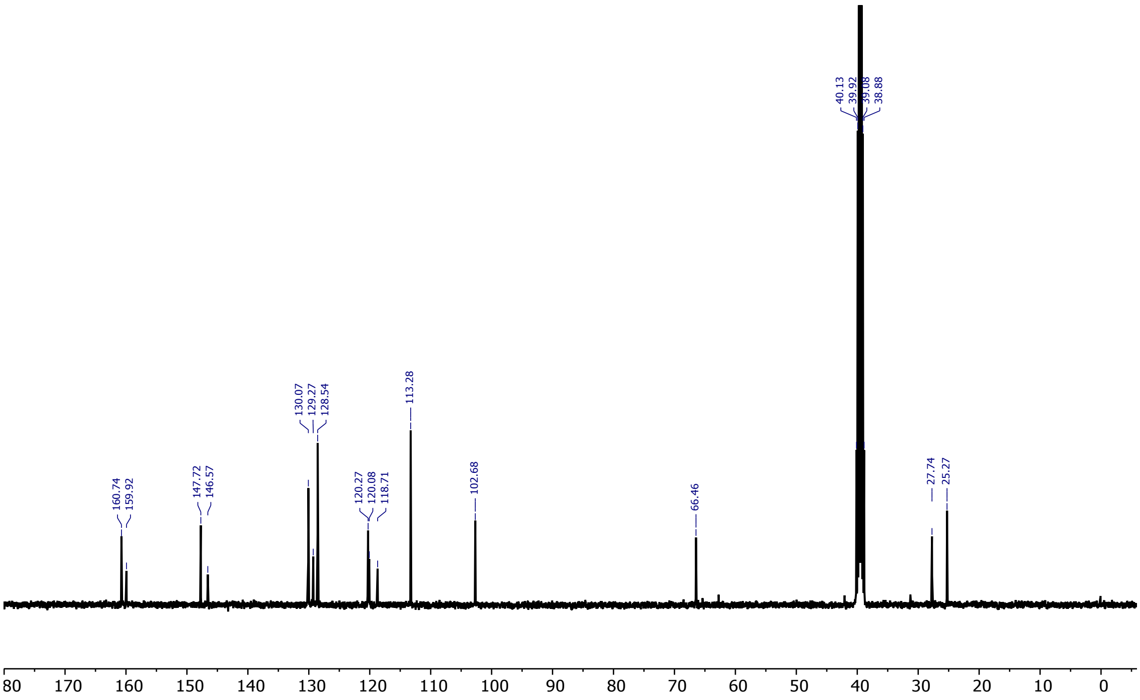

Figure 22. ^{13}C NMR (101 MHz, DMSO-d_6) spectra of compound **8k**.

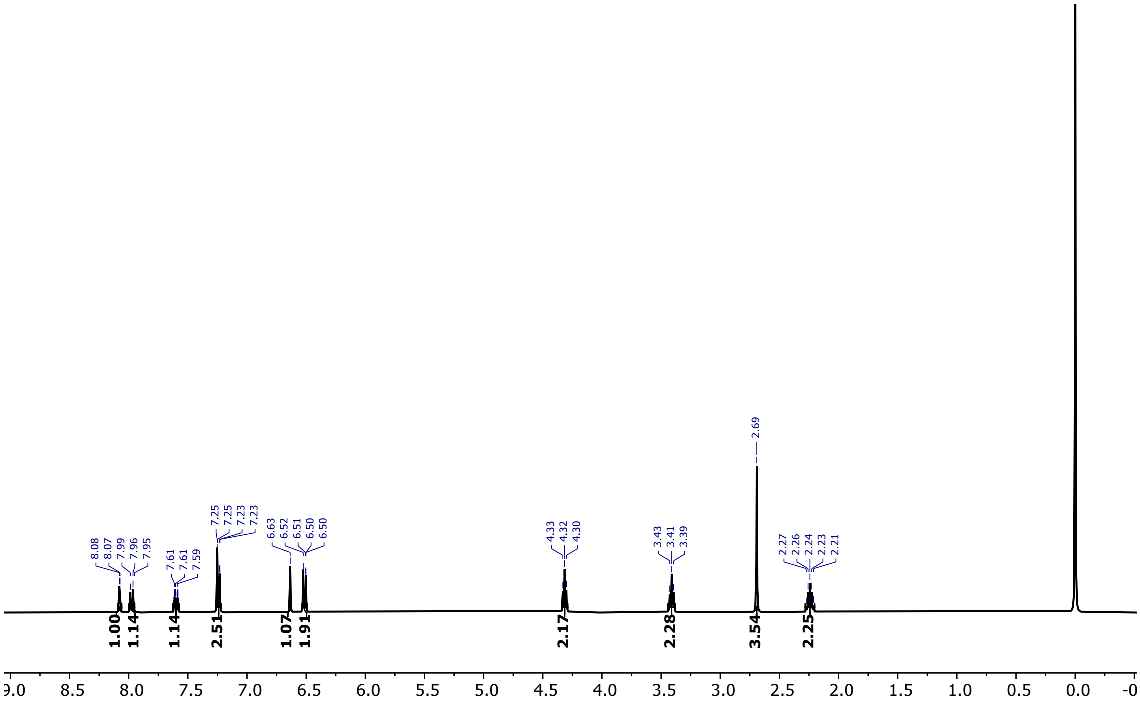

Figure 23. ^{1}H NMR (400 MHz, Chloroform-*d*) spectra of compound **8l**.

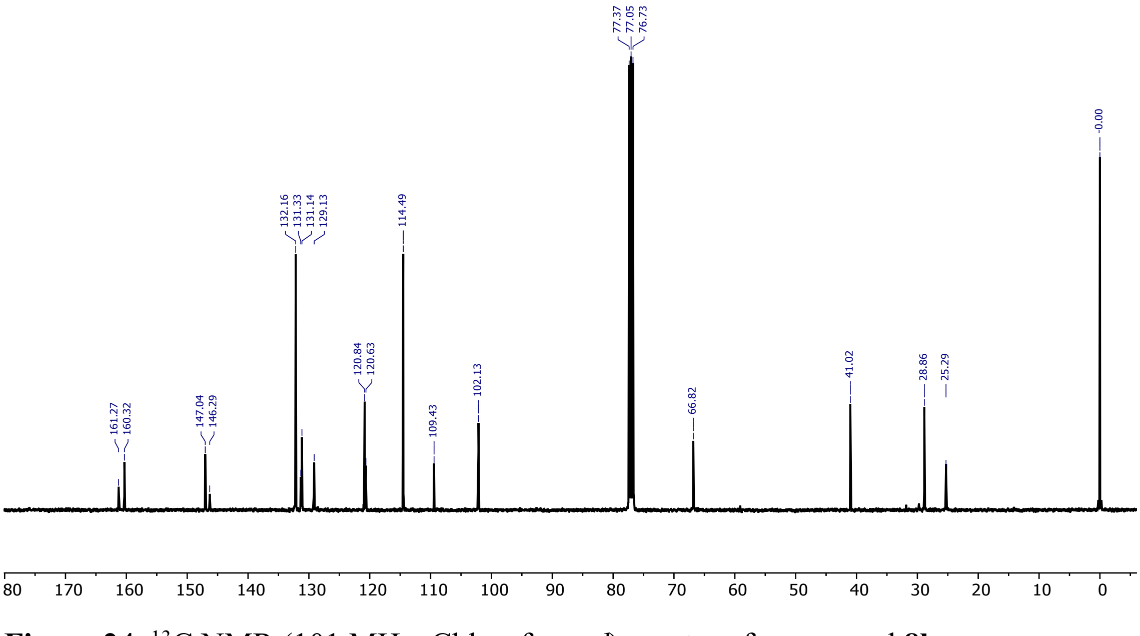

Figure 24. ^{13}C NMR (101 MHz, Chloroform-*d*) spectra of compound **8l**.

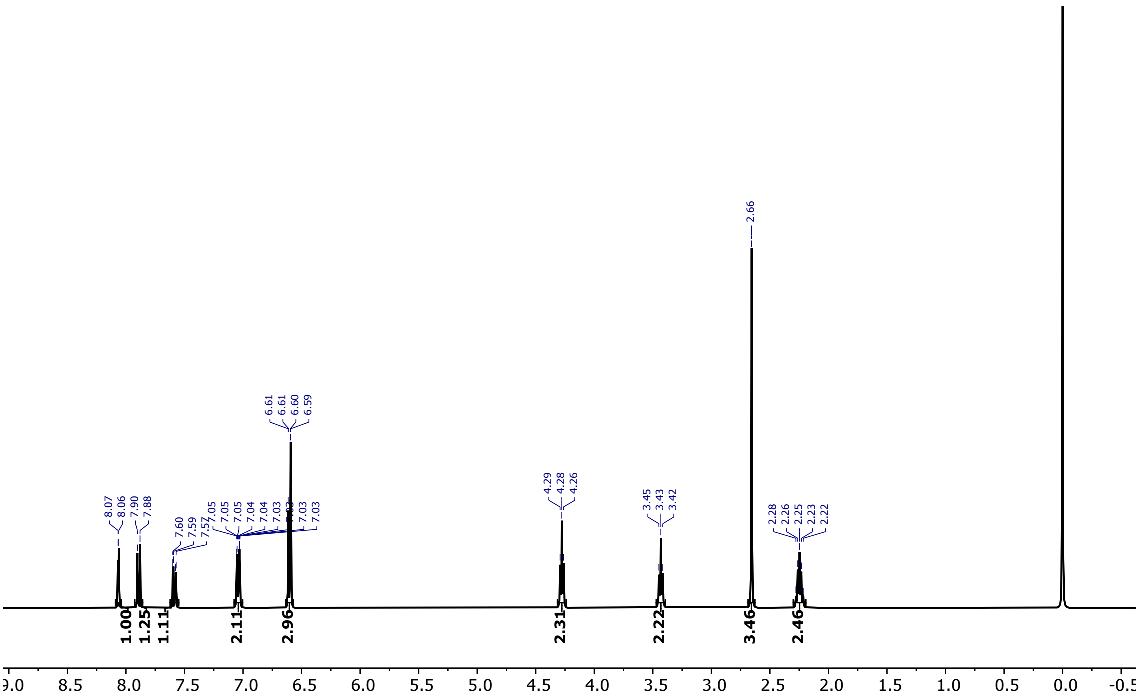

Figure 21. ^{1}H NMR (400 MHz, Chloroform-*d*) spectra of compound **8m**.

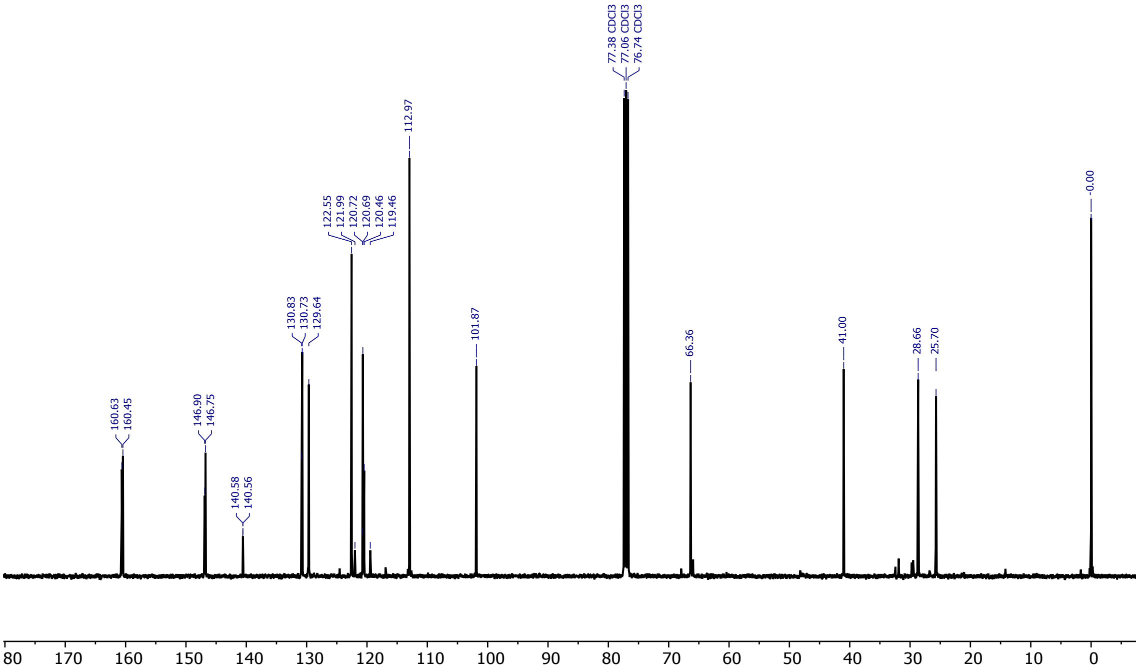

Figure 22. ^{13}C NMR (101 MHz, Chloroform-*d*) spectra of compound **8m**.

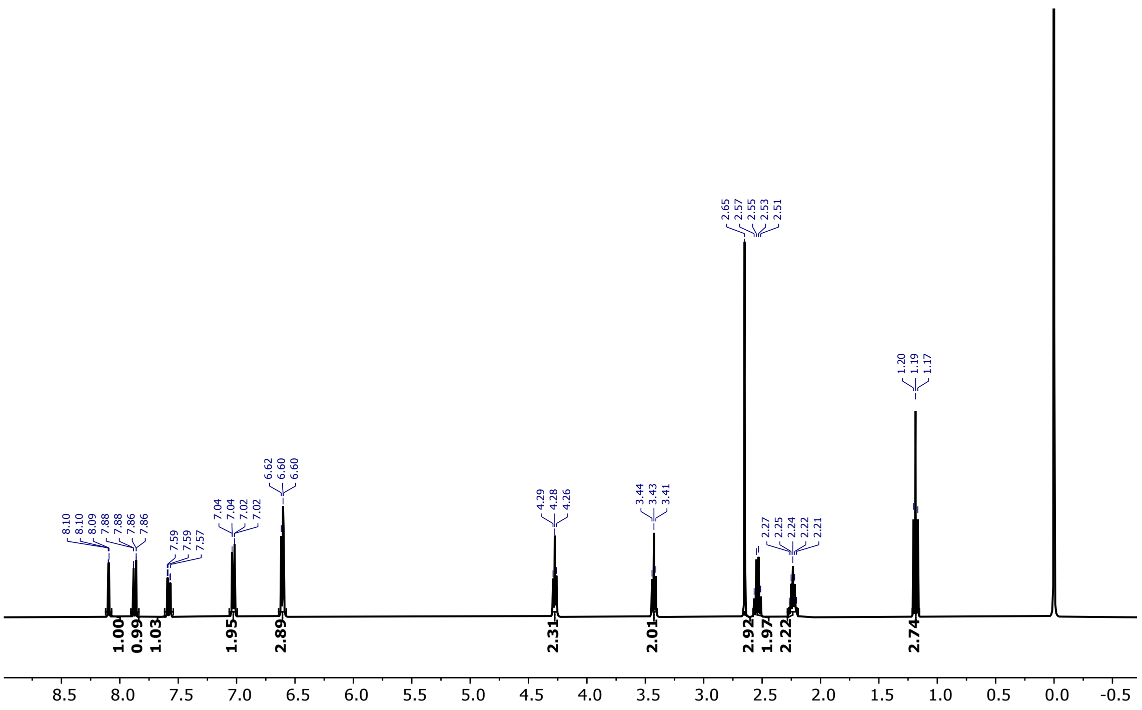

Figure 23. ^{1}H NMR (400 MHz, Chloroform-*d*) spectra of compound **8n**.

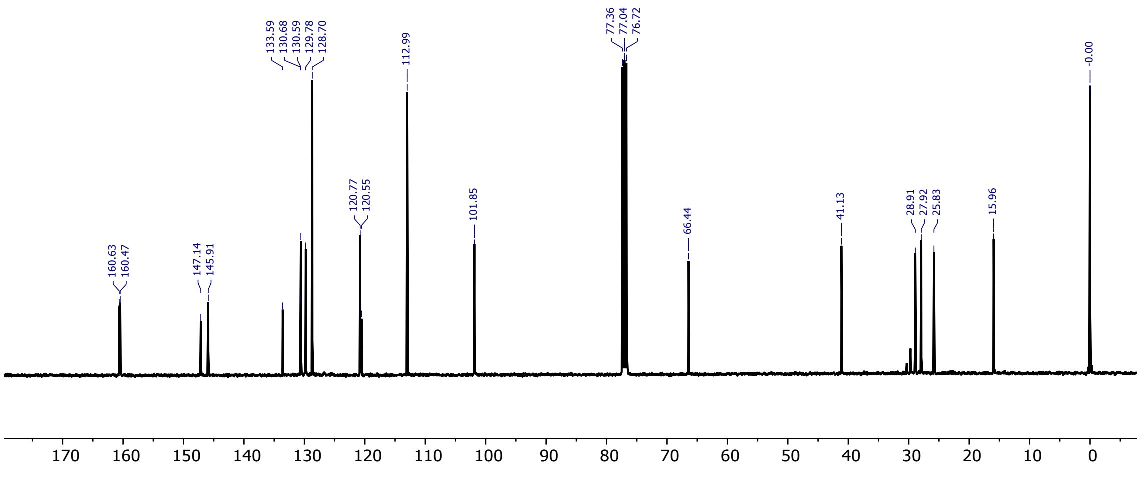

Figure 24. ^{13}C NMR (101 MHz, Chloroform-*d*) spectra of compound **8n**.

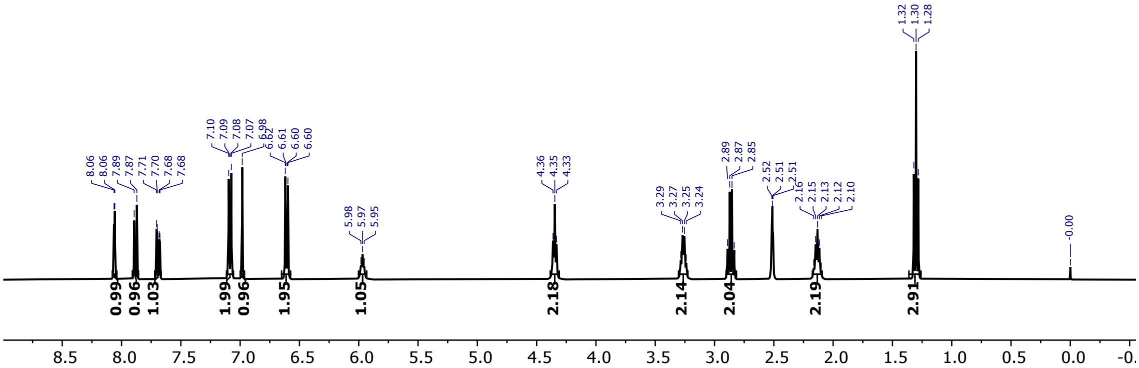

Figure 25. ^{1}H NMR (400 MHz, DMSO-d_6) spectra of compound **8o**.

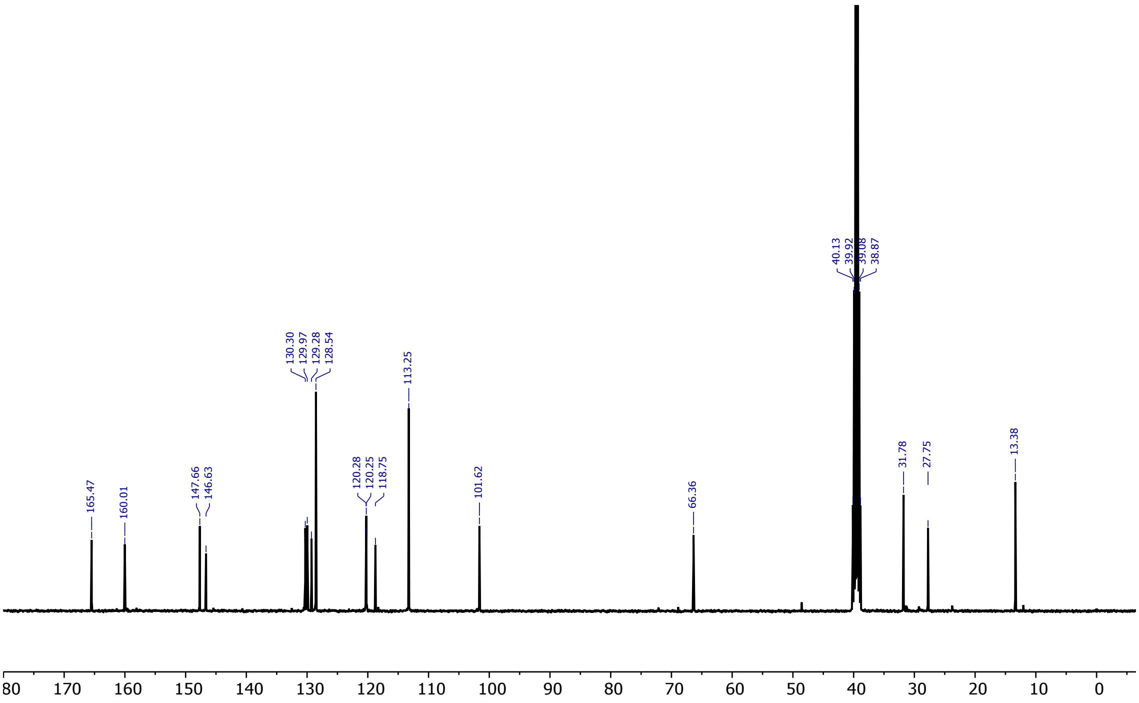

13C NMR (101 MHz, DMSO-d_6) δ 13.38, 27.75, 31.78, 66.36, 101.62, 113.25 (2C), 118.75, 120.25, 120.28, 128.54 (2C), 129.28, 129.97, 130.30, 146.63, 147.66, 160.01, 165.47.

Figure 26. ^{13}C NMR (101 MHz, DMSO-d_6) spectra of compound **8o**.

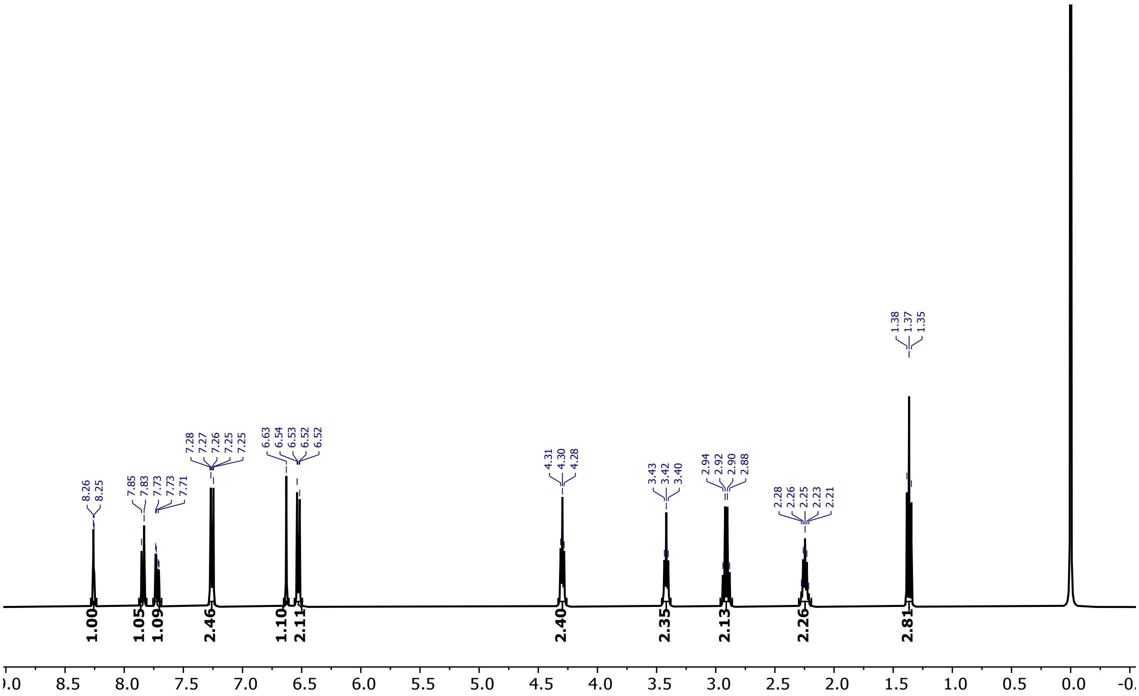

Figure 27. ^{13}H NMR (400 MHz, Chloroform-*d*) spectra of compound **8p**.

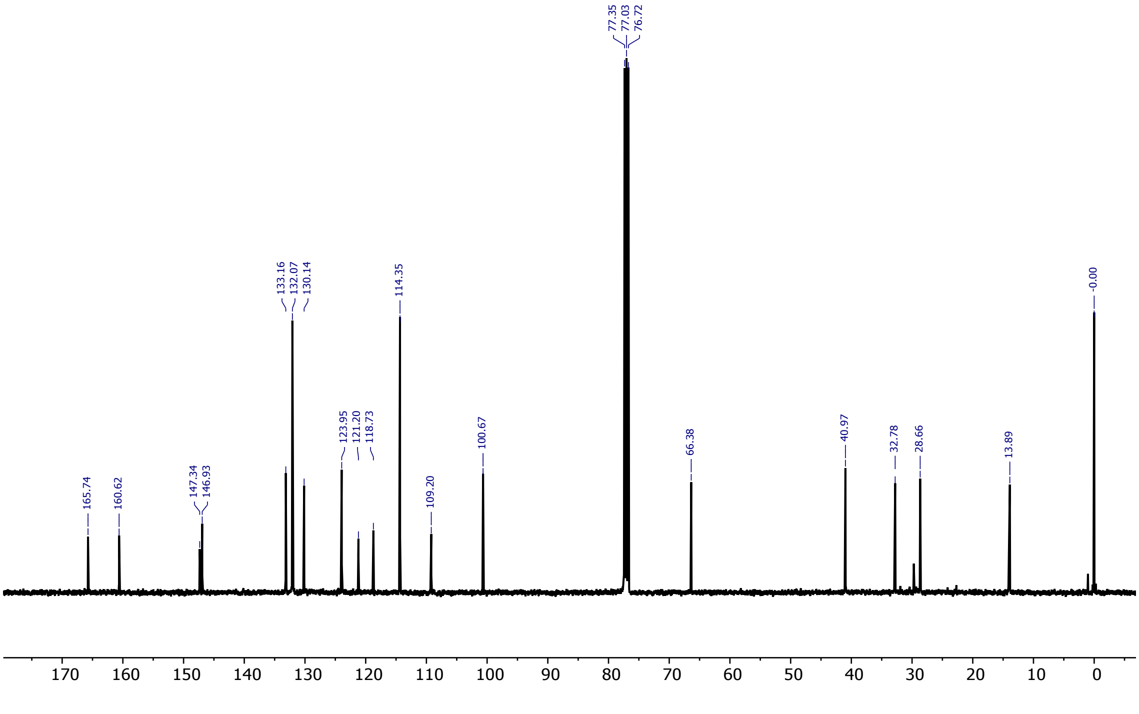

Figure 28. ^{13}C NMR (101 MHz, Chloroform-*d*) spectra of compound **8p**.

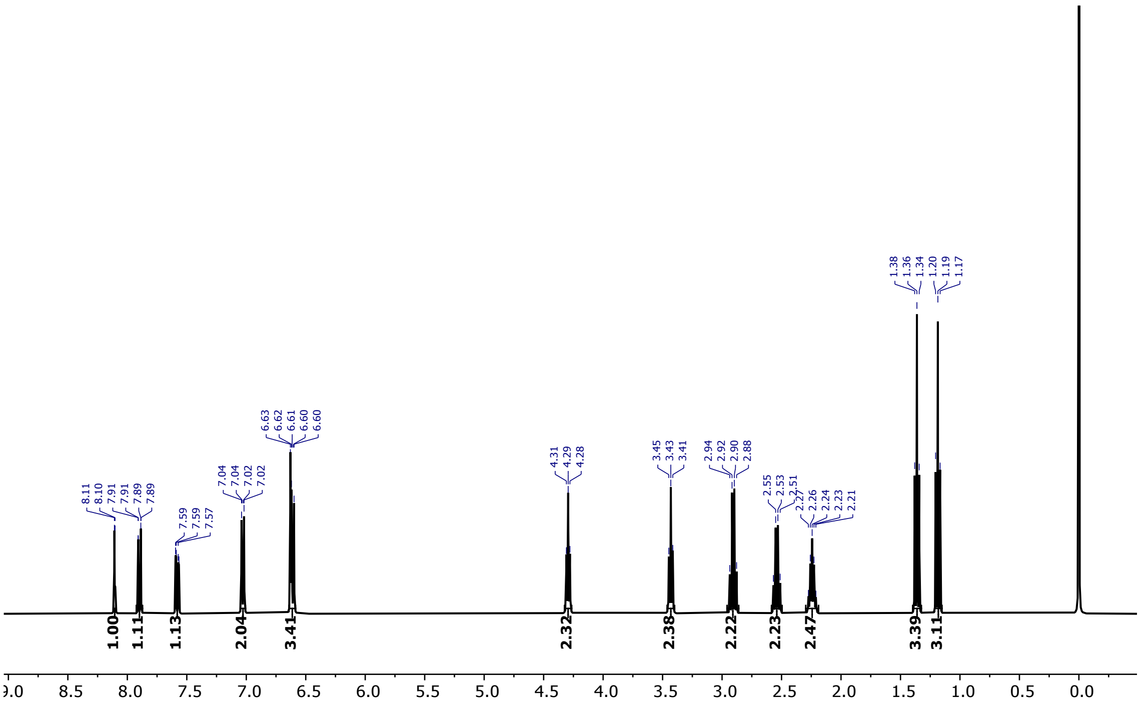

Figure 29. ^{1}H NMR (400 MHz, Chloroform-*d*) spectra of compound **8q**.

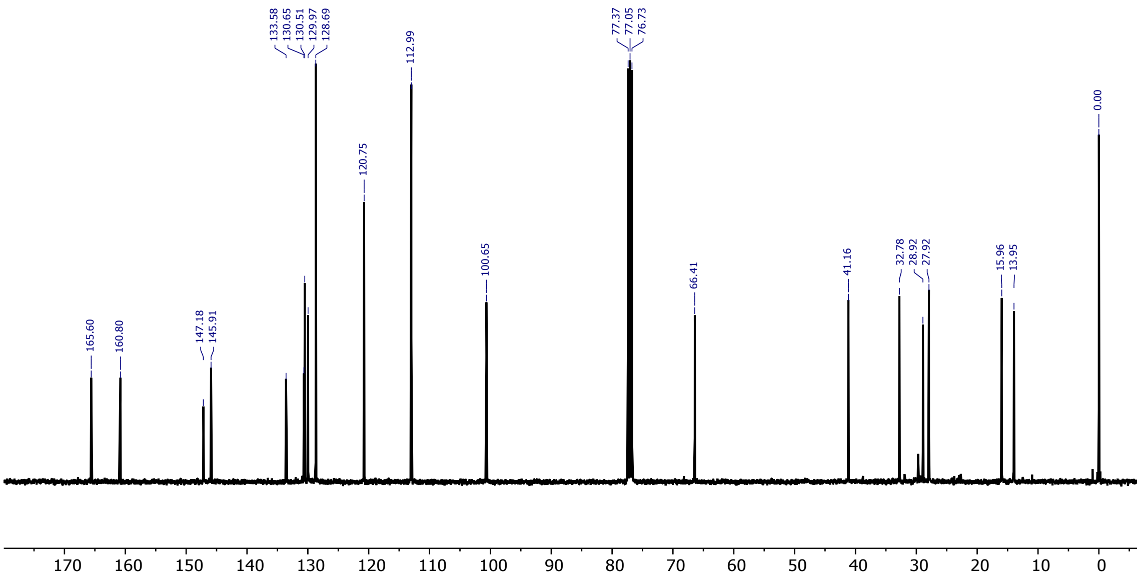

Figure 30. ^{13}C NMR (101 MHz, Chloroform-*d*) spectra of compound **8q**.

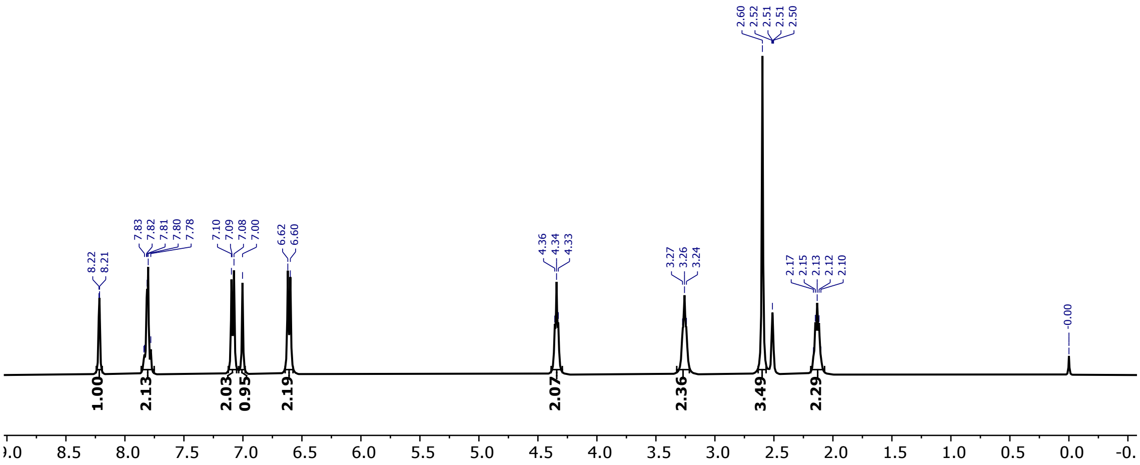

Figure 31. ^{1}H NMR (400 MHz, DMSO-d_6) spectra of compound **8r**.

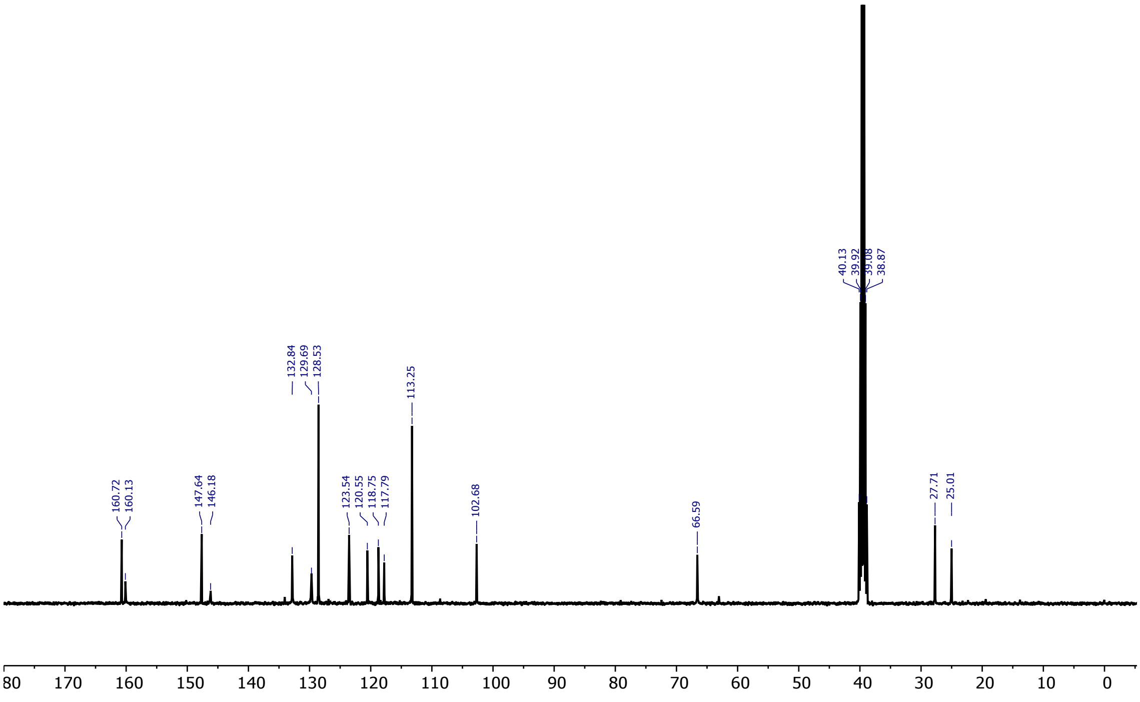

Figure 32. ^{13}C NMR (101 MHz, DMSO-d_6) spectra of compound **8r**.

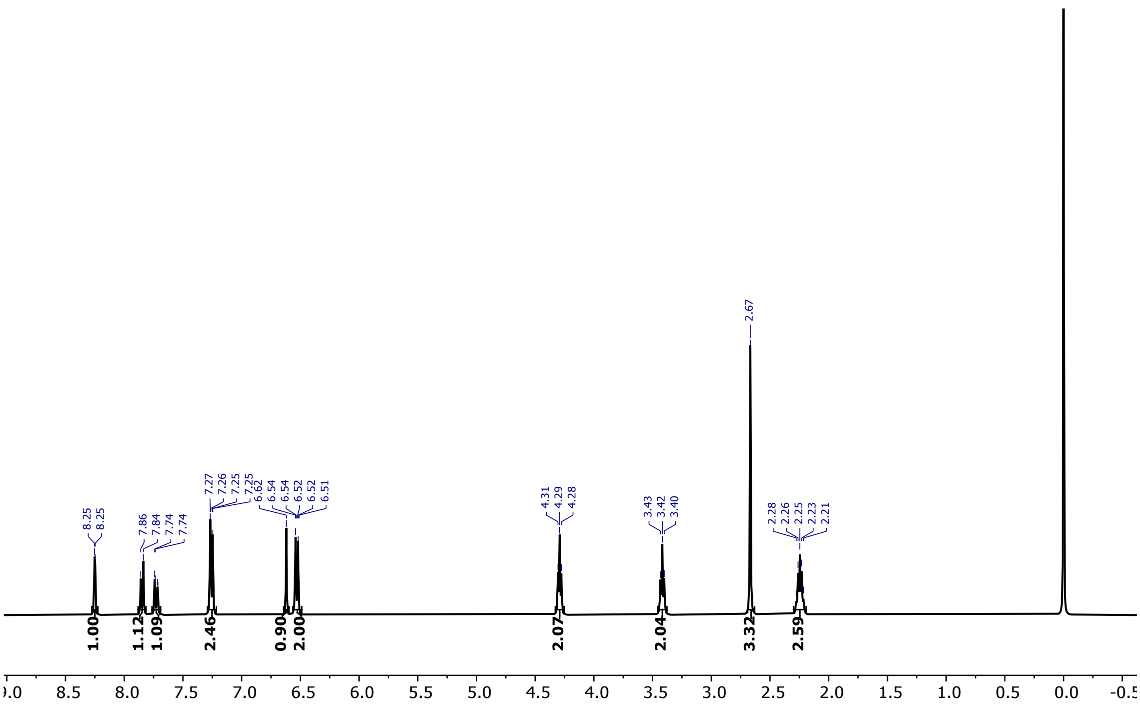

Figure 33. ^{13}H NMR (400 MHz, $CDCl_3$) spectra of compound **8s**.

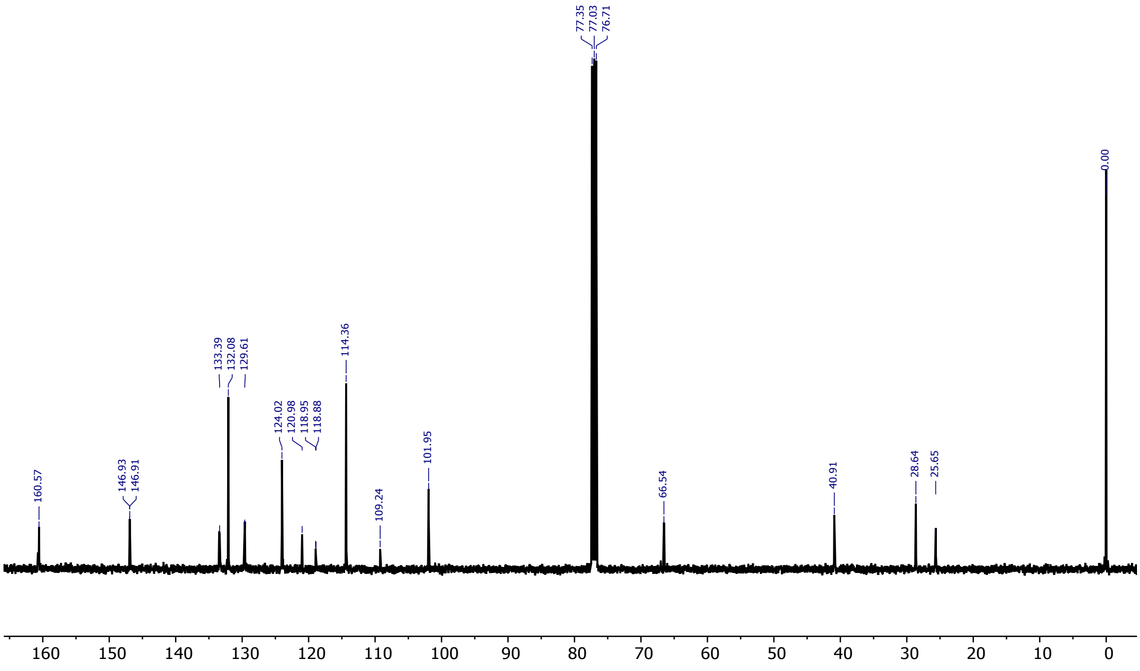

Figure 34. ^{13}C NMR (101 MHz, $CDCl_3$) spectra of compound **8s**.

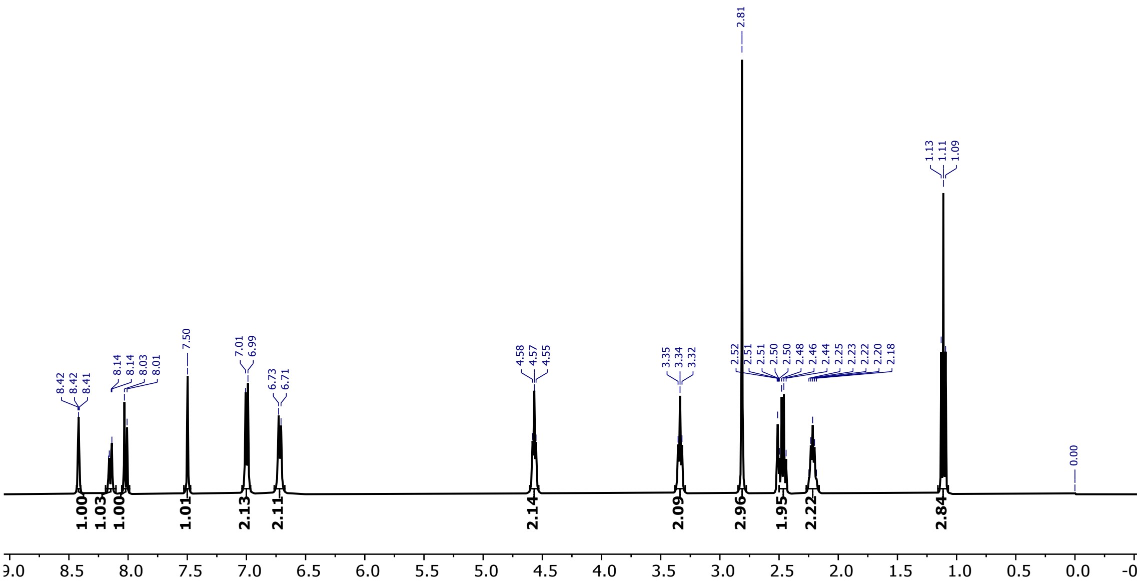

Figure 35. ^{1}H NMR (400 MHz, DMSO-d_6) spectra of compound **8t**.

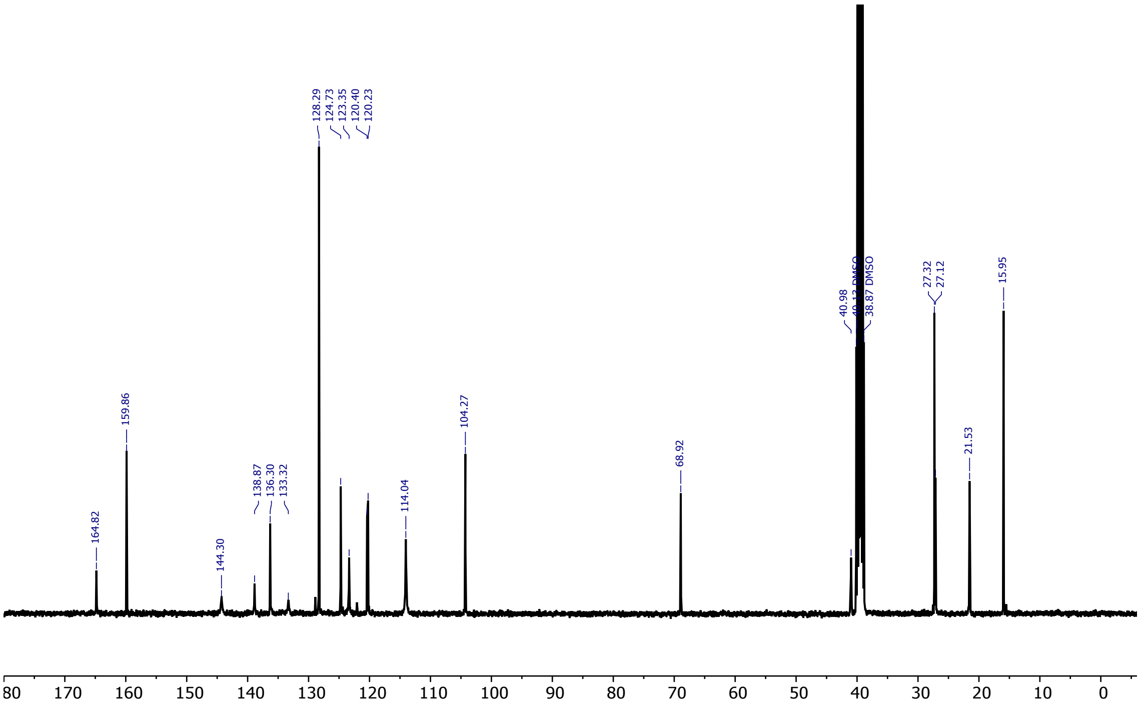

Figure 36. ^{13}C NMR (101 MHz, DMSO-d_6) spectra of compound **8t**.

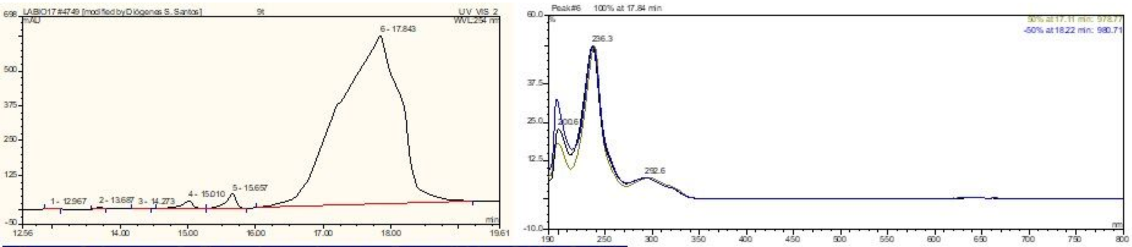

No.	Peakname	Ret.Time min	Area mAU*min	Amount	Type	Height mAU	Rel.Area %	Resolution
1	n.a.	12.967	0.2192	n.a.	BMB*	1.629	0.03	3.98
2	n.a.	13.687	0.6012	n.a.	BMB	5.899	0.09	2.33
3	n.a.	14.273	0.2892	n.a.	BMB*	1.798	0.04	2.43
4	n.a.	15.010	5.8111	n.a.	BM	26.603	0.90	2.52
5	n.a.	15.657	9.1626	n.a.	MB	51.744	1.42	2.05
6	n.a.	17.843	630.3494	n.a.	BMB*	604.210	97.51	n.a.
Total:			646.4326	0.0000		691.882	100.00	

Figure 37. UHPL chromatogram of compound **8t**.

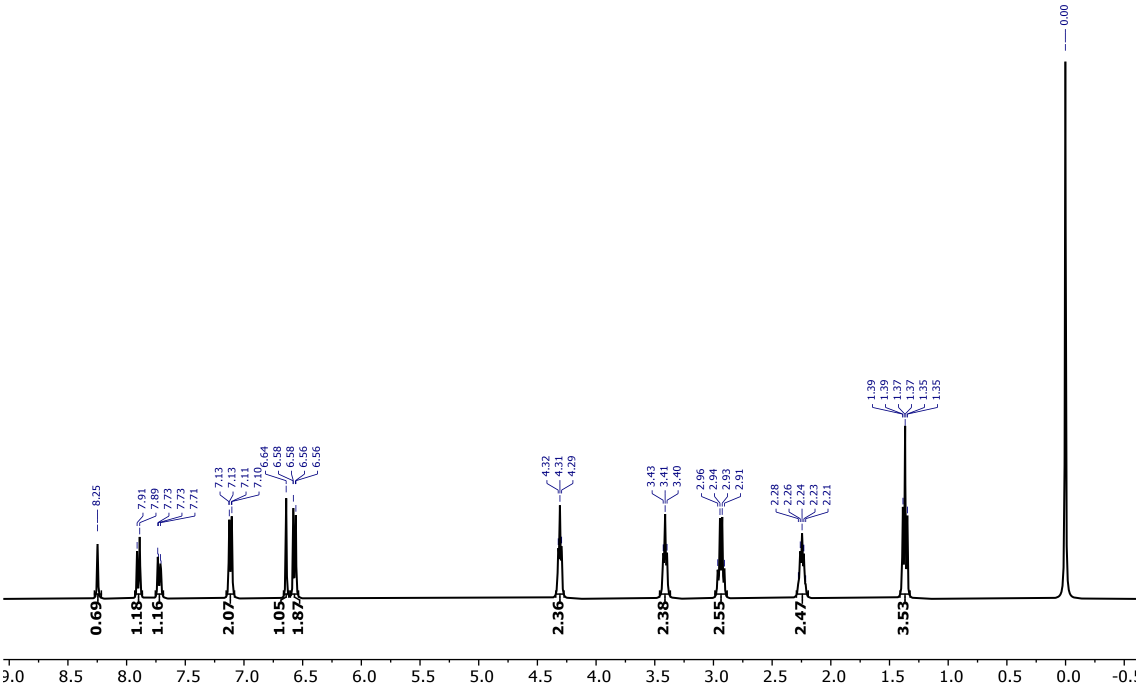

Figure 38. ^{1}H NMR (400 MHz, $CDCl_3$) spectra of compound **8u**.

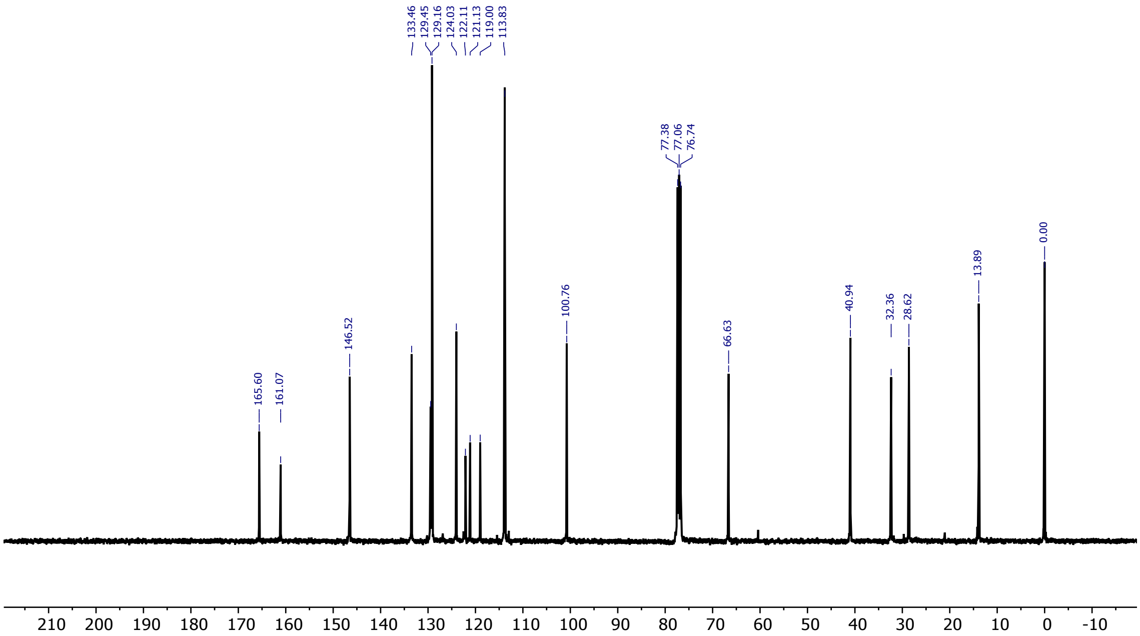

Figure 39. ^{13}C NMR (101 MHz, $CDCl_3$) spectra of compound **8u**.

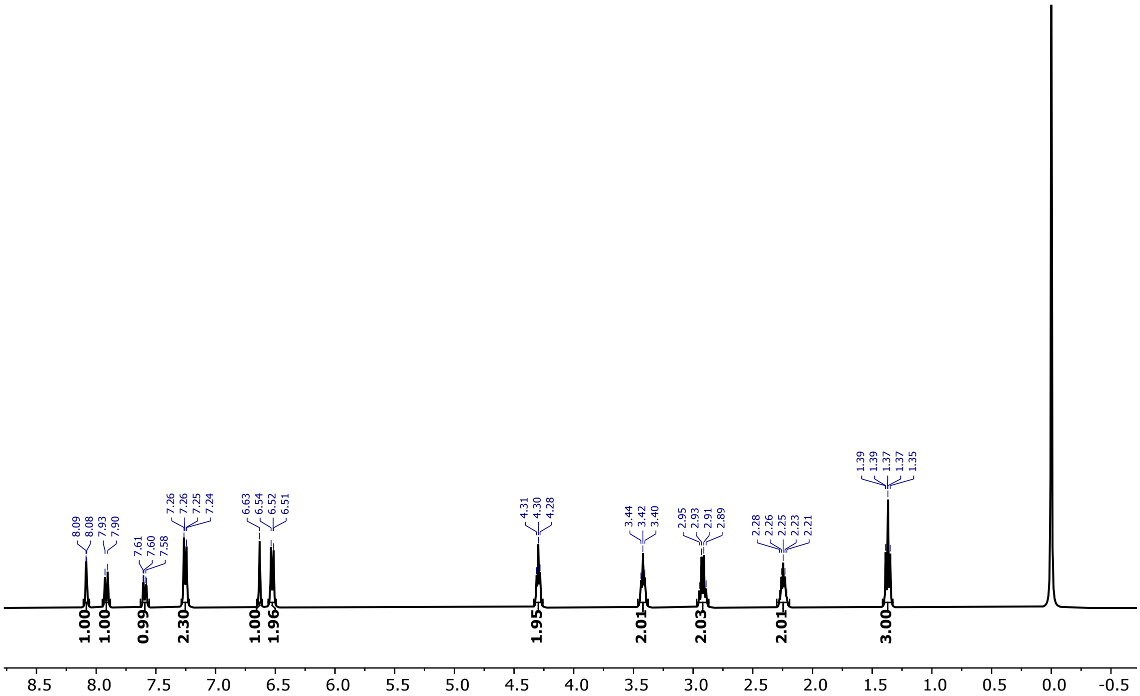

Figure 40. ^{13}H NMR (400 MHz, $CDCl_3$) spectra of compound **8v**.

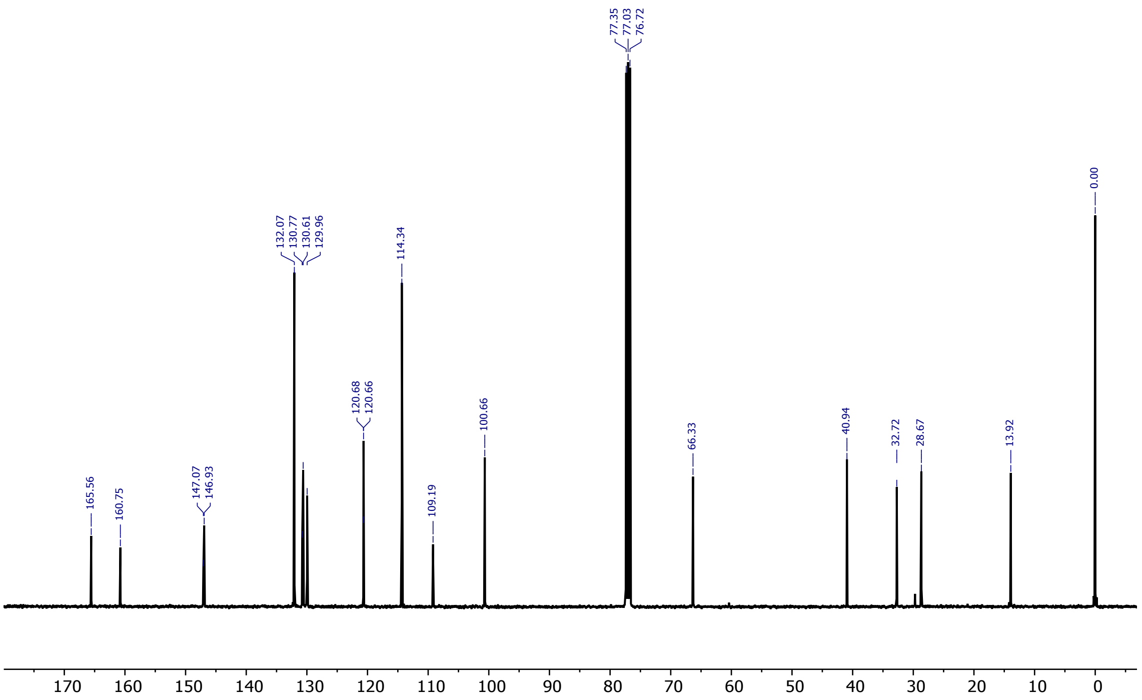

Figure 41. ^{13}C NMR (101 MHz, $CDCl_3$) spectra of compound **8v**.

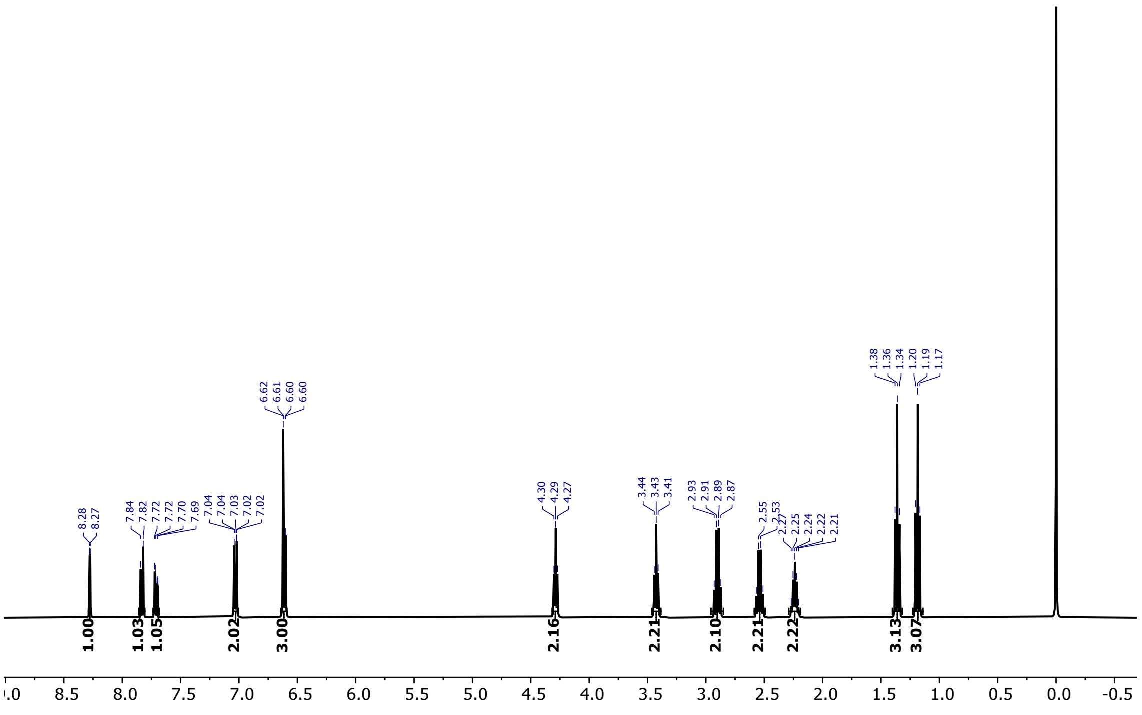

Figure 42. ^{1}H NMR (400 MHz, $CDCl_3$) spectra of compound **8w**.

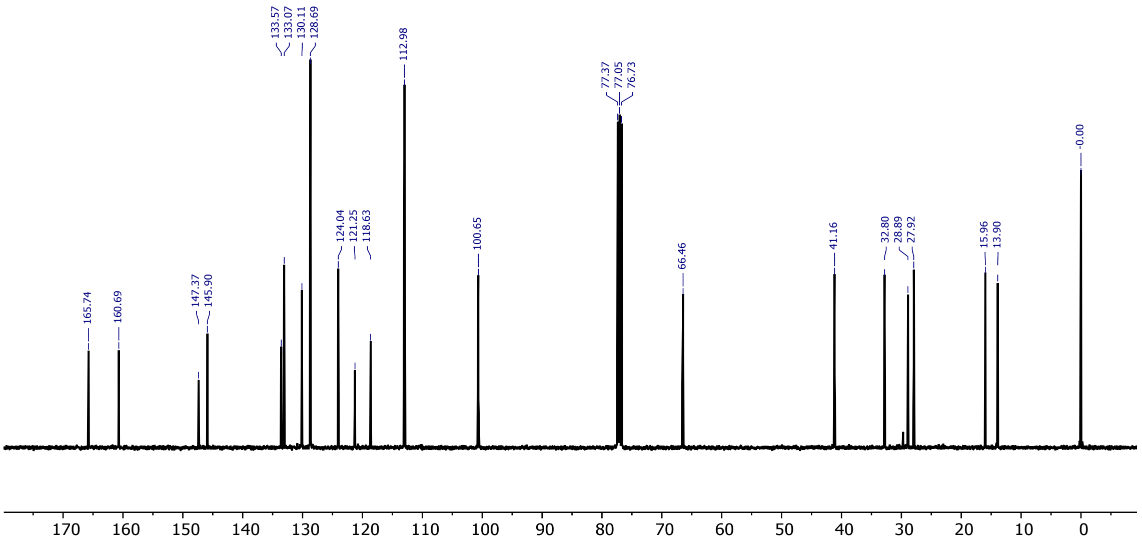

Figure 43. ^{13}C NMR (101 MHz, $CDCl_3$) spectra of compound **8w**.
